# Enzymatic Synthesis of Chemical Nuclease Triplex-Forming Oligonucleotides with Gene-Silencing Applications

**DOI:** 10.1093/nar/gkac438

**Published:** 2022-05-30

**Authors:** Bríonna McGorman, Nicolò Zuin Fantoni, Sinéad O’Carroll, Anna Ziemele, Afaf H El-Sagheer, Tom Brown, Andrew Kellett

**Affiliations:** School of Chemical Sciences and National Institute for Cellular Biotechnology, Dublin City University, Glasnevin, Dublin 9, Ireland; Chemistry Research Laboratory, University of Oxford, South Parks Rd, Oxford, UK; School of Chemical Sciences and National Institute for Cellular Biotechnology, Dublin City University, Glasnevin, Dublin 9, Ireland; School of Chemical Sciences and National Institute for Cellular Biotechnology, Dublin City University, Glasnevin, Dublin 9, Ireland; Chemistry Research Laboratory, University of Oxford, South Parks Rd, Oxford, UK; Department of Science and Mathematics, Suez University, Faculty of Petroleum and Mining, Engineering, Suez 43721, Egypt; Chemistry Research Laboratory, University of Oxford, South Parks Rd, Oxford, UK; School of Chemical Sciences and National Institute for Cellular Biotechnology, Dublin City University, Glasnevin, Dublin 9, Ireland; SSPC, the Science Foundation Ireland Research Centre for Pharmaceuticals, School of Chemical Sciences, Dublin City University, Glasnevin, Dublin 9, Ireland

## Abstract

Triplex-forming oligonucleotides (TFOs) are short, single-stranded oligomers that hybridise to a specific sequence of duplex DNA. TFOs can block transcription and thereby inhibit protein production, making them highly appealing in the field of antigene therapeutics. In this work, a primer extension protocol was developed to enzymatically prepare chemical nuclease TFO hybrid constructs, with gene-silencing applications. Click chemistry was employed to generate novel artificial metallo-nuclease (AMN)-dNTPs, which were selectively incorporated into the TFO strand by a DNA polymerase. This purely enzymatic protocol was then extended to facilitate the construction of 5-methylcytosine (5mC) modified TFOs that displayed increased thermal stability. The utility of the enzymatically synthesised di-(2-picolyl)amine (DPA)-TFOs was assessed and compared to a specifically prepared solid-phase synthesis counterpart through gel electrophoresis, quantitative PCR, and Sanger sequencing, which revealed similar recognition and damage properties to target genes. The specificity was then enhanced through coordinated designer intercalators—DPQ and DPPZ—and high-precision DNA cleavage was achieved. To our knowledge, this is the first example of the enzymatic production of an AMN-TFO hybrid and is the largest base modification incorporated using this method. These results indicate how chemical nuclease-TFOs may overcome limitations associated with non-molecularly targeted metallodrugs and open new avenues for artificial gene-editing technology.

## Graphical Abstract

**Graphical Abstract:**
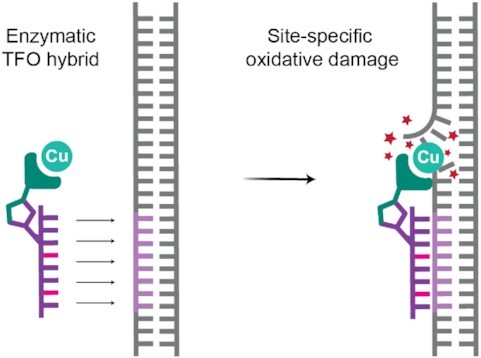
A chemical nuclease triplex-forming oligonucleotide hybrid was enzymatically synthesised, and it was shown to selectively bind with its target DNA sequence and inflict site-specific oxidative DNA damage.

## INTRODUCTION

There is major interest in targeting the human genome with directed nucleases for genetic engineering and knockout applications. A nuclease is an enzyme that can cleave DNA into its individual components and they have been exploited in several breakthrough technologies, such as zinc finger nucleases (ZFNs), (1) transcription activator-like effector nucleases (TALENs), (2) and clustered regularly interspaced short palindromic repeats (CRISPR), (3) with CRISPR/Cas9 now considered the gold standard for gene editing. Synthetic chemical nucleases offer an intriguing addition to these technologies as they provide greater flexibility and offer multiple avenues for gene silencing—including oxidative strand scission. Here, we present the first example of an enzymatically synthesised chemical nuclease-TFO hybrid and demonstrate its utility as a targeted gene knockout agent.

Artificial metallo-nucleases (AMNs) were first reported by Sigman *et al.* when it was found that [Cu (1,10-phenantroline)_2_]^2+^ (Cu-Phen) could semi-intercalate and oxidise DNA from the minor groove in the presence of a reductant. (4,5) Since then the biological activity of this broad class of compounds has been intensively explored with promising anticancer, (6–9) antiviral, (10–12) and antimicrobial (13) chemotypes discovered. This group has reported a variety of AMNs including Cu^2+^ phenanthroline-phenazine complexes (14,15) and, more recently, polypyridyl copper-caging AMN architectures that accommodate designer intercalators. (16,17) In this work, an azide functionalised polypyridyl di-(2-picolyl)amine (DPA) ligand was used to form the Cu-AMN. Copper complexes with this ligand previously showed superior DNA binding and cleaving activity (17) compared to a more sterically crowded tris-(2-pyridylmethyl)amine (TPMA) caging scaffold. (16) One of the major limitations of synthetic nucleases—including Cu(II) complexes of DPA and TPMA—is their promiscuity as stand-alone compounds, causing random cleavage (or shearing) of DNA. Extensive research has therefore been performed to direct AMNs to specific genetic elements. Initial research into sequence specific targeting focused on the covalent linkage of Fe-EDTA or Cu-Phen complexes to distamycin derivatives, (18–22) but to achieve greater precision, research shifted towards the use of oligonucleotides as targeting vectors. Dervan, (23,24) Sigman (25–27) and Hélène (28,29) were the first to demonstrate the attachment of Fe-EDTA or Cu-Phen DNA cleavage agents to oligonucleotides. These hybrids showed great promise as targeted therapeutics, but as nucleic acid chemistry was in its infancy, often complicated and labour-intensive protocols were required during their preparation, which ultimately precluded further development. In recent years, research into the tethering of metallodrugs to oligonucleotides has re-surfaced. For example, in 2012 Miller *et al.* (30) reported a family of platinum-derivatised oligonucleotides that were capable of binding to their target sequence, while the *trans*-platinum(II) moiety cross-linked the DNA. (30) However, the inclusion of platinum-derivatised nucleotides was challenging, which stunted their development. Komiyama *et al.* reported an alternative strategy that overcomes some of the challenges associated with tethering a DNA damaging agent to a nucleic acid delivery vector. (31) This system, known as artificial restriction DNA cutters (ARCUT), is composed of two pseudo-complementary PNA (pcPNA) strands with a Ce(IV)/EDTA nuclease. (31–33) However, this system is compatible with nucleases that can discriminate between double and single-stranded DNA.

Therefore, there was still a desire to covalently attach metallodrugs to nucleic acid delivery vectors. The advent of click chemistry (34) provided a breakthrough synthetic strategy for this task, and recently the ‘click’ attachment of metallodrugs to antigene triplex-forming oligonucleotides (TFOs) has been successful. (35–38) TFOs are short single stranded oligonucleotides that can hybridise with duplex DNA and inhibit protein production at the genomic level. (39) Therefore, AMN-TFO hybrids produced using click chemistry are of significant interest as the TFO sequence can be adapted to new genetic targets, and the desired AMN cleavage unit can be attached to the TFO through simple ‘click’ ligation. There is also significant interest in the development of TFOs with increased stability and various modifications that increase the melting temperature of TFOs have been reported. These include artificial bases, such as 5-methyl-cytosines (5mC), (40) pseudo-deoxycytidine (ΨdC), (41) and 6-amino-5-nitropyridin-2-one (Z) (42), and DNA intercalating agents, for example thiazole orange (35,43) and modified phenanthrene ligands (36). The inclusion of one or more of these modifications enhances the formation of stable triple helical DNA under physiological conditions, and thereby increases their utility. The introduction of a methyl group to the C5 position of cytosine is one of the simplest modifications, and the inclusion of 5mC bases in a TFO sequence can increase triplex stability and reduce the pH dependence of pyrimidine TFOs. This is due to 5mC bases having a higher p*K_a_*, disrupting the surrounding water structure, and enabling greater base stacking and/or hydrophobic interactions in the major grove. (40,44,45)

**Figure 1.**
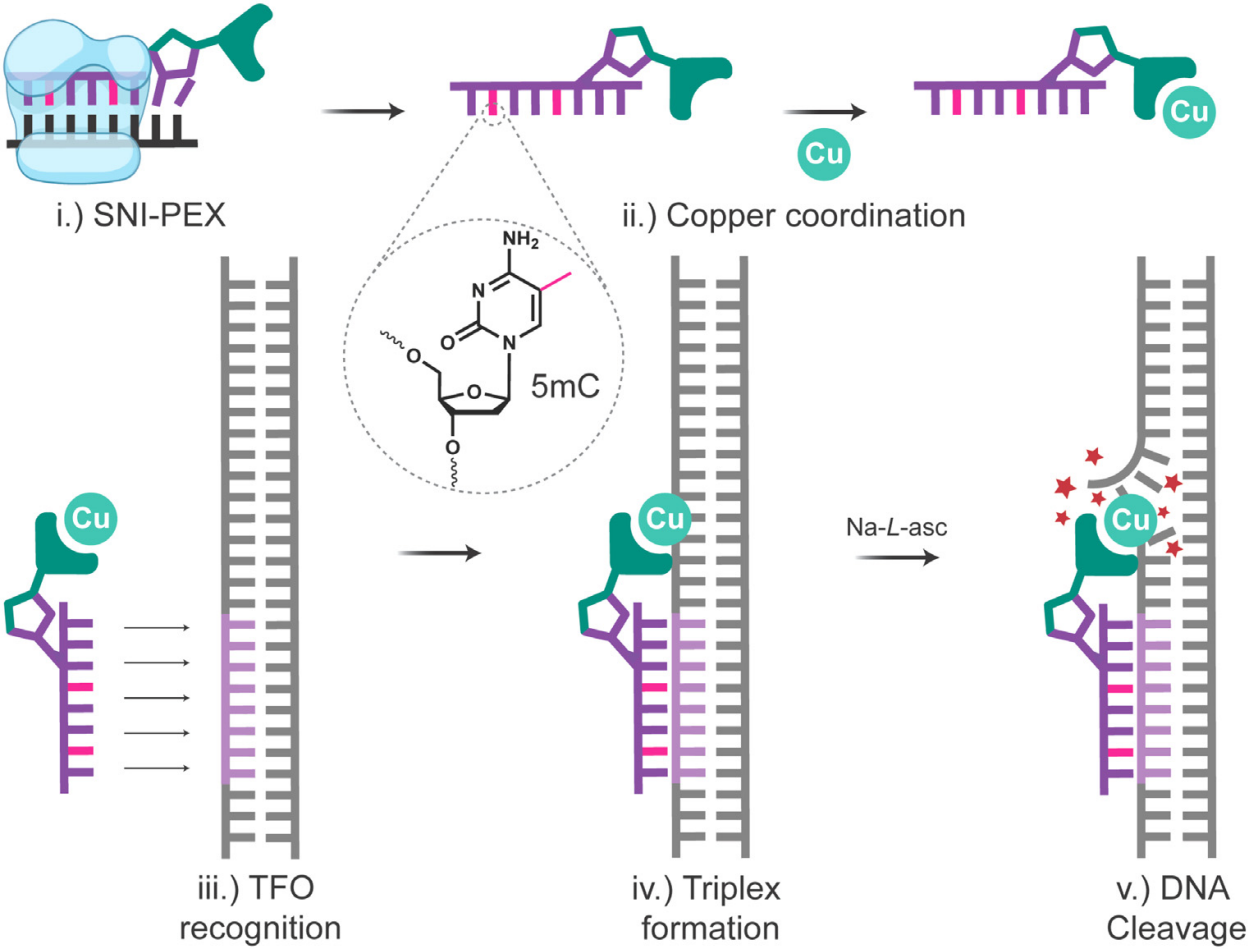
Enzymatic synthesis concept for the 5mC-DPA-TFO hybrid and proceeding steps for targeted DNA cleavage: i.) DNA polymerase synthesises DPA-TFO; ii.) Cu(II) coordinates to the DPA ligand; iii.) Cu(II)-DPA-TFO recognises target duplex; iv.) TFO hybridises to duplex forming triple helical DNA; and v.) Na-*L*-ascorbate activates the AMN to trigger DNA cleavage.

Solid-phase synthesis (SPS) (46,47) is widely used for the production of short oligonucleotides but it is not compatible with modifications that are susceptible to oxidation or are reactive with nucleophiles. (48) This limits the potential functionality of SPS in the production of oxidatively active antigene therapeutics, as the AMN unit must be post-synthetically attached to the TFO. Nucleic acid click chemistry (49,50) offers a relatively simple way to achieve this. (35–38,51) However, the increasing number of therapeutic oligonucleotides approved for clinical use emphasises the need for alternative and greener synthetic strategies. In this work a new enzymatic method for the *in-situ* preparation of an entire oxidative gene-editing probe is reported. Here, nucleic acid click chemistry was combined with a modified primer extension (PEX) protocol—known as single nucleotide incorporation-primer extension (SNI-PEX) (52)—to enzymatically prepare a novel DPA-TFO hybrid (Figure 1). The enzymatic synthesis of DNA *in vitro* is not a new phenomenon and the use of polymerases to synthesise modified DNA was first reported by Langer *et al.* in 1981. (53) However, it was not until 2013 when the Hocek group reported the SNI-PEX protocol (52) that the enzymatic synthesis of AMN-TFO hybrids became a possibility. Recently, the PEX synthesis of oligonucleotides bearing crosslinking agents (54), redox labels (55) and fluorophores (56) have been reported, indicating the expanding possibilities of this technology. In the current work, the copper-catalysed azide-alkyne cycloaddition (CuAAC) reaction (57,58) was employed to generate novel DPA-modified nucleotide triphosphates, which were incorporated into the TFO sequence using a DNA polymerase and employed as gene-editing tools without purification steps. The sequence selectivity and targeted DNA damaging capabilities of these PEX DPA-TFO hybrids was then studied using a variety of molecular and biophysical methods, and their functionality was compared to a SPS post-synthetic ‘click’ DPA-TFO counterpart, specifically prepared for this study. The SNI-PEX method was then extended to generate higher-order hybrids containing designer phenazine ligands—including DPPZ (59,60)—or heavily modified 5mC sequences.

## MATERIALS AND METHODS

### General remarks

PAGE gels were cast using a SureCast^TM^ gel casting system (Invitrogen) and run in an Invitrogen mini gel tank. Agarose gel electrophoresis was performed using a Bio-Rad wide-cell mini sub system. A Bio-Rad basic Power Pac^TM^ was used for PAGE and agarose gel electrophoresis. All gels were imaged using a Syngene G:Box 9 mini gel documentation system. Band densitometry was performed using GeneTools software (Syngene). PCR and triplex annealing were performed using a Mastercycler^®^ nexus (Eppendorf). Real-time PCR (qPCR) and fluorescence thermal melting were performed on a Roche LightCycler 480 II using SYBR green I master mix (Roche) and adhered to the MIQE guidelines. BioRender was used to generate the DNA polymerase graphic.

### Synthesis of N-4-azidobenzyl-N-di-(2-picolyl) amine (4N_3_-Benzyl-DPA)

#### N-4-nitrobenzyl-N-di-(2-picolyl)amine (1)

On a parallel synthesiser, ten reactions were prepared using a modification of a literature procedure reported by Du *et al.* (61) DPA (0.247 g, 1.24 mmol), K_2_CO_3_ (0.516 g, 3.74 mmol) and *p*-nitrobenzylbromide (0.268 g, 1.24 mmol) were mixed in CH_3_CN (10 mL) and stirred overnight at room temperature. The resulting suspension was filtered and the solvent removed to yield an orange oil. The crude product was purified by column chromatography (silica gel, CH_3_CN:CH_2_Cl_2_, gradient 0:100 to 30:70 v/v, followed by MeOH: CH_2_Cl_2_, gradient 5:95 to 20:80 v/v) yielding **1** as a brown solid (3.706 g, yield = 89.4%). ^1^H-NMR (600 MHz, CDCl_3_): δ 8.54 (dq, J = 4.9, 1.7, 0.9 Hz, 2H), 8.16 (dt, J = 8.8, 2.3 Hz, 2H), 7.67 (td, J = 7.7, 1.8 Hz, 2H), 7.58 (d, J = 8.8 Hz, 2 H), 7.52 (d, J = 7.8 Hz, 2H), 7.17 (qd, J = 7.4, 4.9, 1.1 Hz, 2H), 3.83 (s, 4H), 3.81 (s, 2H).^13^C-NMR (151 MHz, CDCl_3_): δ 159.0, 149.3, 147.3, 136.7, 129.5, 123.7, 123.1, 122.4, 60.3, 57.9. ESI-MS *m/z* calcd: 357.1 [M + Na]^+^; found: *m/z* 358.1 (Supplementary section S-1; Supplementary Figure S-1–S-4).

#### N-4-amminobenzyl-N-di-(2-picolyl)amine (2)

To a solution of **1** (0.497 g, 1.49 mmol) in EtOH/H_2_O (3:1; 48 mL), Na_2_S (1.054 g, 13.50 mmol) was added and stirred at 70°C for two days. The solvent was reduced and the product extracted with CH_2_Cl_2_ (3 × 50 mL). The combined fractions were dried over MgSO_4_ and the solvent removed to afford **2** as an orange oil that solidifies over time (0.371 g, yield = 82.0%). ^1^H-NMR (600 MHz, CDCl_3_): δ 8.50 (d, J = 4.8 Hz, 2H), 7.65 (td, J = 7.9, 1.4 Hz, 2H), 7.57 (d, J = 7.8 Hz, 2H), 7.18 (d, J = 8.2 Hz, 2 H), 7.12 (q, J = 6.2, 5.1 Hz, 2H), 6.63 (d, J = 8.2 Hz, 2H), 3.77 (s, 4H), 3.55 (s, 2H).^13^C-NMR (151 MHz, CDCl_3_): δ 160.2, 149.0, 145.5, 136.5, 130.2, 128.9, 122.9, 122.0, 115.2, 59.9, 58.1. ESI-MS: *m/z* calcd: 372.2 [M + Na]^+^; found: 372.1 (Supplementary section S-1; Supplementary Figure S-5 – S-8).

#### N-4-azidobenzyl-N-di-(2-picolyl)amine (3)

The diazotransfer reagent 2-azido-1,3-dimethylimidazolinium hexafluorophosphate (ADMP) used in the following synthesis was prepared according to a literature procedure previously reported by Kitamura *et al.* (62) To a suspension of *N*-4-amminobenzyl-*N*-*di*-(2-picolyl)amine (0.159 g, 0.52 mmol) and *N,N*′-dimethyl-4-aminopyridine (DMAP; 0.076 g, 0.63 mmol) in CH_3_CN (10 mL), ADMP (0.178 g, 0.63 mmol) was added and stirred overnight at 30°C. The reaction was quenched with a saturated aqueous solution of NaHCO_3_ and the salted-out product extracted with CH_2_Cl_2_ (3 × 20 mL). The combined fractions were dried over MgSO_4_ and the solvent removed. The crude compound was purified by column chromatography (silica gel, CH_3_CN:CH_2_Cl_2_, gradient 40:60 to 0:100 v/v, followed by MeOH:CH_2_Cl_2_, gradient 0:100 to 20:80 eluting the product). The combined fractions were dried *in vacuo* to afford the product **3** as a brown solid (0.121 g, yield = 70%). ^1^H-NMR (600 MHz, CDCl_3_): δ 8.52 (dq, J = 4.9, 1.8, 0.9 Hz, 2H), 7.66 (td, J = 7.7, 1.8 Hz, 2H), 7.55 (d, J = 7.8 Hz, 2H), 7.38 (d, J = 8.5 Hz, 2 H), 7.15 (qd, J = 7.4, 4.9, 1.1 Hz, 2H), 6.98 (dt, J = 8.5, 2.5 Hz, 2H), 3.79 (s, 4H), 3.66 (s, 2H). 13C-NMR (151 MHz, CDCl3): δ 149.2, 136.6, 130.4, 123.0, 122.2, 119.1, 60.0, 57.9. ESI-MS: *m/z* calcd: 353.1 [M + Na]^+^; found: 353.1 (Supplementary section S-1; Supplementary Figure S-9 – S-12).

### Solid-phase synthesis of DPA-TFO

#### TFO sequence

An internally modified 31 nucleotide poly-pyrimidine parallel-binding TFO was produced by solid-phase synthesis in a similar manner to earlier reported TPMA-TFOs. (35) This TFO was selected as previous studies have reported thermal stability > 37°C (35,38) and increased target discrimination for internally modified AMN-TFO hybrids. (35)

#### Solid-phase synthesis of alkyne-modified TFO

The TFO with an internal octadiynyl-dU (Glen Research) was synthesised by solid-phase phosphoramidite synthesis using an automated DNA synthesiser (Applied Biosystems 394) according to standard protocols. Coupling efficiencies were monitored by the trityl cation conductivity monitoring facility and were > 98%. The TFO was deprotected and cleaved in standard aqueous ammonia by heating to 55°C for 5 hours, and purified by gradient reverse phase HPLC (C8 size exclusion column) in 0.1 M triethylammonium acetate (TEAA) buffer with 50% CH_3_CN. A gradient of 10–40% over 20 minutes was used with a 4 mL/min flow rate before desalting using a Sephadex^TM^ G-25 grade gravity filtration column (NAP-25^TM^, GE Healthcare). The TFO was then analysed by ESI-MS.

#### Click chemistry reaction of octadiynyl-TFO and 4N_3_-benzyl-DPA

The copper(I)-catalysed azide-alkyne cycloaddition (CuAAC) reaction used to ‘click’ the alkyne-modified TFO with 4N_3_-benzyl-DPA was performed according to the Lumiprobe protocol for click-chemistry labelling of oligonucleotides with minor modifications. The internal-octadiynyl-TFO (60 nmol), 4N_3_-benzyl-DPA (90 nmol), and Na-*L*-ascorbate (1.5 μmol) were mixed in a 50:50 solution of DMSO:water. The solution was degassed with nitrogen and pre-complexed Cu-TBTA (1.5 μmol) was added. The reaction mixture was further degassed and stirred overnight, before being quenched with EDTA (30 μmol) and desalted using Sephadex™ G-25 grade gravity filtration columns (NAP-25^™^, GE Healthcare). The DPA-TFO was then purified by gradient (0.1 M NH_4_OAc + 50% CH_3_CN in NH_4_OAc) reverse phase HPLC (C8 size exclusion column), and the yield was calculated (Supplementary Table S-1). The DPA-TFO was then analysed by ESI-MS (Supplementary section S-2; Supplementary Table S-2; Supplementary Figure S-13).

### Enzymatic synthesis of DPA-TFO

#### TFO sequence

A 32 nt TFO with sequence homology to the SPS DPA-TFO hybrid was selected for enzymatic synthesis. The SPS and SNI-PEX TFOs would ideally have identical sequences, but it was necessary to shift the modification position one base towards the 3′ end of the TFO due to the 5′ to 3′ nature of enzymatic synthesis which otherwise would result in double incorporation of dU^DPA^.

#### Production of DPA-dUTP through click chemistry

In an anaerobic environment, a copper-catalysed azide-alkyne cycloaddition (CuAAC) reaction mixture of C8-alkyne-dUTP (5 nmol, BaseClick), 1.5 equivalents 4N_3_-benzyl-DPA (7.5 nmol), Na-*L*-ascorbate (25 nmol) and Cu-TBTA (25 nmol) was prepared in water and DMSO (1:1) to a total volume of 250 μL. The reaction proceeded at 25°C and 450 RPM for 6 hours and was quenched with 500 equivalents of EDTA and stored at −20°C. A sample of dU^DPA^TP was analysed by ESI-MS (Supplementary section S-3; Supplementary Figure S-14 and 15).

#### SNI-PEX template and primer design

SNI-PEX primers and template were carefully designed to enable the synthesis of a TFO bearing a single DPA modification. The template had a 5′ biotin modification and was fully complementary to the desired TFO sequence (supplementary section Table S-3). Primers with all natural bases and 5-methyl-cytosines (5mC) were carefully selected so that only a single dU^DPA^TP would be incorporated. Therefore, it was necessary for the primer sequence to end at a position followed by a single 3′ ‘T’ incorporation site (*i.e*. one ‘A’ was present on the complementary template strand). If two ‘T’ incorporation sites followed the primer then two dU^DPA^TPs would be incorporated, and an alternative synthetic protocol would be required for the inclusion of a single modified base. (52)

#### SNI-PEX and streptavidin magnetic particles

In a total volume of 200 μL, a sample containing; primer (17 nt, 10 μM, 27 μL, supplementary section Table S-3), 5′-biotinylated template (32 nt, 10 μM, 27 μL), dU^DPA^TP (13.3 μM, 30.4 μL), MgSO_4_ (100 mM, 8 μL, NEB) and Vent (exo-) DNA polymerase (2 U/ μL, 10 μL, NEB) in ThermoPol buffer (10 x, 20 μL, NEB) was prepared. This was incubated at 37°C and 450 RPM for 90 min. Natural dNTPs (10 mM, 27 μL, NEB) were added and a further 20 min incubation at 60°C and 450 RPM was performed, before cooling to 4°C to stop the reaction. The above procedure was repeated using a primer containing 5-methyl cytosines (17nt, supplementary section Table S-3) and 2′-deoxy-5-methylcytidine 5′-triphosphates (NEB) to synthesise a TFO containing methyl-cytosines.

Streptavidin magnetic particles (100 μL, Roche) were washed three times with TEN_100_ (200 μL; 10 mM Tris, 1 mM EDTA, 100 mM NaCl, pH 7.5), and resuspended in TEN_100_ (50 μL). SNI-PEX solution (200 μL) was added and incubated at 18°C and 1200 RPM for 20 min. Particles were collected on a magnet and solution was discarded. Particles were washed three times with TEN_1000_ (200 μL; 10 mM Tris, 1 mM EDTA, 1 M NaCl, pH 7.5), and then washed four times with elution buffer (200 μL; 100 mM sodium pyrophosphate). Particles were resuspended in elution buffer (100 μL), and double stranded DNA was denatured (65°C for 90 sec). Particles were collected on a magnet and the solution, containing the single stranded TFO, was collected. TFO was de-salted using Sephadex^TM^ G-25 grade gravity filtration columns (NAP-5^TM^, Cytiva illustra^TM^). DPA-TFO was quantified by UV fluorescence and concentration was calculated using ϵ_260_ values (Nanodrop 1000, Thermofisher). The product yield was calculated (supplementary section Table S-4) and TFOs were analysed by ESI-MS (supplementary section Table S-5).

#### Denaturing PAGE to visualise single nucleotide incorporation

20% denaturing polyacrylamide gels (1x TBE buffer, 7M urea) were cast. Denaturing loading buffer (95% formamide, 18 mM EDTA, 0.025% SDS, xylene cyanol, bromophenol blue, Invitrogen) was added to SNI-PEX samples (10 μL) and heated to 95°C for 4 min. Samples were loaded directly onto the gel and run at 150 V for 160 min in a Surecast mini gel tank (Invitrogen). The gel was post-stained with SYBR gold and visualised on a G:Box 9 mini (Syngene) gel documentation system. Band densitometry was performed in triplicate on raw gel images.

### Analysis of sequence specific cleavage by DPA-TFO

#### Amplification of TFO target and off-target

A 57 bp amplicon containing the TFO recognition sequence (pCSanDI-HYG; forward: 5′-AAGCCGGCGAACGTGGCGG-3′ and reverse: 5′-CCTAGCGCCCGCTCCTTTCG-3′), and a 40 bp amplicon lacking the TFO target sequence (pUC19, NEB; forward: 5′-TGACTCCCCGTCGTGTAGAT-3′ and reverse: 5′-AGCCCTCCCGTATCGTAGTT-3′) were generated by PCR (MyTaq Red DNA polymerase, Bioline) and purified into 6 μL of nuclease-free water using membrane spin columns (Monarch PCR & DNA clean-up kit, NEB). These are the 57 bp target and 40 bp *off*-target duplexes (see supplementary section Table S-6).

#### Fluorescent thermal melting

This protocol was adapted from reference (63). The TFO was hybridised to its dsDNA target using two different methods to ensure triplex was successfully formed: (i) TFO (25 pmol) and 57 bp target duplex (5 pmol) were incubated in phosphate buffer (10 mM PO_4_^3−^, 150 mM NaCl, 2 mM MgCl_2_, pH 6.1, or 10 mM PO_4_^3−^, 150 mM NaCl, 2 mM MgCl_2_, pH 7) at 37°C for 15 hours; (ii) In phosphate buffer (10 mM PO_4_^3−^, 150 mM NaCl, 2 mM MgCl_2_, pH 6.1, or 10 mM PO_4_^3−^, 150 mM NaCl, 2 mM MgCl_2_, pH 7) TFO (25 pmol) and 57 bp target duplex (5 pmol) were annealed by heating to 95°C and slowly cooling (1 °C/min) to 4°C. Both methods resulted in successful triplex formation. SYBR green I (1 μL, Roche) was added to each sample and the melting profile for the triplex was analysed on a LightCycler^®^ 480 II (Roche). Samples were heated to 99°C (0.1°C/sec) with 10 fluorescence measurements recorded per °C. A plot of sample temperature versus fluorescence was obtained and the first negative derivative of the sample was calculated, resulting in the melting temperature being displayed as a peak.

#### Triplex formation

Target (57 bp, 1 pmol) and *off*-target (40 bp, 1 pmol) duplexes were incubated with 10, 25 and 50 equivalents of TFO (32 nt) in phosphate buffer (10 mM PO_4_^3−^, 150 mM NaCl, 2 mM MgCl_2_, pH 6.1) at 37°C for 24 hours. 6X loading dye (10 mM Tris-HCl, pH 7.6, 0.03% bromophenol blue, 0.03% xylene cyanol FF, 60% glycerol, 60 mM EDTA, Invitrogen) was added, and the entire solution was loaded onto a native PAGE gel (50 mM Tris acetate, 75 mM NaCl, 5 mM MgCl_2_, pH 6.1). Gel was run at 70 V for 240 min and was post-stained with SYBR gold.

#### DPA-TFO hybrids PAGE

The DPA-TFO hybrid (15.625 pmol) was incubated with 1 equivalent of Cu(ClO_4_)_2_ for 15 min at room temperature. This allowed Cu(II) to coordinate with DPA. The copper-bound hybrid (15.625 pmol), and Na-*L*-ascorbate (25, 250 or 1000 eq.), was then incubated with a duplex containing the TFO recognition site (57 bp fragment of GFP, 0.625 pmol) and *off*-target duplex (40 bp fragment of pUC19, 0.625 pmol) in phosphate buffer (10 mM PO_4_^3−^, 150 mM NaCl, 2 mM MgCl_2_, pH 6.1) at 20, 30 or 40°C for 2, 15 and 24 hours, the reaction was quenched with 6X loading dye (10 mM Tris-HCl, pH 7.6, 0.03% bromophenol blue, 0.03% xylene cyanol FF, 60% glycerol, 60 mM EDTA, Invitrogen). The entire solution was loaded onto a native PAGE gel (50 mM Tris acetate, 75 mM NaCl, 5 mM MgCl_2_, pH 6.1) and was run at 70 V for 240 min. The gel was post-stained with SYBR gold.

#### PAGE analysis of cleavage activity of Cu-DPA-TFO hybrids at physiological temperature

A DPA-TFO hybrid (15.625 pmol) was incubated with 1 equivalent of Cu(ClO_4_)_2_ for 15 min at room temperature, to allow Cu(II) to coordinate with DPA. This Cu-DPA-TFO (15.625 pmol), and increasing concentrations of Na-*L*-ascorbate (0–1000 eq), was then incubated with a duplex containing the TFO recognition site (57 bp fragment of GFP, 0.625 pmol) and *off*-target duplex (40 bp fragment of pUC19, 0.625 pmol) in phosphate buffer (10 mM PO_4_^3−^, 150 mM NaCl, 2 mM MgCl_2_, pH 6.1) at 37°C for 15 hours, the reaction was quenched with 6X loading dye (10 mM Tris-HCl, pH 7.6, 0.03% bromophenol blue, 0.03% xylene cyanol FF, 60% glycerol, 60 mM EDTA, Invitrogen). The entire solution was loaded onto a native PAGE gel (50 mM Tris acetate, 75 mM NaCl, 5 mM MgCl_2_, pH 6.1) and was run at 70 V for 240 min. The gel was post-stained with SYBR gold. Gels were run in triplicate and band densitometry was performed to analyse band intensity.

#### ROS scavengers

Samples for PAGE were prepared as described above, however the *off*-target sequence was excluded and a reactive oxygen species (ROS) scavenger (50 nmol); L-histidine, D-mannitol, L-methionine or 4,5-dihydroxy-1,3-benzendisulfonic acid (tiron) was added prior to incubation at 37°C. The DNA damage inflicted was analysed by PAGE.

#### qPCR analysis of catalytic activity with target and *off*-target

qPCR was performed using SYBR green I fluorescence (see supplementary section S-4.5 for more information on experimental design). A 113 bp amplicon containing the TFO recognition sequence (pCSanDI-HYG; forward: 5′-AAAGGGAGCCCCCGATTTAG-3′ and reverse: 5′-GTGACCGCTACACTTGCCA-3′ see supplementary section Table S-6), and a 116 bp amplicon lacking the TFO target sequence (pUC19, NEB; forward: 5′-TCCGGTTCCCAACGATCAAG-3′ and reverse: 5′-AGTGATAACACTGCGGCCAA-3′ see supplementary section Table S-6) were generated by PCR (MyTaq Red DNA polymerase, Bioline) and purified using membrane spin columns (Monarch PCR & DNA clean-up kit, NEB). In a total volume of 12 μL (10 mM PO_4_^3−^, 150 mM NaCl, 2 mM MgCl_2_, pH 6), the target duplex (113 bp, 1.25 pmol) and *off*-target sequence (116 bp, 1.25 pmol) were exposed to Cu-DPA-TFO or a 1:1 mixture of free Cu-DPA and free TFO (31.25 pmol) in the presence of increasing equivalents of Na-*L*-ascorbate (0–2000 eq.). At t = 0 hours, 6 μL of each reaction volume was quenched with EDTA (60 nmol) and kept as the reference samples. The remaining 6 μL was incubated at 37°C for t = 15 hours (experiment) prior to addition of EDTA. Diluted (1:40^5^) reference and experiment samples were analysed by qPCR over 45 PCR cycles. The changes in C_T_ (threshold cycle) between experiment and reference samples (experiment C_T_ – reference C_T_) were calculated (ΔC_T_). ΔC_T_ was then normalised to a non-treated control (ΔC_T_ sample - ΔC_T_ control = ΔΔC_T_), and this was plotted as a linear value 2^−ΔΔCT^. Two-way ANOVA with Tukey's multiple comparisons was performed, using GraphPad Prism, to determine the significance of the 2^−ΔΔCT^ values obtained. * = *P* = < 0.05, significant ** = *P* < 0.01, very significant. *** = *P* < 0.001, extremely significant.

#### Plasmid Studies

The DPA-TFO hybrid (2.5 pmol) was incubated with 1 equivalent of either: Cu(ClO_4_)_2_; Cu(II)-DPQ (64); or Cu(II)-DPPZ (65) for 15 min at room temperature, to allow Cu(ClO_4_)_2_/ Cu(II)-DPQ / Cu(II)-DPPZ to coordinate with DPA. Plasmid DNA containing the GFP gene (400 ng, pCSanDI-HYG-N44) and the pUC19 plasmid (400 ng, NEB) were then incubated with the 25 equivalents of (DPQ/DPPZ)-Cu(II)-DPA-TFO (2.5 pmol) and increasing concentrations of Na-*L*-ascorbate (25–2000 eq., 62.5 – 5000 pmol) in phosphate buffer (10 mM PO_4_^3−^, 150 mM NaCl, 2 mM MgCl_2_, pH 6.1) at 37°C for 15 hours. The reaction was quenched with 6X loading dye (10 mM Tris-HCl, pH 7.6, 0.03% bromophenol blue, 0.03% xylene cyanol FF, 60% glycerol, 60 mM EDTA, Invitrogen) and loaded onto a 1% agarose gel containing SYBR safe (1X, Invitrogen). The gel was run at 50 V for 270 min, and imaged using UV fluorescence.

#### Sequencing

The GFP plasmid (2.25 mg, pCSanDI-HYG-N44) was incubated with 25 equivalents of (DPQ/DPPZ)-Cu(II)-DPA-TFO (14 pmol) and Na-*L*-ascorbate (2000 eq., 28 nmol) in phosphate buffer (10 mM PO_4_^3−^, 150 mM NaCl, 2 mM MgCl_2_, pH 6.1) at 37°C for 15 hours. The reaction was quenched with EDTA (500 eq., 7 nmol) and purified into 15 μL of nuclease-free water using membrane spin columns (Monarch PCR & DNA clean-up kit, NEB). Samples were subjected to Sanger sequencing (Eurofins) with forward (5′-CGGTGGATGTGGAATGTGTG-3′) and reverse primers (5′-CTGAGTAGGTGTCATTCTATTCTG-3′), and data was analysed using SnapGene. It is important to note that the sequencing information obtained is complementary to the DNA strand read during Sanger sequencing. Thus, the undefined ‘X’ region read on the extended reverse primer (i.e. pyrimidine-rich sequence) indicates damage on the complementary (i.e. poly-purine) sequence of the plasmid that is cleaved by the DPA-TFO hybrids.

## RESULTS AND DISCUSSION

### Synthesis of 4N_3_-Benzyl-DPA

**Figure 2.**
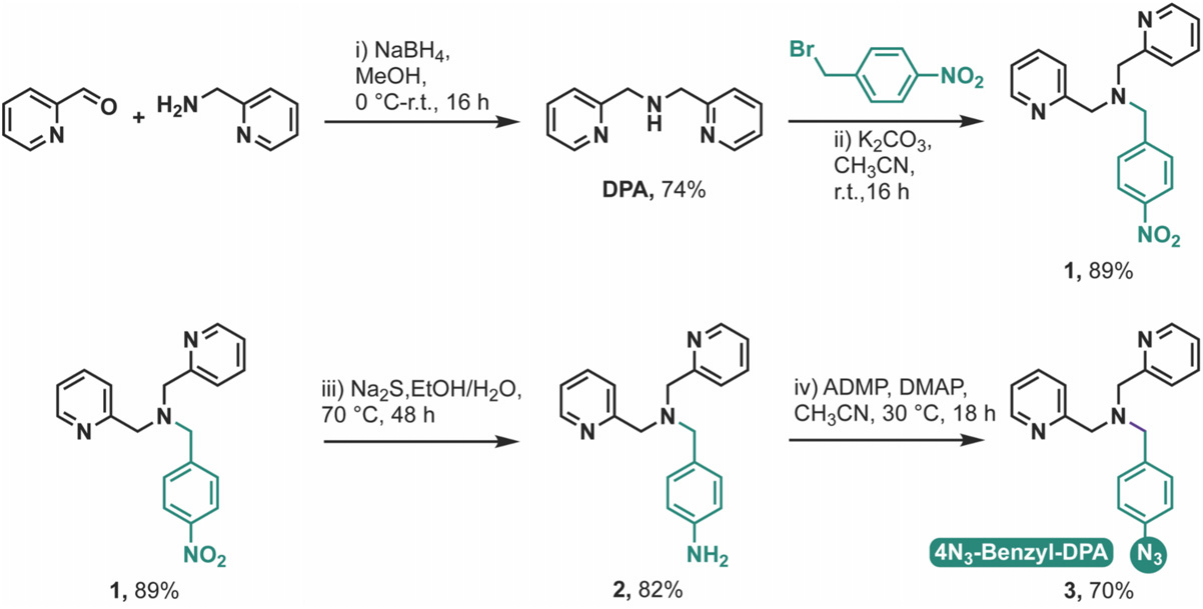
Schematic for the synthesis of 4N_3_-benzyl-DPA.

The polypyridyl copper caging ligand, DPA, was selected for this study as recent work into copper complexes of TPMA (16) and DPA (17) revealed the less sterically hindered DPA ligand had a superior DNA binding affinity. Therefore, within this work a synthetic route to introduce an azide-modification into the copper binding polypyridyl DPA ligand was developed (Figure 2). The synthetic strategy was designed with the aim of: i.) using canonical ‘click’ reaction conditions (modularity, insensitivity toward oxygen and water, regiospecificity and stereospecificity); ii.) green chemistry considerations (employment of safer solvents, chemicals, and easily removable catalysts or reactants); and iii.) enabling potential industrial up-scalability. DPA was treated with 4-nitrobenzyl bromide to form **1**. The azide group was introduced by reducing the NO_2_ group to an amine followed by a diazotransfer reaction on the N-4-amminobenzyl linker of **2** (Figure 2). The use of NaS_2_ in the nitro- to amine-reduction provided an alternative pathway avoiding the requirement for potentially pyrophoric and oxygen sensitive conditions such as those typical of palladium on carbon hydrogenations. Similarly, the use of 2-azido-1,3-dimethylimidazolinium hexafluorophosphate (ADMP) provided a safer alternative to sodium azide, which is the common reactant in canonical azidation reactions.

### Enzymatic synthesis of DPA-TFO hybrids

**Figure 3.**
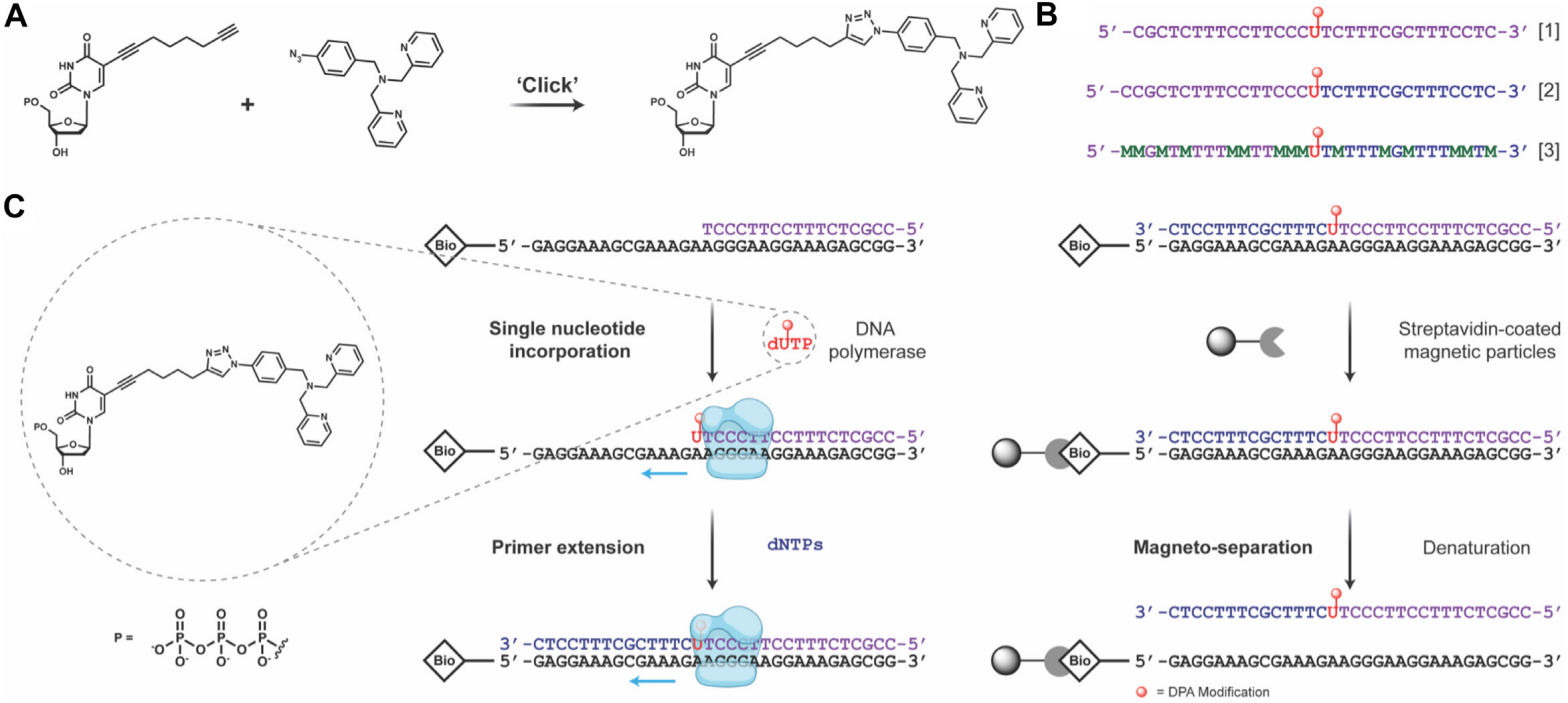
(**A**) Scheme of click reaction between C8-alkyne-dUTP and 4N_3_-benzyl-DPA to generate dU^DPA^TPs. (**B**) Sequence of SPS DPA-TFO1, SNI-PEX DPA-TFO2 and SNI-PEX DPA-5mC-TFO3, where M = 5mC. (**C**) Template (black)—complementary to the desired TFO sequence—and a short primer (purple)—that will form the start of the TFO—are annealed. dU^DPA^TP (red) is then incorporated by a DNA polymerase (blue) immediately 3′ of the primer. The remainder of the sequence is then synthesised with natural dNTPs (dark blue). For an oligonucleotide to behave as a TFO it must be single stranded, therefore the template and newly synthesised TFO sequence must be separated. Streptavidin coated magnetic particles bind to the biotin-tag on the template, then using a magnet and a denaturation step the TFO can be isolated.

A SNI-PEX method was developed to enable the incorporation of dNTPs bearing large modifications, such as the DPA ligand (Figure 3). The first step for this enzymatic synthesis was to generate DPA-modified uridine triphosphates (dU^DPA^TPs) (Figure 3A). The Cu(I)-catalysed azide-alkyne cycloaddition (CuAAC) reaction (57,58) was selected for this task as it can be performed rapidly, at room temperature, and in either organic solvents or aqueous media. This enabled the reaction between the 4N_3_-benzyl-DPA and C8-alkyne-dUTP (dU^a^TP) to be performed within 6 hours without any specialised equipment and in a system that was compatible with the next step of the synthetic protocol, alleviating the need for a purification step. The click reaction was quenched with EDTA and the newly formed dU^DPA^TPs were ready to be incorporated into the TFO strand. A sample of the dU^DPA^TPs was removed, purified by HPLC, and analysed by mass spectrometry to ensure the desired product was present (Supplementary Figure S-14 and S-15).

A poly-pyrimidine parallel-binding TFO was selected for this study (Figure 3B). This TFO was designed to target a 32 base pair region of the eGFP gene (pCSanDI-HYG). The targeted region contains a low number of consecutive C^+^-GC triplets as they are only stable at low pH (66,67) and contains a minimum number of triplet inversion sites (*i.e*. G-CG) to prevent reduced triplex stability (Supplementary Table S-3) (68). Although the selected TFO contains a low number of G-CG inversions, T-CG or C-CG sites would also have been viable options to further increase stability. (69) In previous analysis it was shown that this unmodified TFO sequence can form a stable triple helix at ∼pH 6 (*T_M_* > 45°C). (35)

**Figure 4.**
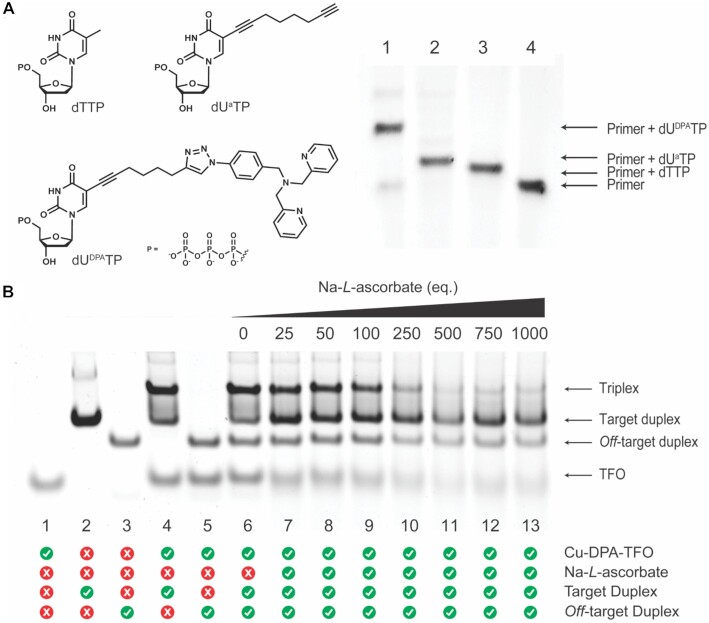
(**A**) 20% denaturing PAGE analysis of SNI-PEX products, showing incorporation of Lane 1: dU^DPA^TP. Lane 2: dU^a^TP. Lane 3: dTTP, and Lane 4: no incorporation. Band densitometry was performed to calculate incorporation efficiency. (**B**) 20% native PAGE showing targeted cleavage by the enzymatically synthesised Cu(II)-DPA-TFO2. Lane 1: TFO (32 nt). Lane 2: Target duplex (57 bp). Lane 3: *Off*-target duplex (40 bp). Lane 4: TFO and target duplex hybridise to form triplex. Lane 5: TFO does not bind with *off*-target duplex. Lane 6–13: Target and *off*-target duplexes incubated with Cu(II)-DPA-TFO2 (25 eq.) and 0–1000 eq. Na-*L*-ascorbate. TFO2 only binds with its target sequence inflicting DNA damage, while *off*-target remains relatively intact. A similar cleavage profile was observed for the SPS TFO1.

A biotinylated template strand complementary to the TFO sequence and a primer, which forms the beginning of the TFO, were required for SNI-PEX. The template and primer were annealed in the presence of a DNA polymerase, and the modified nucleotide was incorporated immediately after the primer (Figure 3C). However, the incorporation of a nucleotide bearing the large DPA modification required significant optimisation. The suitability of various DNA polymerases, including Sequenase V.2.0, Therminator, and Vent (exo-), was investigated. Template, primer and dU^DPA^TP were incubated with each DNA polymerase in their recommended buffer. Sequenase V.2.0 was inefficient at incorporating the dU^DPA^TP and a large concentration of unreacted primer remained after 90 min of incubation. When therminator DNA polymerase was tested, multiple non-templated dU^DPA^TP bases were present (Supplementary Figure S-16) and the major product was the primer with three additional bases. Vent (exo-) DNA polymerase also incorporated multiple non-templated bases but with short incubation times (5 – 20 min) the major products were the primer with the desired dU^DPA^TP incorporated along with the primer with one templated and one non-templated base (Supplementary Figure S-17). Therefore, further optimisation was performed with Vent (exo-) DNA polymerase. To limit the incorporation of non-templated bases short incubation times (≥ 1 min), low concentrations of polymerase, and 1 equivalent of dU^DPA^TP to primer were tested. Unfortunately, no suitable products were obtained. Next, altering the reaction conditions to disfavour polymerase efficiency was considered. By lowering the reaction temperature from 60°C to 37°C a maximum of two dU^DPA^TPs were incorporated and further optimisation at 37°C yielded the desired product—the primer with a single dU^DPA^TP incorporated (Figure 4A). The remainder of the oligonucleotide was then synthesised by adding a high concentration of natural dNTPs (dCTP, dGTP, dTTP and dATP) to the SNI-PEX reaction mixture—essentially saturating the modified base—and extending the primer-dU^DPA^TP strand. This SNI-PEX protocol was then repeated for the synthesis of a methylated TFO; in this instance the natural dCTPs were replaced with 5-methyl-dCTPs (5mC) and thus every cytosine base in the sequence was methylated. Next, the newly synthesised TFOs were isolated from their template and reaction components. Streptavidin coated magnetic particles were employed for this task, but significant optimisation was required. Conditions previously reported (52,70,71) were not suitable due to the high melting temperature of the TFO-template duplex (∼85°C) and the instability of the streptavidin-biotin interaction above 70°C. (70) Therefore, conditions that lower the melting temperature of dsDNA, but do not impact the streptavidin-biotin bonds were required. Since the presence of salts is widely known to affect the melting temperature of DNA, (72–75) various buffers containing NaCl and MgCl_2_, along with concentrations of NaOH, DMSO, and formamide were tested, to no success. Next, the impact of various less commonly used salts was investigated. Here, sodium pyrophosphate was found to be suitable for the task, as it reduced the TFO-template dsDNA melting temperature to ∼61°C, enabling magneto-separation to be performed below the streptavidin-biotin stability threshold. DPA-TFOs were successfully isolated at yields of 57.8% and 61.5%, (Supplementary Table S-4) for TFO2 and 5mC-TFO3, respectively. Finally, the enzymatically synthesised DPA-TFO (TFO2) was analysed and compared to a DPA-functionalised TFO prepared using a combination of SPS methodology and post-synthetic click functionalisation (TFO1, yield 21.1%) (Supplementary Table S-1). Data here revealed that the enzymatic incorporation of a single DPA moiety was successful (∼78%) with a fraction of unreacted primer (∼20%) remaining in solution (Figure 4A, lane 1; Supplementary section S-3). Therefore, SNI-PEX successfully produced the DPA-TFO hybrid together with non-modified TFO (Supplementary Figure S-18; Supplementary Table S-5).

### Thermal melting analysis

Fluorescence thermal melting experiments were performed to determine the stability of the DPA-TFO hybrids. Each TFO was incubated with target duplex in phosphate buffer (pH 6.1) at 37°C for 24 hours. SYBR green I was added, and thermal melting was performed by heating the sample to 95°C while recording ten fluorescence measurements per °C (Supplementary Figure S-19). The TFOs were found to form stable triplexes, with T_M_ values of 45°C − 46.2°C obtained for DPA-TFO1, DPA-TFO2, copper (II)-bound Cu-DPA-TFO1 and Cu-DPA-TFO2 (Table 1). The introduction of 5mC in TFO3 further stabilised the DNA triplex with a T_M_ of 55.3°C recorded in the absence of copper (Δ = +13.8°C), while significantly it enabled triplex formation at neutral pH (T_M_ = 44.9°C).

**Table 1.** Triplex melting temperature for DPA-TFOs produced using SPS (TFO1) and SNI-PEX (TFO2 and 5mC-TFO3)

**Oligo name**	***T* *_M_* (°C)**
SPS DPA-TFO1	45.2
SPS Cu-DPA-TFO1	45.7
PEX DPA-TFO2	45.5
PEX Cu-DPA-TFO2	46.2
DPA-5mC-TFO3 (pH 6.0)	55.3
DPA-5mC-TFO3 (pH 7.0)	44.9

### Targeted oxidative cleavage by DPA-TFO hybrids

**Figure 5.**
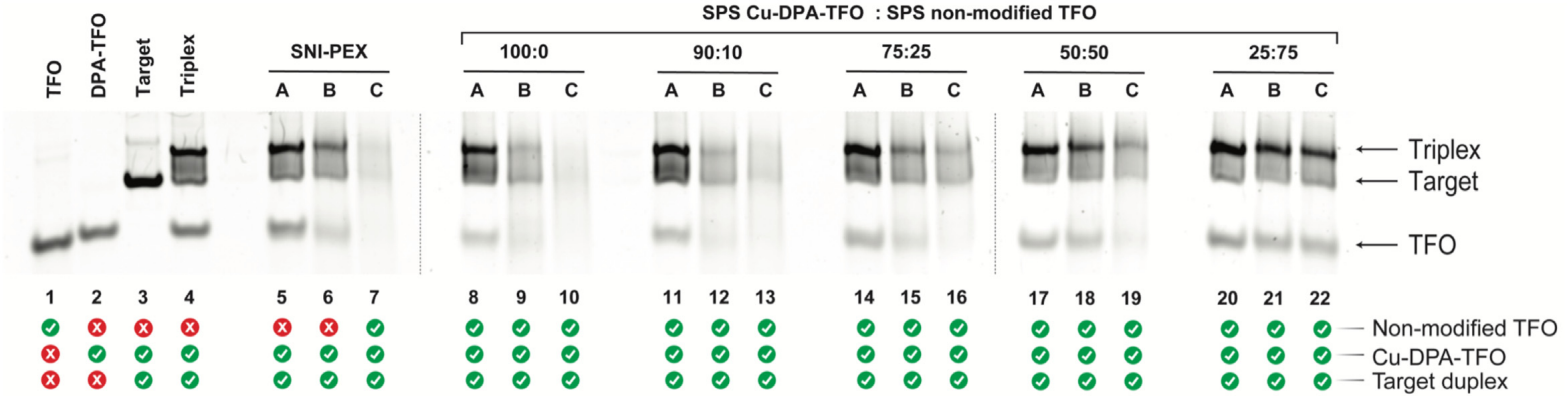
Native PAGE (20%) results of spiking cleavage study. Cu-DPA-TFO1 was spiked with 0, 10, 25, 50 or 75% non-modified TFO (100:0, 90:10, 75:25, 50:50 and 25:75 SPS Cu-DPA-TFO: SPS non-modified TFO, respectively). Lane 1: Non-modified TFO. Lane 2: DPA-TFO1. Lane 3: 57 bp target duplex. Lane 4: Control triplex. Lanes 5–7: SNI-PEX DPA-TFO2. Lanes 8–10: 100% Cu-DPA-TFO1. Lane 11-13: 90% Cu-DPA-TFO1, 10% non-modified TFO. Lanes 14-16: 75% Cu-DPA-TFO1, 25% non-modified TFO. Lanes 17-19: 50% Cu-DPA-TFO1, 50% non-modified TFO. Lanes 20-22: 25% Cu-DPA-TFO1, 75% non-modified TFO. Lanes A, B and C = 50, 500 and 1000 equivalents Na-*L*-ascorbate, respectively. The presence of 10% non-modified TFO has a small impact on DNA cleavage, while significant DNA cleavage inhibition was observed above 25% non-modified TFO.

The ability of the DPA-TFOs to recognise and bind with their genomic target was investigated using polyacrylamide gel electrophoresis (PAGE). Here, a 57 bp region of the GFP gene containing the TFO recognition site and a random *off*-target 40 bp region of the pUC19 plasmid (which lacks the TFO binding sequence) were selected. To determine the sequence specificity of the DPA-TFO hybrid, target and *off*-target duplexes were incubated together with the TFO. This revealed the TFO formed a triplex only with its target sequence, while the *off*-target remained unaltered (Supplementary Figure S-20**)**. Next, the oxidative cleavage of the enzymatically synthesised copper-bound DPA-TFO was determined and compared to its SPS counterpart. Here, the Cu(II)-DPA-TFO hybrids 1 and 2 were incubated with target and *off*-target duplexes in the presence of increasing equivalents of Na-*L*-ascorbate. DNA damage was monitored after 15 hours of continuous incubation at 37°C and visualised by PAGE (SNI-PEX hybrid: Figure 4B; SPS hybrid: Supplementary Figure S-21). The band intensity of the target duplex decreased as the concentration of Na-*L*-ascorbate increased, while the *off*-target duplex remained relatively unchanged (see Supplementary Figure S-22 for band densitometry analysis). It was noted that the target duplex was not entirely depleted at the highest concentration of ascorbate (1000 eq. to TFO) and we hypothesise this is due to the Cu-DPA-TFO damaging the target strand and thereby preventing appropriate recognition by the hybrid (discussed further in ‘Targeted damage of supercoiled plasmid DNA’ and ‘Cleavage resolution experiments’ sections). Significantly, earlier work involving copper(II) phenanthrene AMN-TFO hybrids demonstrated similar activity where DNA cleavage produces an equilibrium between the triplex and duplex conformations due to targeted oxidation at the recognition site. (36) Similar results were obtained for the SPS produced DPA-TFO1 and the SNI-PEX hybrid (TFO2). This experiment was then repeated under identical conditions but the hybrid was replaced with free Cu(II)-DPA complex and an alkyne-modified-TFO. The products were again analysed by PAGE and no significant DNA damage was observed on either the target or *off*-target duplexes (Supplementary Figure S-23). Next, a time and temperature-based study was performed to investigate their impact on targeted DNA cleavage (Supplementary Figure S-24). At 20°C no significant damage was observed after incubation for 24 hours (Supplementary Figure S-24A), whereas at 30°C (Supplementary Figure S-24b) and 40°C (Supplementary Figure S-24c) DNA cleavage was observed after 15 hours of incubation. Finally, DNA cleavage threshold experiments were performed to examine the impact of non-modified TFO in a sample. Here, SPS DPA-TFO1 was spiked, at ratios of 10–75%, with non-modified TFO and the impact on DNA damage was assessed (Figure 5). Data here confirms DPA-TFO2 cleavage matches the activity of the 25% spiked DPA-TFO1, which strongly correlates with earlier identified SNI-PEX incorporation efficiency.

### Mechanistic studies

**Figure 6.**
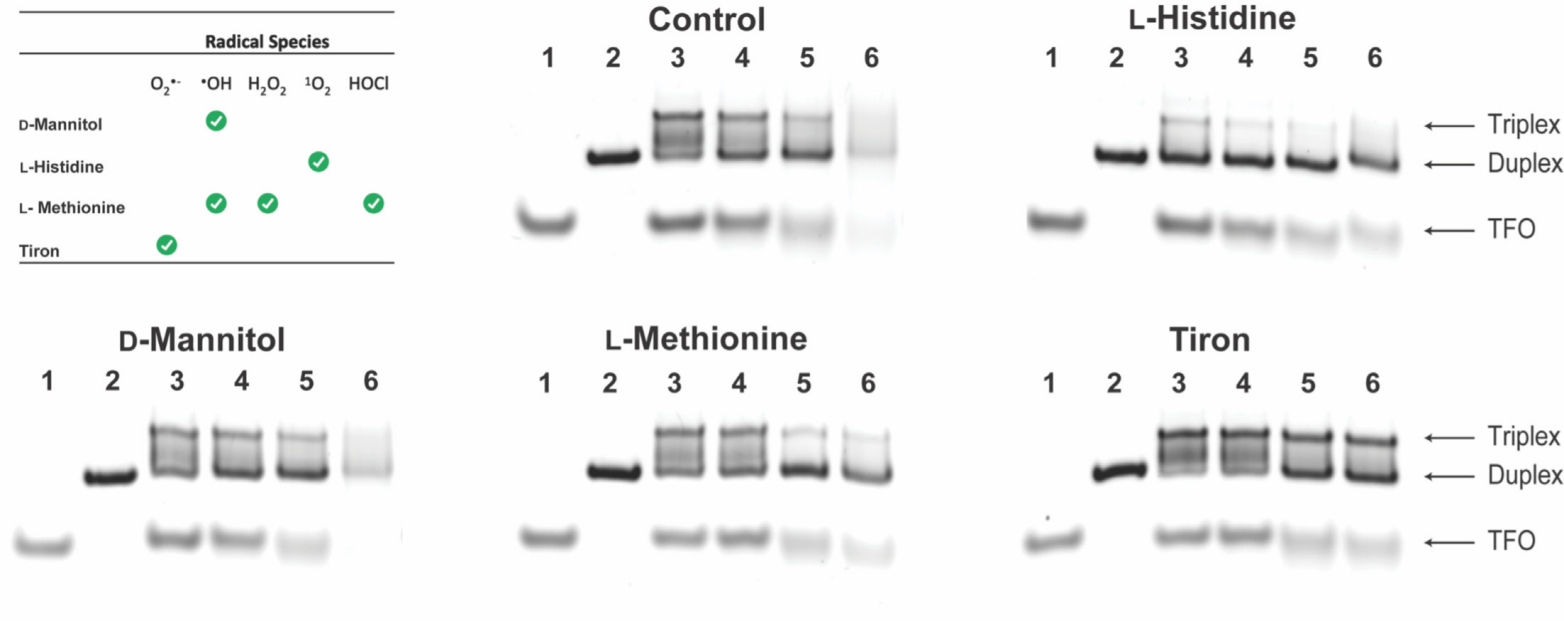
20% native PAGE showing impact ROS have on activity of Cu-DPA-TFO. Lane 1: TFO. Lane 2: Target duplex. Lane 3–6: Cu-DPA-TFO incubated with target duplex and ROS scavenger and 0, 50, 500 or 2000 equivalents of Na-*L*-ascorbate. Control contains no scavenger species.

To determine the radical species involved in the Cu-DPA-TFO cleavage pathway, experiments were performed using reactive oxygen species (ROS) scavengers: L-histidine (singlet oxygen; ^1^O_2_); D-mannitol (hydroxyl radical; ^•^OH); L-methionine (hydroxyl radical; ^•^OH, hypochlorous acid; HOCl, and hydrogen peroxide; H_2_O_2_,); or tiron (superoxide; O_2_^•−^). The hybrid was incubated with the target duplex, Na-*L*-ascorbate, and a ROS scavenger and the products analysed by PAGE (Figure 6). In the presence of tiron no significant DNA cleavage was observed indicating damage occurs primarily through a superoxide mediated pathway, which provides a route to the formation of oxidatively active Cu-O_2_^•−^ intermediates. (21,76) Incubation with L-histidine and L-methionine resulted in low levels of DNA damage inhibition, while D-mannitol had no impact (Figure 6).

### Quantification of DNA damage by qPCR

**Figure 7.**
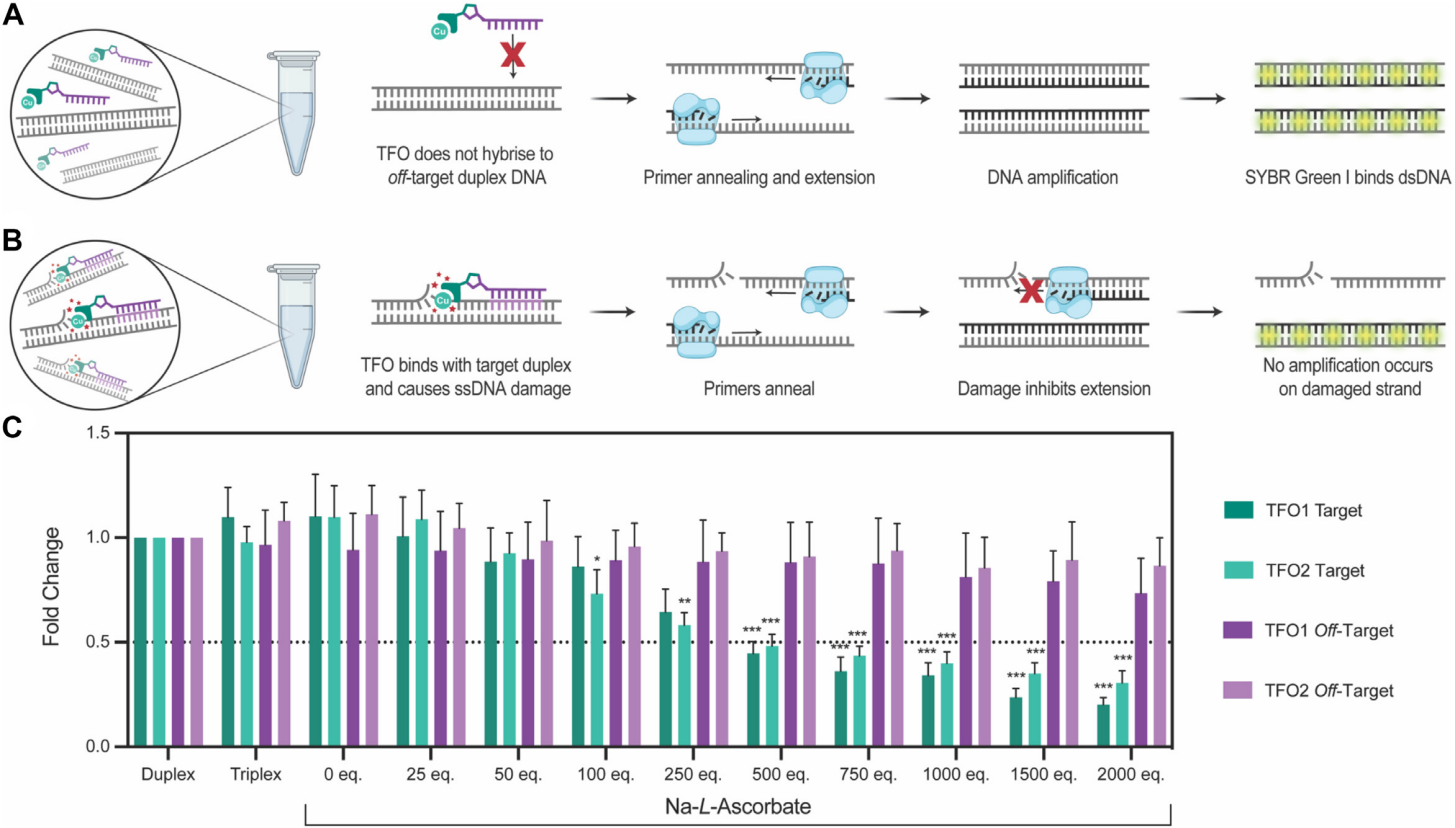
(**A**) The TFO does not hybridise with the *off*-target duplex and consequently the *off*-target was not damaged. Thereby successful cycles of PCR were completed, and SYBR green fluorescence was detected. (**B**) The DPA-TFO hybrid has damaged a single strand of its dsDNA target. Amplification of the damaged strand was inhibited. SYBR green I only binds with intact dsDNA and consequently a lower fluorescent signal was observed. (**C**) Damage inflicted upon the target and *off*-target duplexes during incubation with Cu(II)-DPA-TFO (25 eq.) was analysed by comparing the threshold cycle (C_T_) of experiment (15 h) and reference (0 h) samples (experiment C_T_ – reference C_T_) to give ΔC_T_. ΔC_T_ for each sample was normalised to a non-treated control (ΔΔC_T_) and plotted as a linear value (2^−ΔΔCT^) of fold change in intact DNA. A significant difference is observed between the target and *off*-target for TFO1 and 2, while no significant difference is observed between SPS TFO 1 and SNI-PEX TFO2. * = *P* = < 0.05, significant ** = *P* < 0.01, very significant. *** = *P* < 0.001, extremely significant.

Although PAGE analysis revealed triplex formation and targeted cleavage activity it cannot accurately quantify the DNA damage induced by the DPA-TFO hybrids. Therefore, a real-time quantitative PCR (qPCR) protocol was developed for this task. In this assay a DNA polymerase attempted to amplify a 113 bp fragment of the GFP gene that contained the TFO recognition site. The ability of an *off*-target sequence to undergo PCR amplification was also assessed. Similar to PAGE analysis, target and *off*-target duplexes were incubated with Cu(II)-DPA-TFO1 or the enzymatic Cu(II)-DPA-TFO2 (25 eq.) and increasing concentrations of Na-*L*-ascorbate at 37°C for 15 hours. The reaction was stopped through the addition of EDTA, and samples were diluted to a concentration suitable for PCR. Each sample was then subjected to two qPCR reactions: i.) amplification of the *off*-target duplex (Figure 7A), and ii.) amplification of the target sequence (Figure 7B). The resulting amplification profile for each sequence was analysed and the threshold cycle (C_T_; cycle at which the fluorescent signal is 10 standard deviations above background fluorescence) was noted. In qPCR the C_T_ is directly proportional to the initial concentration of intact dsDNA and therefore the damage induced upon the target and *off*-target duplexes can be compared. It was found that the DPA-TFO hybrids induced significantly more damage on the target sequence when compared to the *off*-target, with 64% versus 13%, and 57% versus 9% of the DNA being damaged in the presence of TFO1 or TFO2, respectively, and 750 equivalents of reductant (Figure 7C), which confirms the sequence selectivity of DPA-TFO hybrids. This result confirmed cleavage sensitivity was not dramatically impacted by the fraction of non-modified TFO present in the sample, and confirmed SNI-PEX as a viable method for the synthesis of AMN-TFO hybrids. The experiment was then repeated using the free Cu-DPA complex and the alkyne-TFO. In this instance no significant damage was observed for either DNA sequence (Supplementary Figure S-25) confirming that attachment of DPA to a TFO not only allows for targeted delivery of the complex but also increases the DNA damaging propensity of the AMN. Since PAGE analysis indicated superoxide was the main ROS responsible for DNA cleavage, a scavenger experiment in the presence of tiron was repeated and analysed by qPCR. Again, no significant damage was observed (Supplementary Figure S-26), which confirms ROS-mediated damage induced by the DPA-TFO hybrid is responsible for preventing DNA amplification.

### Targeted damage of supercoiled plasmid DNA

**Figure 8.**
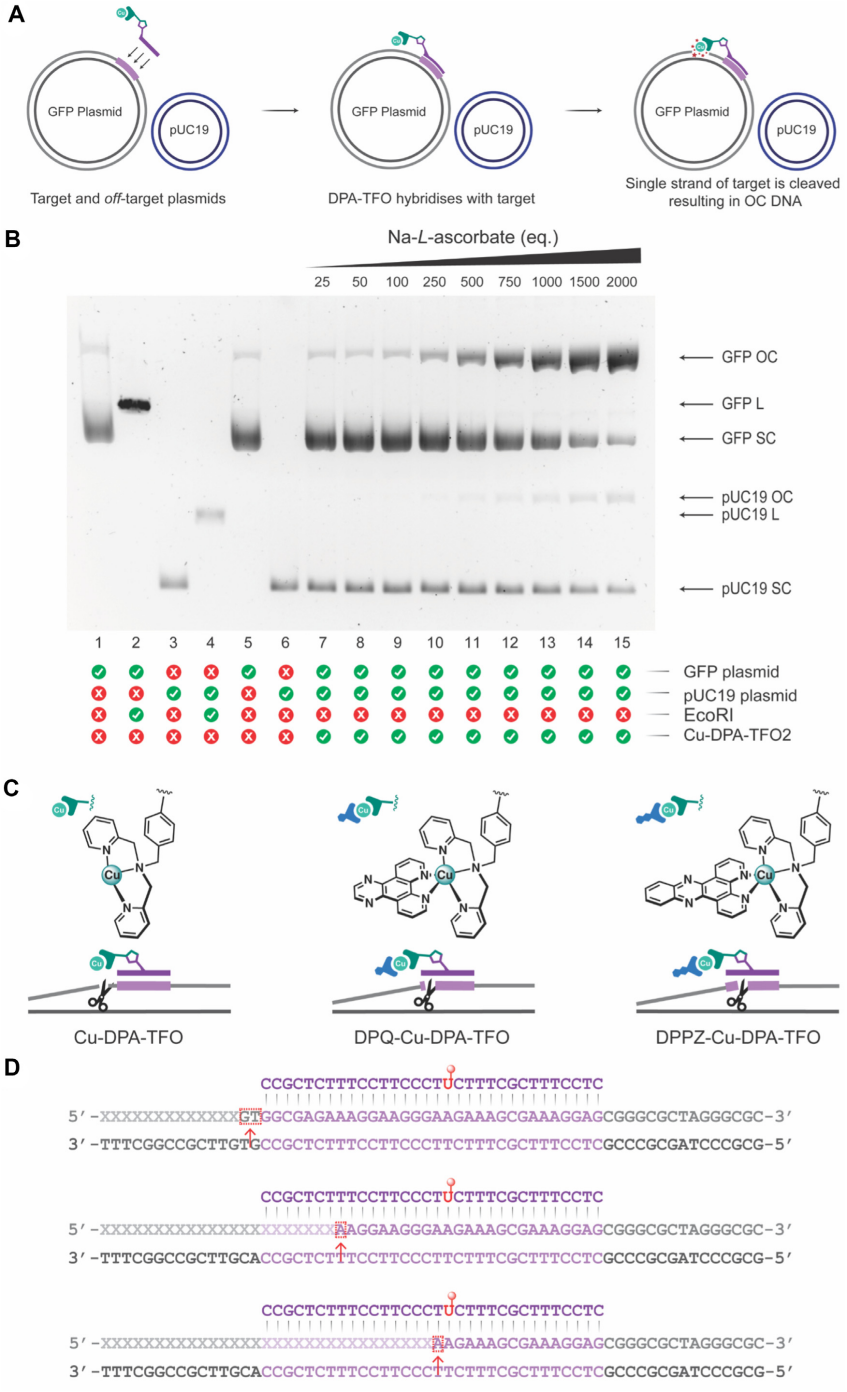
(**A**) DPA-TFO hybrid incubated with target (GFP) and *off*-target (pUC19) plasmid DNA. (**B**) 1% agarose gel electrophoresis. Lane 1: Supercoiled (SC) GFP (6389 bp). Lane 2: GFP linearised by EcoRI. Lane 3: Supercoiled (SC) pUC19 (2686 bp). Lane 4: pUC19 treated with EcoRI. Lane 5: SC GFP control. Lane 6: SC pUC19 control. Lane 7–15: GFP and pUC19 with 25 equivalents of Cu-DPA-TFO2 and 25–2000 equivalents of Na-*L*-ascorbate. The DPA-TFO hybrid can distinguish between the GFP and pUC19 plasmids, it cleaves a single strand of the GFP plasmid, resulting in the formation of open-circular (OC) DNA. (**C**) Molecular structures of Cu-DPA (left), DPQ-Cu-DPA (middle) and DPPZ-Cu-DPA (right), along with cartoon illustration of sequencing cleavage studies involving the enzymatic DPA-TFO hybrid. (**D**) Cu-DPA-TFO2 cuts a single strand of DNA target within 20 bases of the Cu-DPA modification site (top), DPQ-Cu-DPA-TFO2 nicks the target sequence <10 bases from the modification site (middle), and DPPZ-Cu-DPA-TFO2 inflicts a single strand break < 2 bases from the modified dNTP (bottom).

To this point, we have demonstrated how the DPA-TFO hybrids can recognise and damage a specific short DNA sequence. However, DNA within a cell is far more complex than a simple 113 bp duplex as it is folded, twisted, and packaged into tightly wound chromatin (77,78) that may impede effective TFO binding. Therefore, studies involving plasmid DNA were designed to determine if the DPA-TFO hybrids could recognise and damage its target sequence in a more biologically complex environment (Figure 8A). Additionally, supercoiled (SC) plasmid DNA is an ideal substrate to identify oxidative damage as it can delineate single stand damage, resulting in the open-circular (OC) form, and double strand damage, which produces the linear (L) form. (72) Each DPA-TFO hybrid was incubated with a plasmid containing the target GFP gene (pCSanDI-HYG-N44) and an *off*-target pUC19 plasmid, and the resulting products were analysed by agarose gel electrophoresis (TFO1; Supplementary Figure S-27, TFO2; Figure 8B, TFO3; Supplementary Figure S-28). The DPA-TFO hybrid was observed to selectively damage the correct plasmid sequence. It was also noted that the hybrid cleaved a single strand of the GFP plasmid, resulting in relaxation of the SC DNA to its OC form.

Recent investigations into the DNA cleavage affinities of copper(II) DPA complexes with a semi-intercalator, such as Phen, or designer intercalators of DPQ or DPPZ, identified enhanced nuclease activities. (17) Therefore, this study was adapted for use with the enzymatically produced DPA-TFO hybrid, whereby DPQ-Cu(II)-DPA-TFO and DPPZ-Cu(II)-DPA-TFO (25 eq.) were generated and incubated with target (GFP) and *off*-target (pUC19) plasmids, and the resulting DNA cleavage was analysed via agarose electrophoresis (Supplementary Figure S-29). Significantly, the presence of a coordinated intercalator increases DNA binding affinity with the SC target being totally transformed into its OC form. This suggests that optimal placement of intercalating groups is key to optimising the oxidative cleavage process of this class.

### Cleavage resolution experiments

Sanger sequencing was next performed to determine the resolution at which the nuclease-TFO hybrids can cleave their DNA target. Here, the supercoiled GFP plasmid was incubated with Cu-DPA-TFO, DPQ-Cu-DPA-TFO or DPPZ-Cu-DPA-TFO, and the resulting DNA damage was investigated by Sanger sequencing (Figure 8C and D). The sequencing data obtained (Supplementary section S-4) confirmed that the DPA-TFO hybrids nick one strand of the dsDNA target. Interestingly, the pyrimidine strand remains completely intact, while the target purine strand—to which the TFO is hybridised—was damaged. This result differs from earlier reported Fe-EDTA-TFOs developed by Dervan (24) that damaged the pyrimidine strand of the target duplex within the parallel TFO binding domain. Cu-DPA-TFO nicked the purine strand < 20 bases downstream (3′) of the modification site at the triplex-duplex junction— we hypothesise this may occur due to the non-intercalating nature of this copper(II) hybrid interacting with a distal site within the supercoiled substrate—while incubation with DPQ-Cu-DPA-TFO resulted in a single strand break < 10 bases from the DPQ-Cu-DPA modification. Most significantly, the DPPZ-Cu-DPA-TFO nicked the GFP plasmid within 2 bases of the modification site (Figure 8D). This analysis reveals the presence of a designer intercalator—such as DPQ or DPPZ—increases the specificity of DNA cleavage and enables cleavage within the TFO binding region

## CONCLUSION

AMN-TFO hybrids offer an intriguing addition to the field of targeted therapeutics. The TFO is designed to target a genetic sequence of choice—this can be adapted to many desirable purine/pyrimidine rich DNA sequence (36,79,80)—and brings the AMN cutting unit into proximity of the desired cleavage site. The AMN then damages the desired DNA sequence and prevents DNA replication. In this work, a novel azide-functionalised AMN, 4N_3_-benzyl-DPA, was synthesised and tethered to a TFO sequence for targeted DNA cleavage. To overcome the limitations of solid-phase synthesis a protocol for the enzymatic synthesis of AMN-TFO hybrids was developed. This involved the preparation of a DPA-modified nucleotide, incorporation of the dU^DPA^TP into the TFO sequence and isolation of the DPA-TFO. The DPA-modified dUTP was produced through nucleic acid click chemistry and this functionalised dU^DPA^TP was then enzymatically incorporated into the TFO sequence by SNI-PEX. (52) Although the inclusion of modified nucleotides at a specific sequence position has previously been reported, (54,56) the incorporation of a modification of this size had not been achieved. Additionally, to our knowledge, this is the first report of the enzymatic synthesis of an AMN-TFO hybrid. The selection of Vent (exo-) DNA polymerase and its subsequent optimisation enabled a single dU^DPA^TP to be incorporated into the TFO probe with reasonably high efficiency. A key advantage of this approach is that targeted nucleases may now be prepared in a more widely accessible manner, and it can ostensibly be adapted for the synthesis of practically any TFO sequence. This offers flexibility for the type of AMN cleaving unit and its position within the probe, thereby providing notable advantages over the existing state-of-art gene-editing tools. The inclusion of 5mC bases in the DPA-TFO sequence represents one such example of this versatility. The specific incorporation of a large DPA-modification into a heavily methylated sequence demonstrates the adaptability of this technology to the search for future gene-editing systems. An exciting alternative to 5mC is 6-amino-5-nitropyridin-2-one (Z). The triphosphate of this Z base (dZTP) is compatible with enzymatic synthesis (42,81–83) and recently it was reported to completely alleviate the pH dependency of poly-pyrimidine TFOs. (42) Therefore, expansion of the SNI-PEX protocol to include dZTPs and artificial backbones—such as locked nucleic acids or phosphorothioate nucleotides (84)—should unlock the potential of this AMN-TFO technology as a targeted therapy with increased cellular uptake and enhance stability against degradation in biological systems.

The viability of this enzymatic method for the synthesis of functional DPA-TFO hybrids was confirmed through comparisons with a DPA-TFO prepared by SPS with post-synthetic click functionalisation. Thermal melting, electrophoresis and qPCR were performed to evaluate the activity of the DPA-TFOs. No significant difference in thermal stability was observed between the SPS and SNI-PEX TFOs, and both could maintain triplex formation above 45°C. PAGE cleavage analysis revealed that the DPA-TFOs were capable of distinguishing target and *off*-target DNA sequences in both short duplexes and a more complex plasmid environment. However, upon close analysis it was noted that the cleavage efficiency of the SNI-PEX DPA-TFO2 was lower than that of its SPS counterpart. Spiking experiments revealed that this was due to the small concentration of non-modified TFO present in the enzymatic sample. However, qPCR analysis confirmed that there was no significant difference in amplification inhibition between the SPS and SNI-PEX DPA-TFOs, confirming the enzymatic protocol as a viable method for the synthesis of AMN-TFO hybrids. Finally, sequencing studies were performed to investigate the specificity at which the DPA-TFO cleaves its target. The DPA-TFOs demonstrated a high level of target discrimination, and in the presence of a designer intercalator cleavage of the purine strand within two bases of the modification site was achieved. This contrasts with previous studies of single-strand damage inflicted by internally modified Fe-EDTA-TFOs, (24) which predominantly cleave the pyrimidine strand. This indicates that by altering the hybridised AMN cutting unit, DNA cleavage can be directed to either strand of the genetic target. The end-point of this technology is to achieve effective gene-knockout in cellular systems, and the technology reported herein is a fundamental step towards achieving this objective. The enzymatic method and accompanying *in vitro* analyses are essential steps in the pursuit of this long-term goal, with future work involving the expansion of this technology to the synthesis of TFOs bearing a variety of AMNs and artificial bases/backbones. The delivery and toxicity of these AMN-TFO hybrids will also be important factors to consider, but as the field of nucleic acid delivery agents expands, improved opportunities for *in vivo* success for these hybrid systems are likely to arise.

In summary, the enzymatic methodology reported herein was designed to enable the incorporation of a variety of AMNs and therefore will be applicable for the expansion of this AMN-TFO technology. The ability of the DPA-TFOs to specifically modify one strand of a gene of interest and the accessibility of SNI-PEX for the synthesis of highly modified chemical nuclease-TFO hybrids will solidify AMN-TFOs at the forefront of targeted metallodrug design, and we predict that this technology will be invaluable in the pursuit of personalised medicine.

## Supplementary Material

gkac438_Supplemental_File
