# Supplementary material for: Enzymatic Synthesis of Chemical Nuclease Triplex-Forming Oligonucleotides with Gene-Silencing Applications
Source: Nucleic Acids Res. 2022 May 30;50(10):5467–81. doi: 10.1093/nar/gkac438 (PMC9177962; doi:10.1093/nar/gkac438)
Supplement: gkac438_Supplemental_File [file gkac438_supplemental_file.pdf]

Supporting Information accompanying the manuscript

Enzymatic Synthesis of Chemical Nuclease Triplex-Forming Oligonucleotides with Gene-Silencing Applications

Bríonna McGorman,[1] Nicolò Zuin Fantoni,[2] Sinéad O'Carroll,[1] Anna Ziemele,[1] Afaf H. El-Sagheer,[2,3] Tom Brown[2] and Andrew Kellett*[1,4]

List of Content:

S - 1: Synthesis of N-4-azidobenzyl-N-di-(2-picolyl)amine ($4N_3$-Benzyl-DPA)

S - 2: Solid-phase synthesis of DPA-TFO

S - 3: Enzymatic synthesis of DPA-TFO

S - 4: Analysis of sequence specific cleavage by DPA-TFO

S - 1: Synthesis of N-4-azidobenzyl-N-di-(2-picolyl)amine (4N_3-Benzyl-DPA)

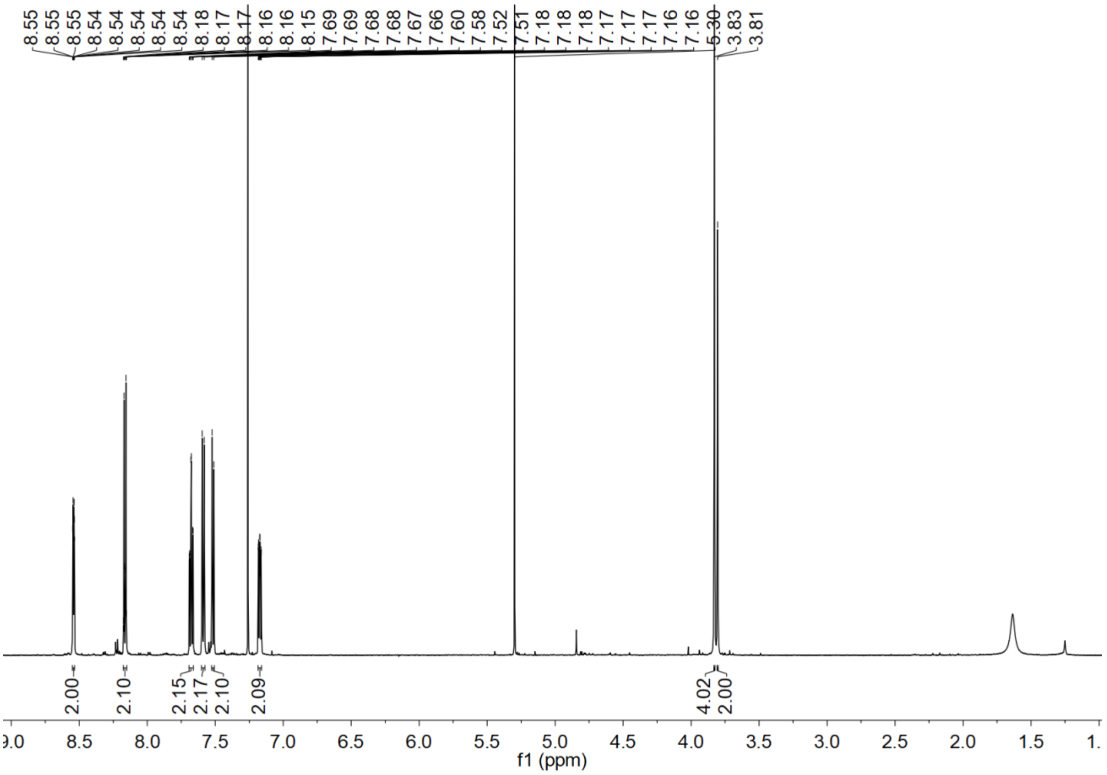

Figure S - 1. ^{1}H-NMR spectrum of N-4-nitrobenzyl-N-di-(2-picolyl)amine (1).

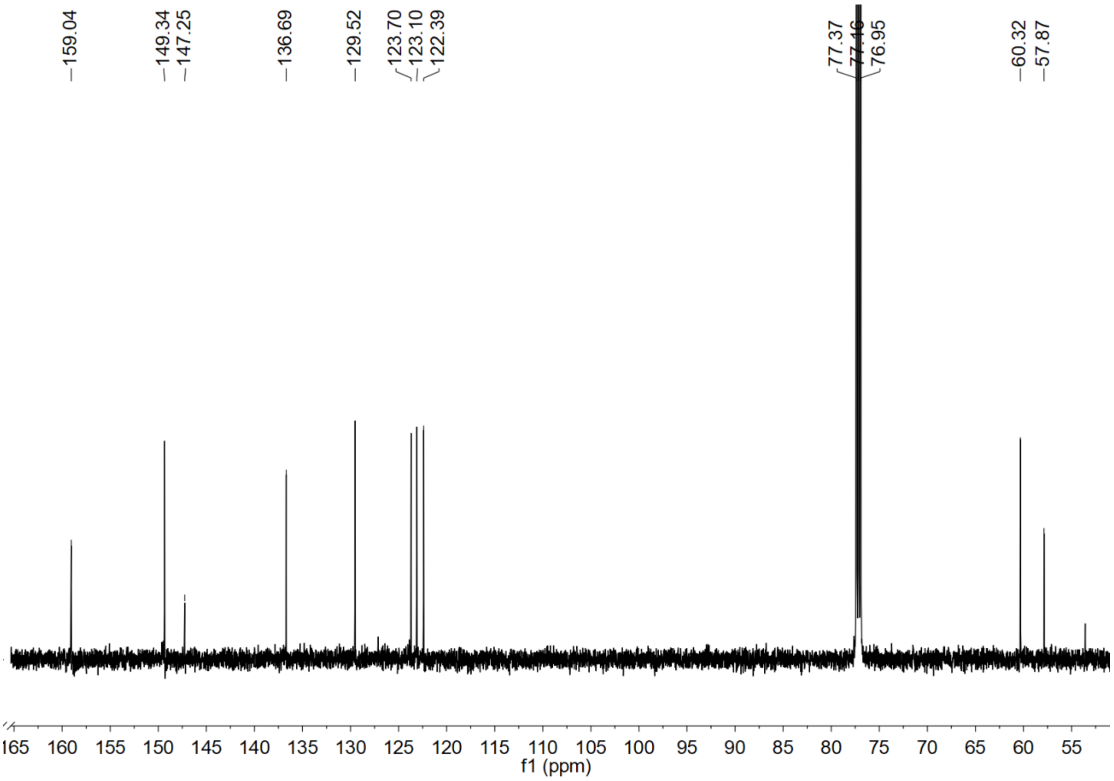

Figure S - 2. ^{13}C-NMR spectrum of *N*-4-nitrobenzyl-*N*-*di*-(2-picolyl)amine (1).

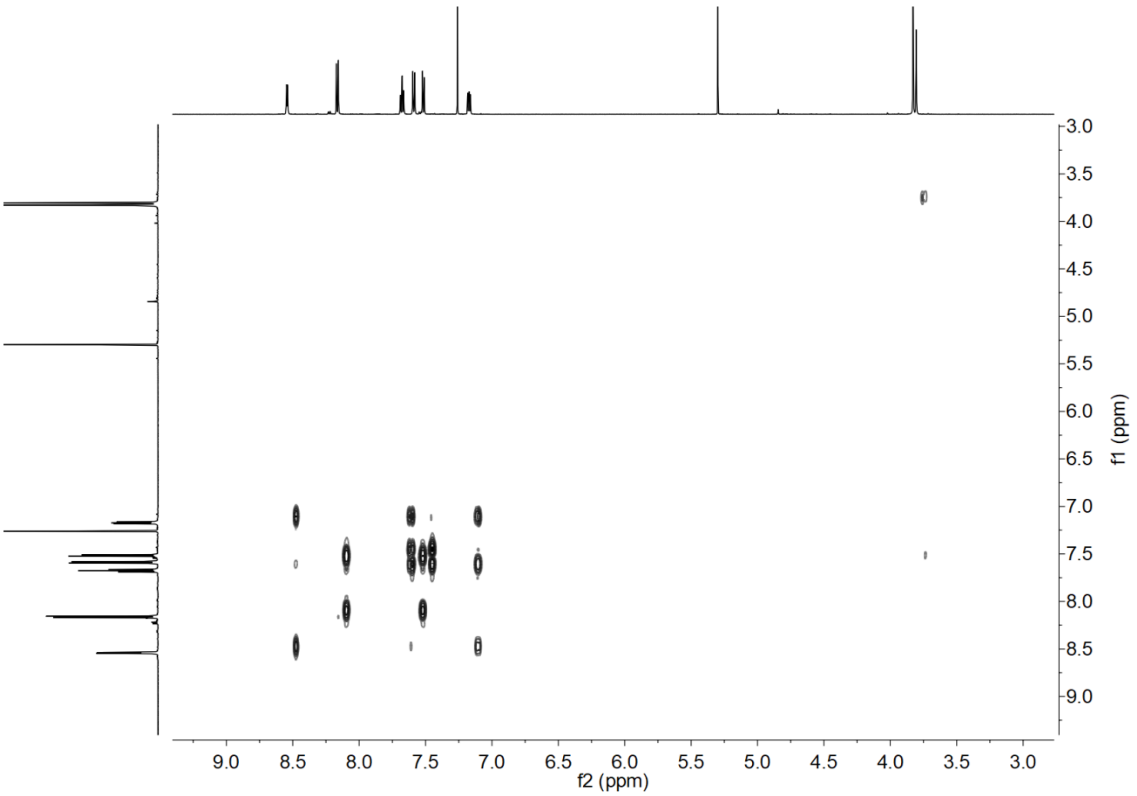

Figure S - 3. ^{1}H-^{1}H COSY spectrum of N-4-nitrobenzyl-N-di-(2-picolyl)amine (1).

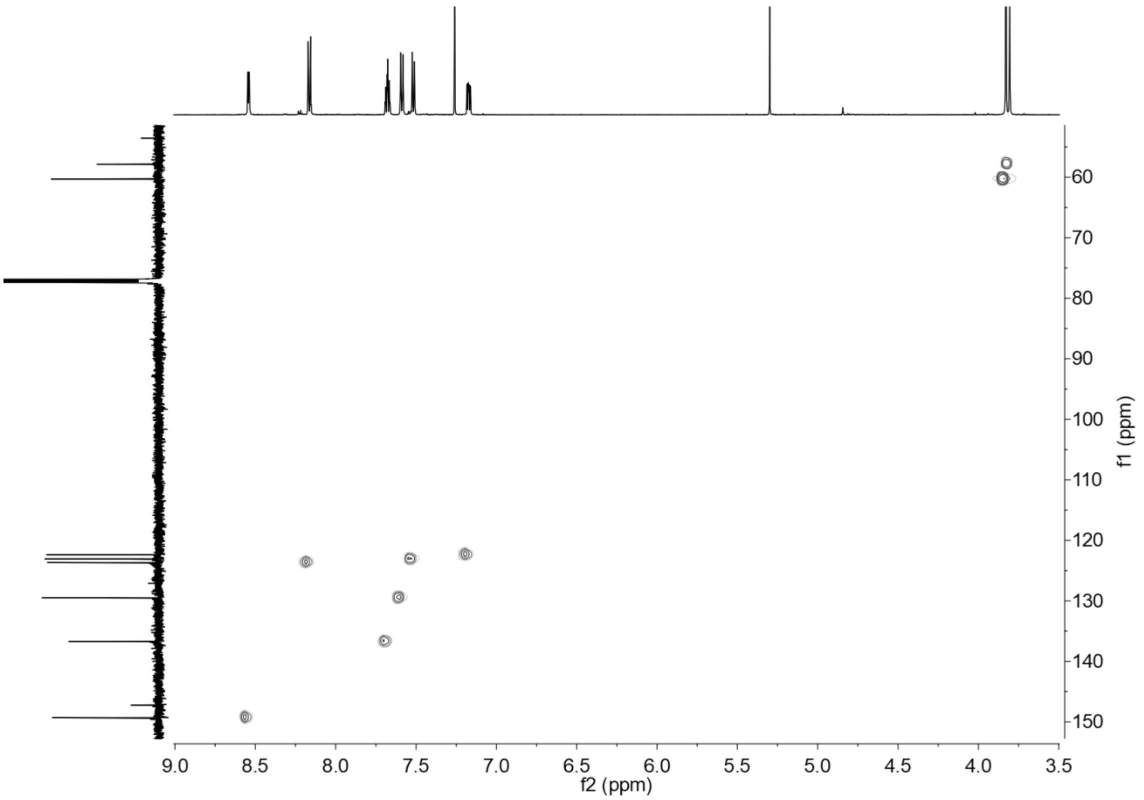

Figure S - 4. ^{1}H-13C HSQC spectrum of N-4-nitrobenzyl-N-di-(2-picolyl)amine (1).

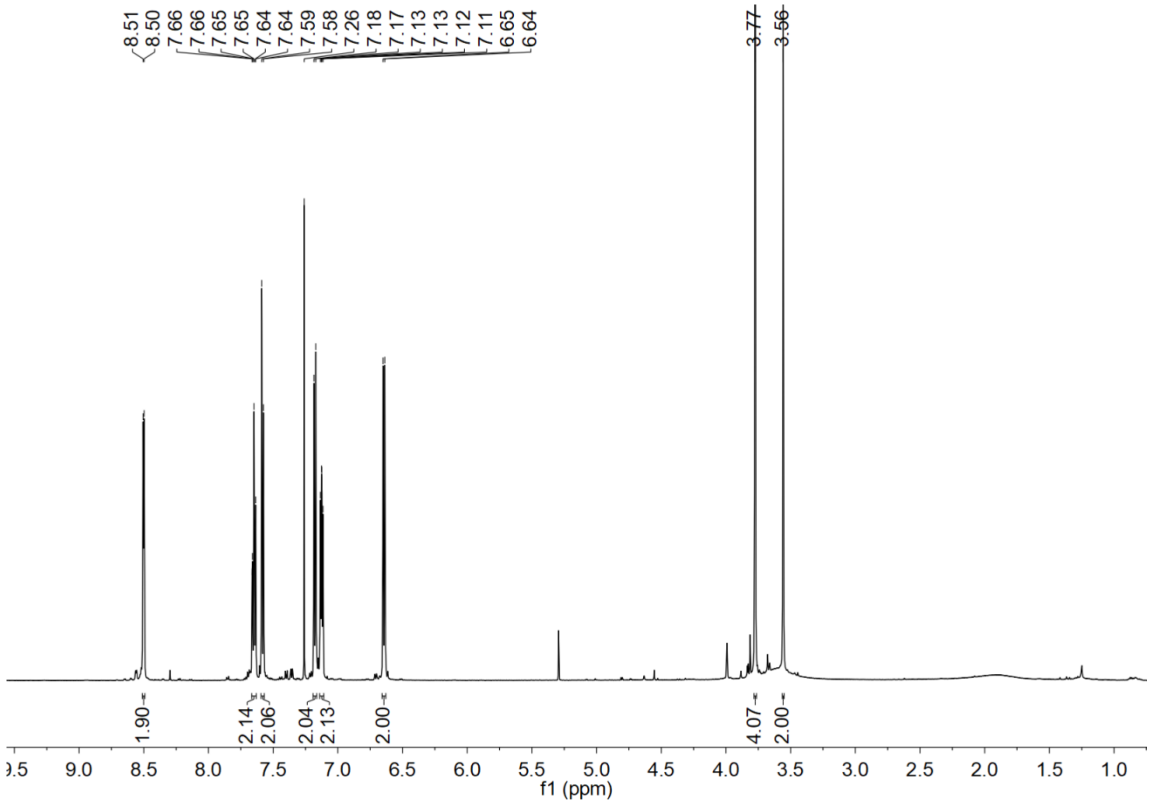

Figure S - 5. ^{1}H-NMR spectrum of N-4-amminobenzyl-N-di-(2-picolyl)amine (2).

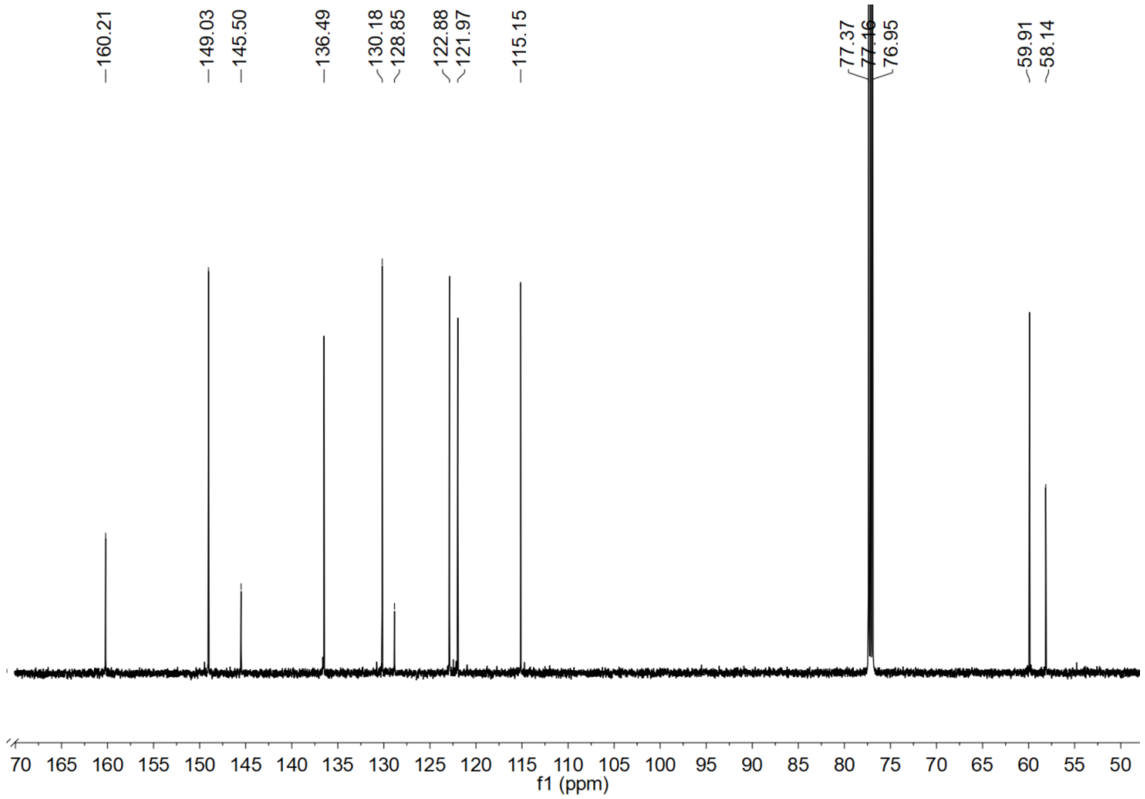

Figure S - 6. ^{13}C-NMR spectrum of N-4-amminobenzyl-N-di-(2-picolyl)amine (2).

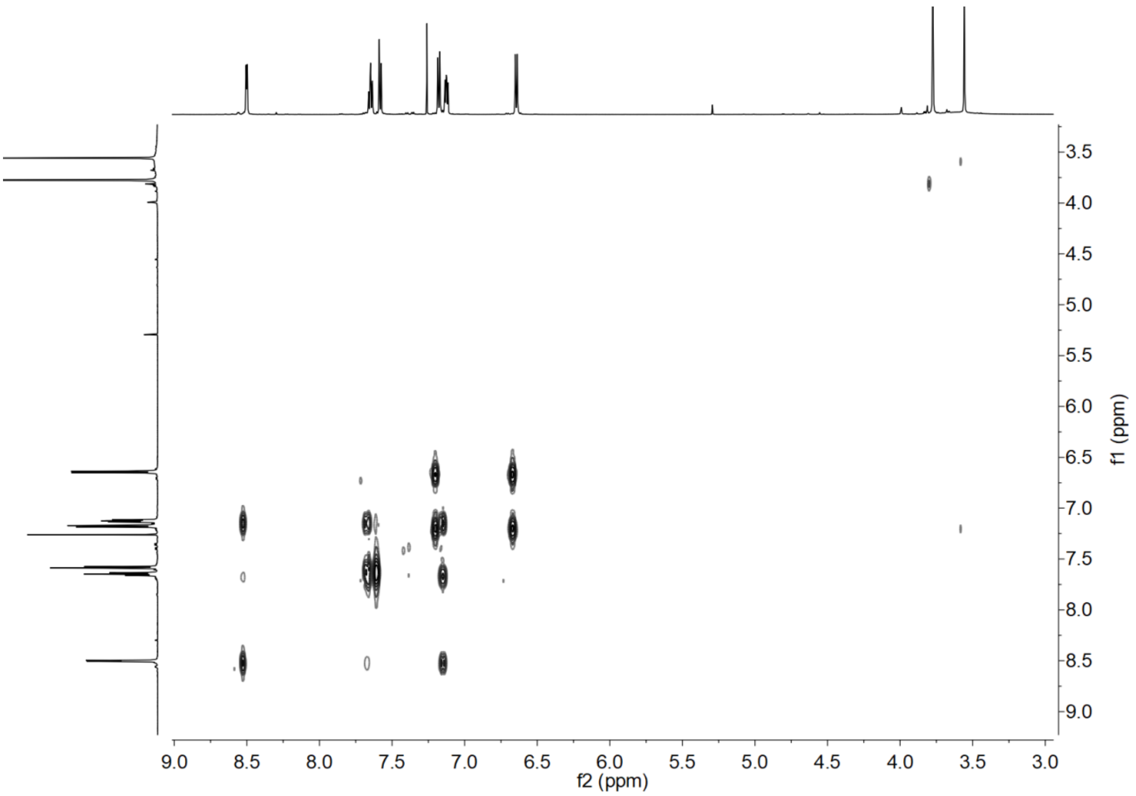

Figure S - 7. ^{1}H-^{1}H COSY spectrum of *N*-4-amminobenzyl-*N*-*di*-(2-picolyl)amine (2).

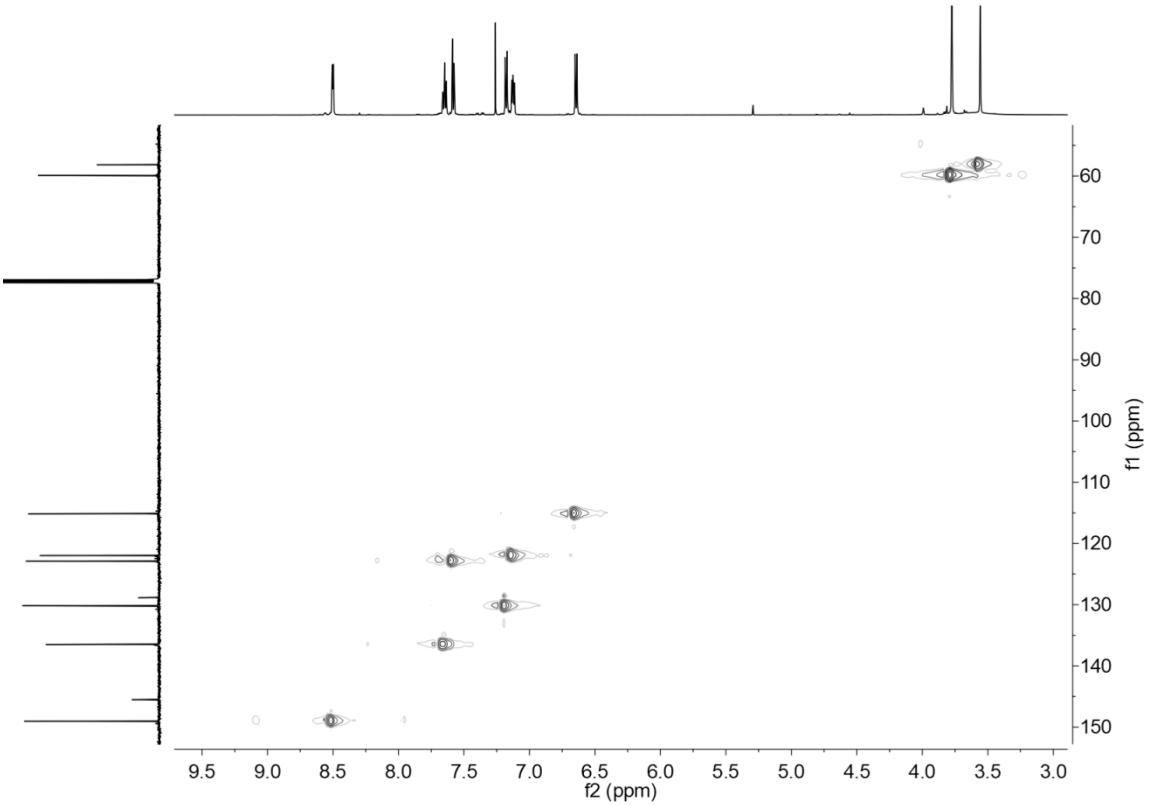

Figure S - 8. ^{1}H-^{13}C HSQC spectrum of N-4-amminobenzyl-N-di-(2-picolyl)amine (2).

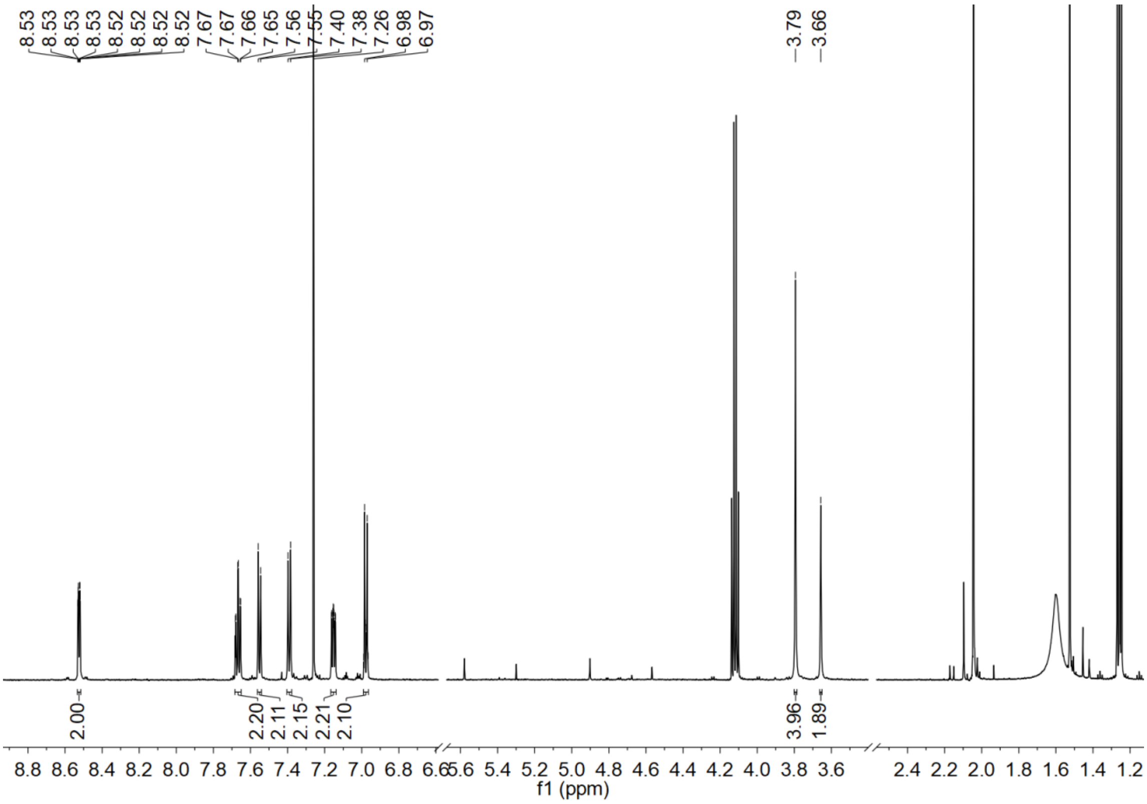

Figure S - 9. ^{1}H-NMR spectrum of *N*-4-azidobenzyl-*N*-*di*-(2-picolyl)amine (3).

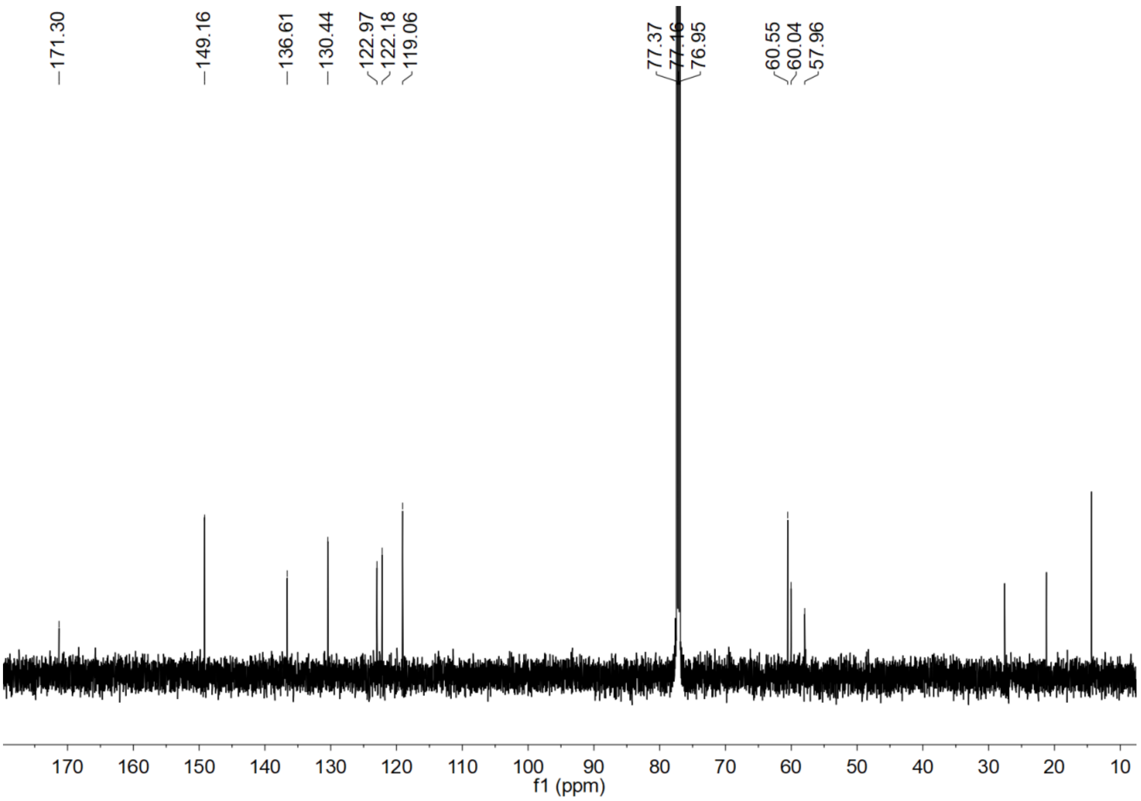

Figure S - 10. ^{13}C-NMR spectrum of *N*-4-azidobenzyl-*N*-*di*-(2-picolyl)amine (3).

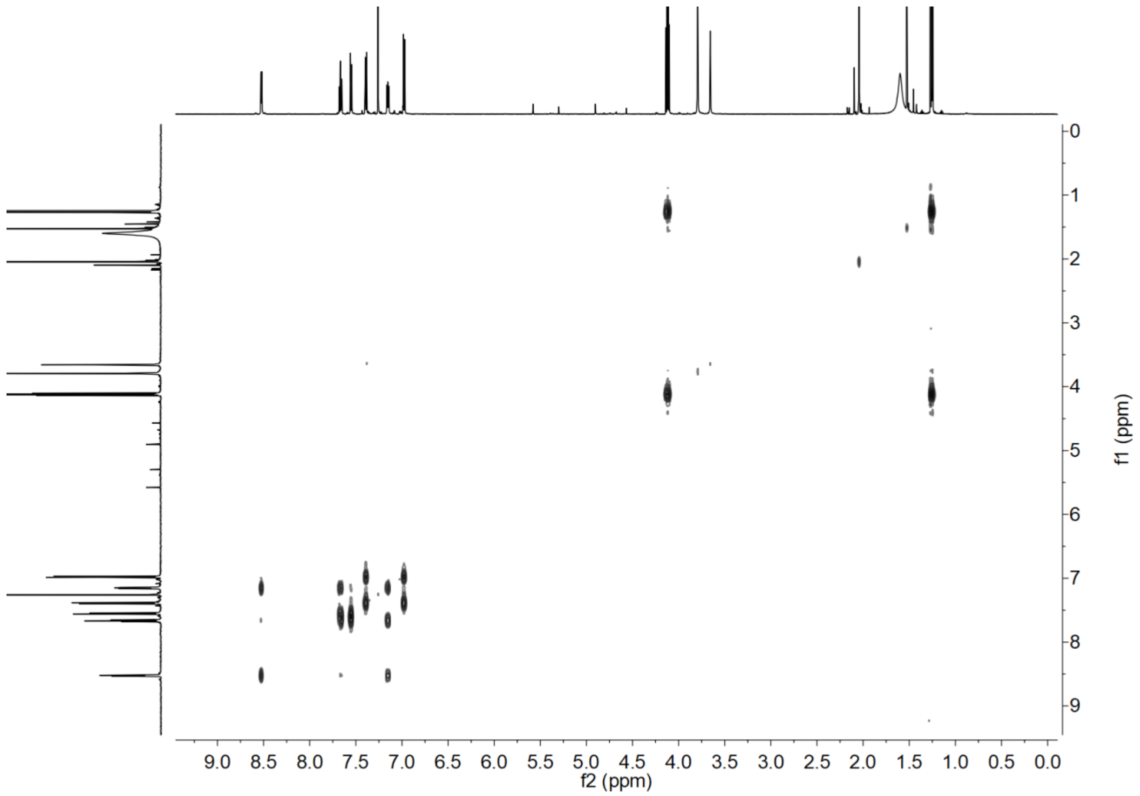

Figure S - 11. ^{1}H-^{1}H COSY spectrum of N-4-azidobenzyl-N-di-(2-picolyl)amine (3).

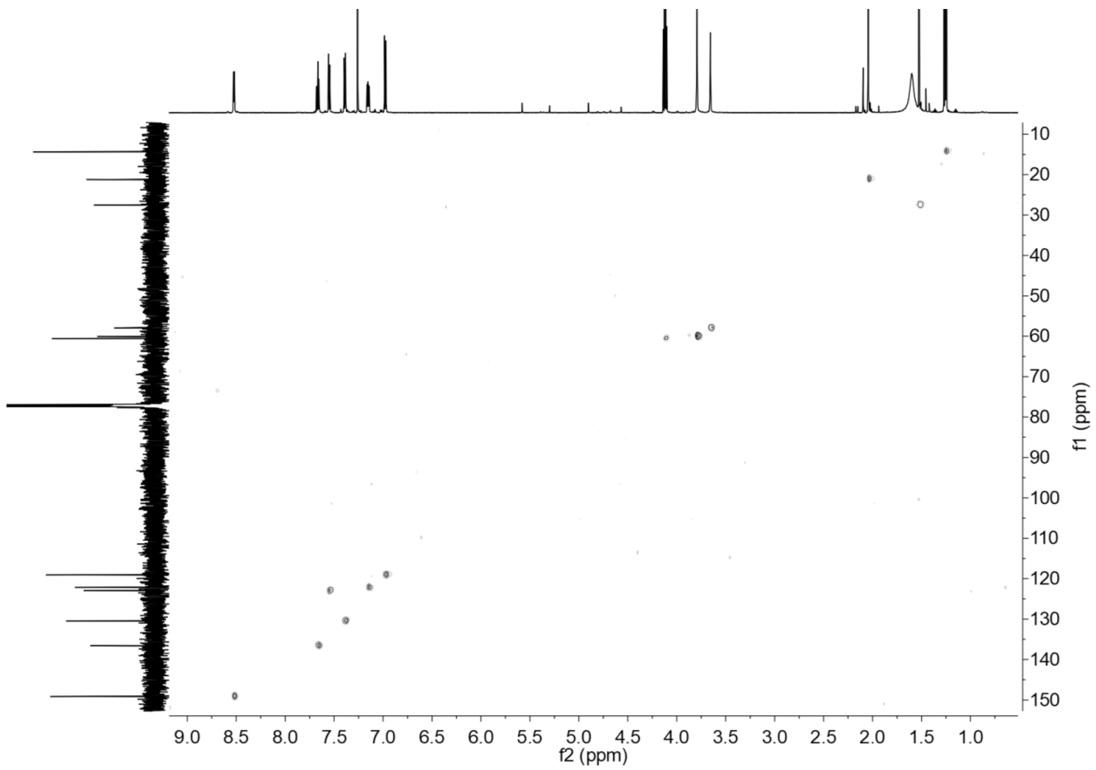

Figure S - 12. ^{1}H-^{13}C HSQC spectrum of N-4-azidobenzyl-N-di-(2-picolyl)amine (3).

S - 2: Solid-phase synthesis of DPA-TFO

S - 2.1 'Click' reaction yields

Table S - 1. Experimental yields of DPA-TFO1 after 'click' reaction, desalting and HPLC purification; int-dU[a] = internal-octadiynyl-deoxyuridine; $4N_3$-DPA = *N*-4-azidobenzyl-*N*-*di*-(2-picolyl)amine.

Oligo	Sequence	Modification	Yield (%)
TFO1	5′ – CGC TCT TTC CTT CCC UTC TTT CGC TTT CCT C – 3′	int-dU[a]-$4N_3$-DPA	21.1

S - 2.2 Mass spectrometry data of SPS TFOs

Table S - 2. Oligonucleotide sequences, modifications and dC content. Calculated and found m/z; (a) = + 63 m/z caused by the ligand scavenging Cu(II) from the UPLC column in the analysis. int-dU[a] = internal-octadiynyl-deoxyuridine; $4N_3$-DPA = *N*-4-azidobenzyl-*N*-*di*-(2-picolyl)amine.

Oligo name	Sequence	Modification	Calcd. Mass	Found Mass
Alkyne-TFO	5′ – CGC TCT TTC CTT CCC UTC TTT CGC TTT CCT C – 3′	int-dU[a]	9298	9297.7
DPA-TFO1	5′ – CGC TCT TTC CTT CCC UTC TTT CGC TTT CCT C – 3′	int-dU[a]-$4N_3$-DPA	9628.4	9690.8[a]

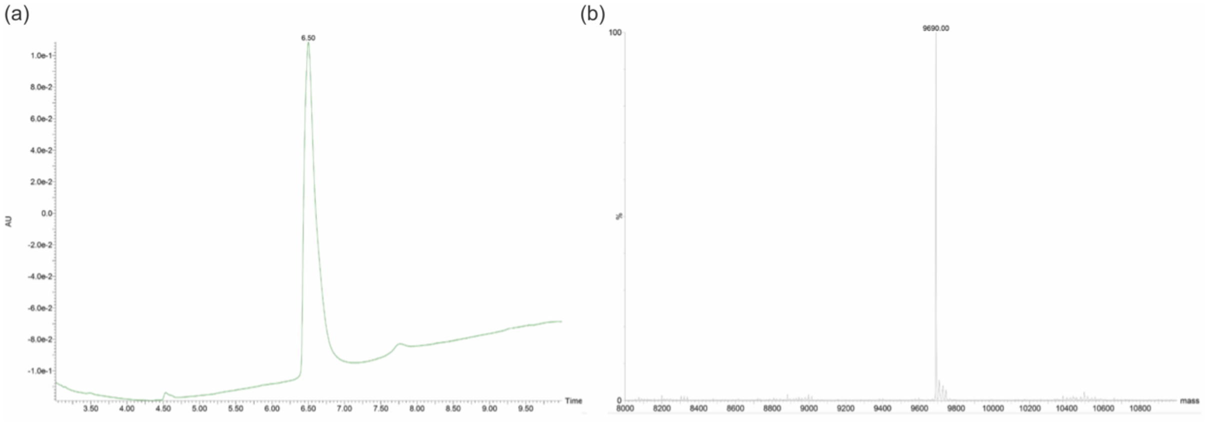

Figure S - 13. (a) UPLC-MS chromatograms of pure DPA-TFO. The SPS DPA-TFO was produced in yields according to **Table S - 1** and mass was confirmed according to **Table S - 2** UPLC x-axis = time (min) and y-axis = UV absorbance at 260nm. (b) Deconvoluted (MaxEnt) mass spectrum of SPS DPA-TFO1.

S - 3: Enzymatic synthesis of DPA-TFO

S - 3.1 Mass spectrometry data of the DPA-dU^{DPA}TP

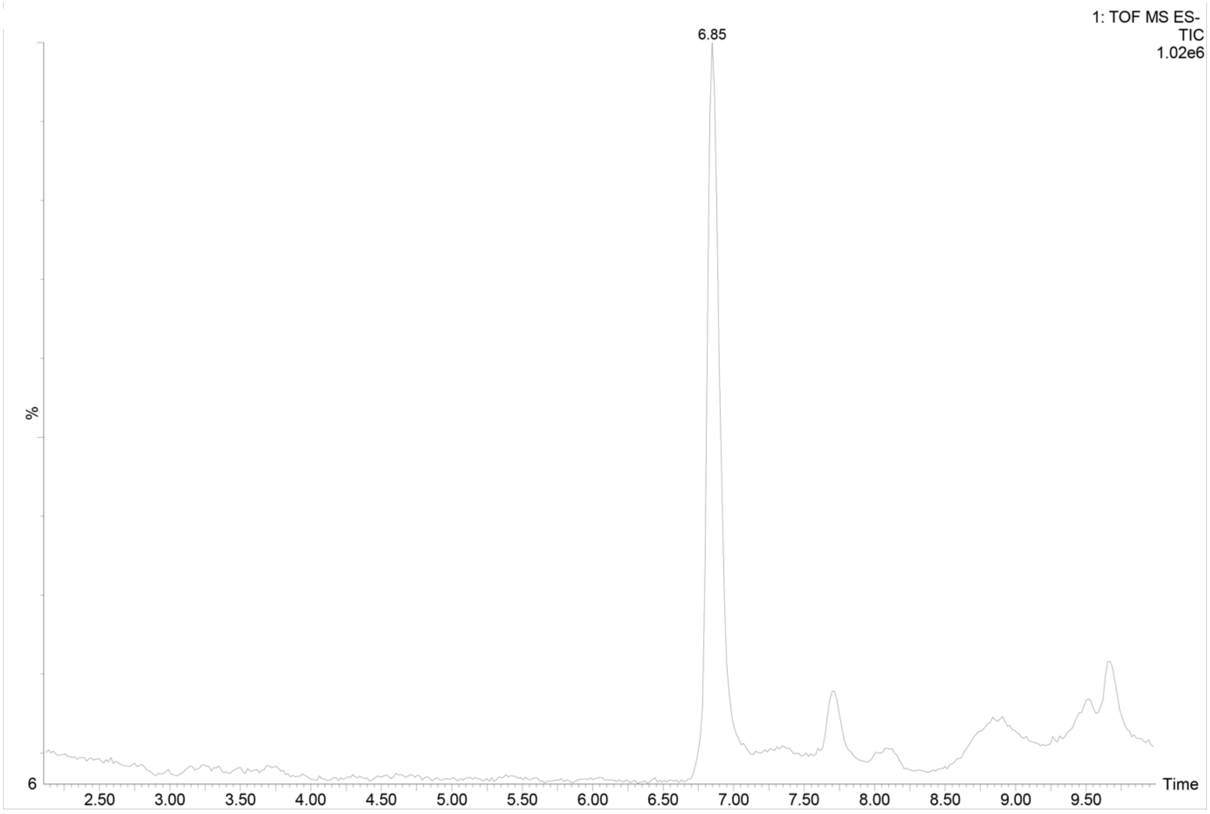

Figure S - 14. UPLC-MS chromatograms of dU^{DPA}TP.

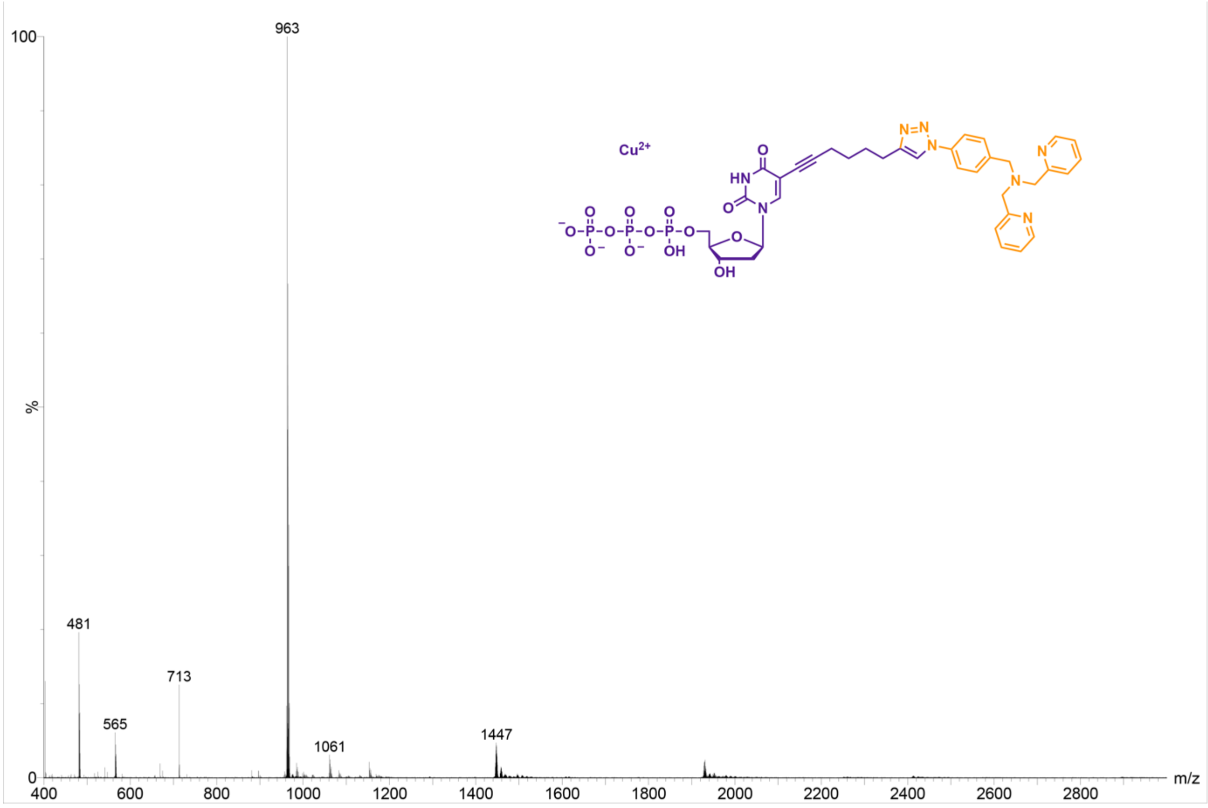

Figure S - 15. ESI-mass spectrum of DPA-dU^{DPA}TP [M+Cu^{2+}]$^{-}$.

S - 3.2 Enzymatic synthesis of DPA-TFO

Table S - 3. Sequence of SNI-PEX primers, template and TFOs. `M` = 5-methyl-cytosine. `Bio` = biotin.

	Length	Sequence (5'-3')
Primer	17 nt	CCGCTCTTTCCTTCCCT
Me-C Primer	17 nt	MMGMTMTTTMMTTMMMT
Template	32 nt	Bio-GAGGAAAGCGAAAGAAGGGAAGGAAAGAGCGG
TFO2	32 nt	CCGCTCTTTCCTTCCCT**U**CTTTCGCTTTCCTC
5mC-TFO3	32 nt	MMGMTMTTTMMTTMMMT**U**MTTTMGMTTTMMTM

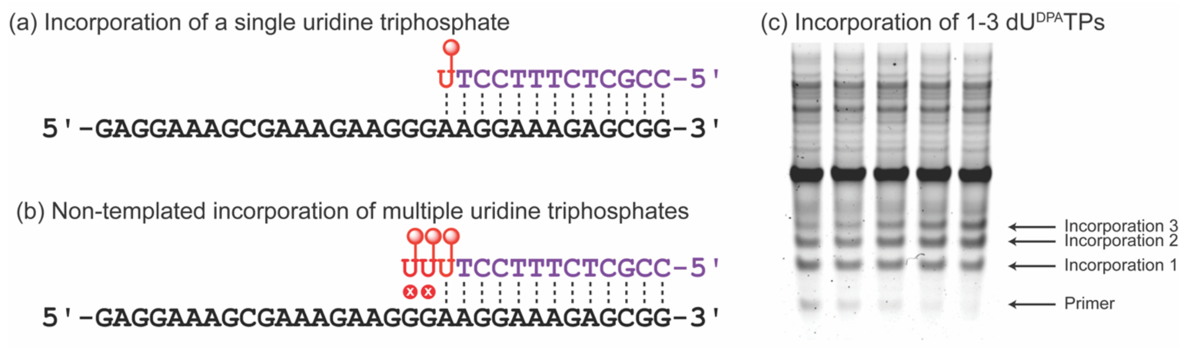

Figure S - 16. (a) Incorporation of a single modified nucleotide at the desired position in the TFO sequence. (b) An additional two uridine nucleotide triphosphates are incorporated. These are non-templated as they will not form Watson-Crick bonds with the guanine bases on the complementary strand. (c) 20% denaturing PAGE analysis of incorporation by therminator DNA polymerase. There is one templated (incorporation 1) and two non-templated (incorporation 2 and 3) incorporations of dU^{DPA}TP.

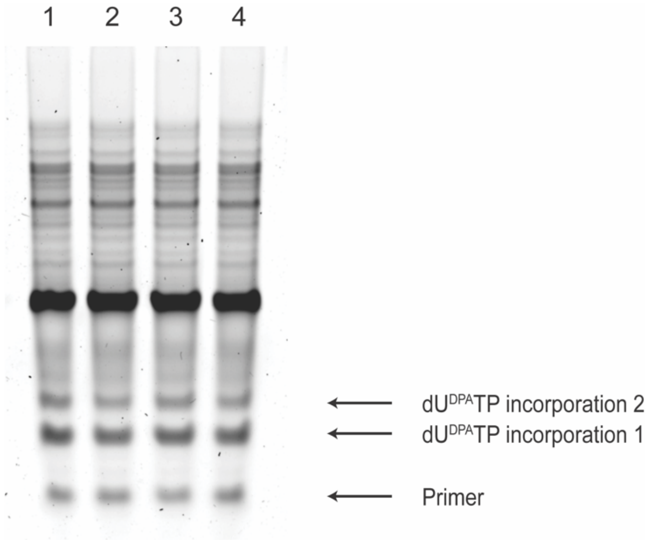

Figure S - 17. 20% denaturing PAGE showing the incorporation of dU^{DPA}TP by vent (exo-) DNA polymerase. Lane 1-4: Incubation time 5, 10, 15 and 20 min, respectively. A maximum of two dU^{DPA}TPs (the first templated and the second non-templated) are incorporated.

Table S - 4. Yield for enzymatically synthesised DPA-TFO hybrids. M = 5-methyl-cytosine. Yield was calculated by comparing concentration of primer/template added to SNI-PEX reaction versus concentration of DPA-TFO determined by UV absorbance.

Oligo name	Sequence	Modification	Yield (%)
TFO2	5′ – CCG CTC TTT CCT TCC CT**U** CTT TCG CTT TCC TC – 3′	$dU^{DPA}TP$	57.8%
5mC-TFO3	5′ – MMG MTM TTT MMT TMM MT**U** MTT TMG MTT TMM TM – 3′	$dU^{DPA}TP$	61.5%

S - 3.3 Mass Spectrometry analysis of SNI-PEX DPA-TFO2

Table S - 5. Oligonucleotide sequence and modifications. Calculated and found m/z; (a) = + 63 m/z caused by the ligand scavenging Cu(II) from the UPLC column in the analysis; (b) = non-modified TFO (i.e. fully canonical sequence); M = 5-methyl-cytosine.

Oligo name	Sequence	Modification	Calcd. Mass	Found Mass
TFO2	5′ – CCG CTC TTT CCT TCC CT**U** CTT TCG CTT TCC TC – 3′	dU^{DPA}	9917.5[a]	9980[a]
TFO2	5′ – CCG CTC TTT CCT TCC CT**T** CTT TCG CTT TCC TC – 3′	dT	9497.1[b]	9497[b]
5mC-TFO3	5′ – MMG MTM TTT MMT TMM MT**U** MTT TMG MTT TMM TM – 3′	dU^{DPA}	10127.5[a]	10191[a]
5mC-TFO3	5′ – MMG MTM TTT MMT TMM MT**T** MTT TMG MTT TMM TM – 3′	dT	9707[b]	9708[b]

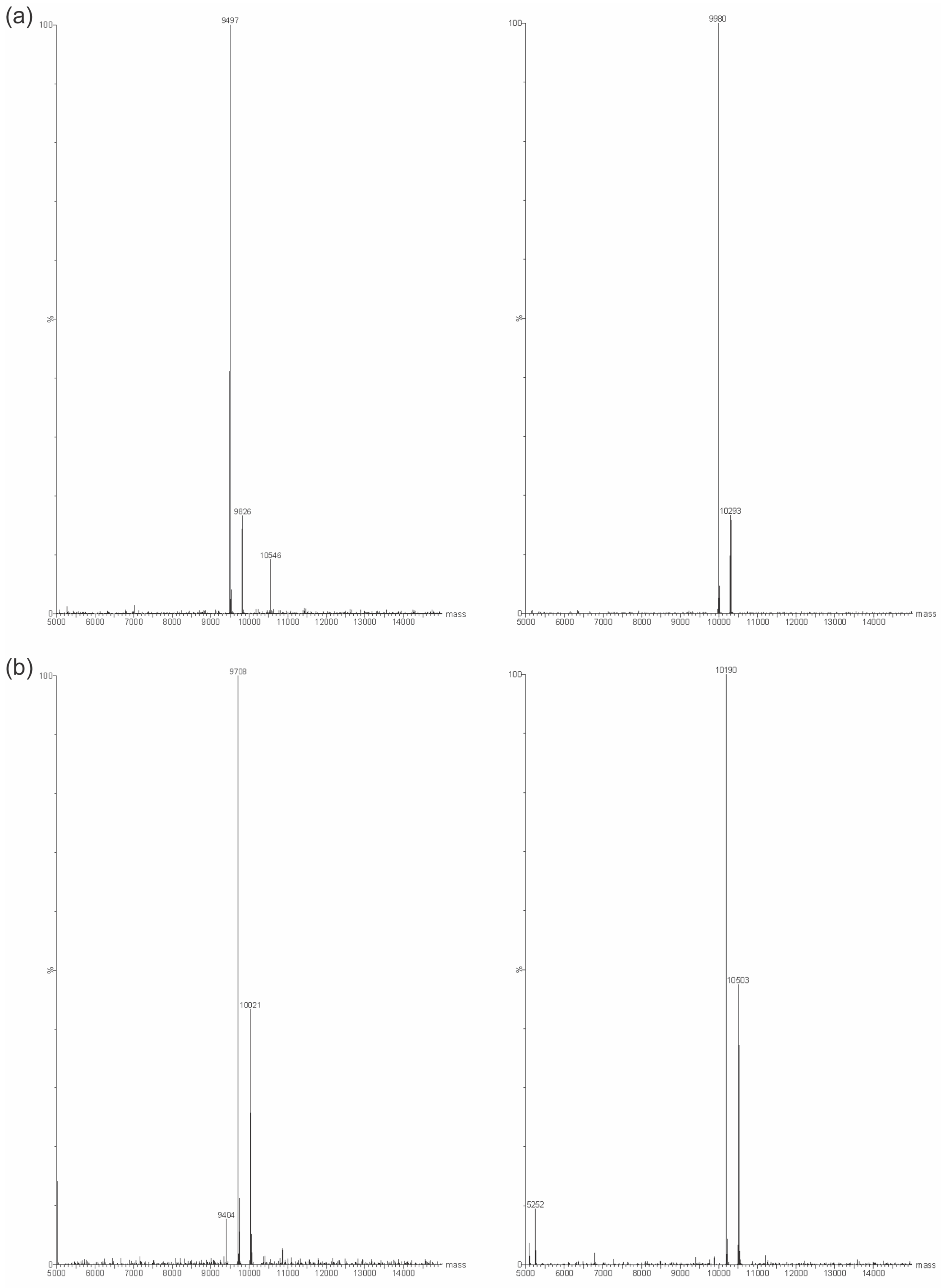

Figure S - 18. (a) ESI-mass spectrum of enzymatically produced DPA-TFO2. Left: Non-modified TFO. Right: DPA-TFO2 [M + Cu^{2+}], (b) ESI-mass spectrum of enzymatically produced 5mC-DPA-TFO3 Left: Non-modified 5mC-TFO. Right: DPA-TFO3 [M + Cu^{2+}].

S - 4: Analysis of sequence specific cleavage by DPA-TFO

S - 4.1 DNA targets for DPA-TFO analysis

Table S - 6. TFO target and *off*-target duplexes.

	Length	Sequence (5'-3')
Target duplex	57 bp	AAGCCGGCGAACGTGGCGAGAAAGGAAGGGAAGAAAGCGAAAGGAGCG<br>GGCGCTAGG
Target duplex	113 bp	AAAGGGAGCCCCCGATTTAGAGCTTGACGGGGAAAGCCGGCGAACGTG<br>GCGAGAAAGGAAGGGAAGAAAGCGAAAGGAGCGGGCGCTAGGGCGCTG<br>GCAAGTGTAGCGGTCAC
Off-target duplex	40 bp	TGACTCCCCGTCGTGTAGATAACTACGATACGGGAGGGCT
Off-target duplex	116 bp	TCCGGTTCCCAACGATCAAGGCGAGTTACATGATCCCCCATGTTGTGC<br>AAAAAAGCGGTTAGCTCCTTCGGTCCTCCGATCGTTGTCAGAAGTAAG<br>TTGGCCGCAGTGTTATCACT
GFP plasmid	6389 bp	pCSanDI-HYG-N44
pUC19 plasmid	2686 bp	New England Biolabs (N3041)

```
>pCSanDI-HYG-N44 [length=6389]
GACGGATCGGGAGATCTCCCGATCCCCTATGGTGCACTCTCAGTACAATCTGCTCTGATGCCGCATAGTTAAGCC
AGTATCTGCTCCCTGCTTGTGTGTTGGAGGTCGCTGAGTAGTGCGCGAGCAAAATTTAAGCTACAACAAGGCAAG
GCTTGACCGACAATTGCATGAAGAATCTGCTTAGGGTTAGGCGTTTTGCGCTGCTTCGCGATGTACGGGCCAGAT
ATACGCGTTGACATTGATTATTGACTAGTTATTAATAGTAATCAATTACGGGGTCATTAGTTCATAGCCCATATA
TGGAGTTCCGCGTTACATAACTTACGGTAAATGGCCCGCCTGGCTGACCGCCCAACGACCCCCGCCCATTGACGT
CAATAATGACGTATGTTCCCATAGTAACGCCAATAGGGACTTTCCATTGACGTCAATGGGTGGAGTATTTACGGT
AAACTGCCCACTTGGCAGTACATCAAGTGTATCATATGCCAAGTACGCCCCCTATTGACGTCAATGACGGTAAAT
GGCCCGCCTGGCATTATGCCCAGTACATGACCTTATGGGACTTTCCTACTTGGCAGTACATCTACGTATTAGTCA
TCGCTATTACCATGGTGATGCGGTTTTGGCAGTACATCAATGGGCGTGGATAGCGGTTTGACTCACGGGGATTTC
CAAGTCTCCACCCCATTGACGTCAATGGGAGTTTGTTTTGGCACCAAAATCAACGGGACTTTCCAAAATGTCGTA
ACAACTCCGCCCCATTGACGCAAATGGGCGGTAGGCGTGTACGGTGGGAGGTCTATATAAGCAGAGCTCTCTGGC
TAACTAGAGAACCCACTGCTTACTGGCTTATCGAAATTAATACGACTCACTATAGGGAGACCCAAGCTGGCTAGC
GTTTAAACTTAAGCTTGGTACCGAGCTCGGATCCACCGGTCGCCACCATGGTGAGCAAGGGCGAGGAGCTGTTCA
CCGGGGTGGTGCCCATCCTGGTCGAGCTGGACGGCGACGTAAACGGCCACAAGTTCAGCGTGTCCGGCGAGGGCG
AGGGCGATGCCACCTACGGCAAGCTGACCCTGAAGTTCATCTGCACCACCGGCAAGCTGCCCGTGCCCTGGCCCA
CCCTCGTGACCACCCTGACCTACGGCGTGCAGTGCTTCAGCCGCTACCCCGACCACATGAAGCAGCACGACTTCT
TCAAGTCCGCCATGCCCGAAGGCTACGTCCAGGAGCGCACCATCTTCTTCAAGGACGACGGCAACTACAAGACCC
GCGCCGAGGTGAAGTTCGAGGGCGACACCCTGGTGAACCGCATCGAGCTGAAGGGCATCGACTTCAAGGAGGACG
GCAACATCCTGGGGCACAAGCTGGAGTACAACTACAACAGCCACAACGTCTATATCATGGCCGACAAGCAGAAGA
ACGGCATCAAGGTGAACTTCAAGATCCGCCACAACATCGAGGACGGCAGCGTGCAGCTCGCCGACCACTACCAGC
AGAACACCCCCATCGGCGACGGCCCCGTGCTGCTGCCCGACAACCACTACCTGAGCACCCAGTCCGCCCTGAGCA
```

AAGACCCCAACGAGAAGCGCGATCACATGGTCCTGCTGGAGTTCGTGACCGCCGCCGGGATCACTCTCGGCATGG
ACGAGCTGTACAAGAAGCTTAGCCATGGCTTCCCGCCGGAGGTGGAGGAGCAGGATGATGGCACGCTGCCCATGT
CTTGTGCCCAGGAGAGCGGGATGGACCGTCACCCTGCAGCCTGTGCTTCTGCTAGGATCAATGTGTAGGCGGCCG
CTCGAGTCTAGGGTCCCATCGGAATTCTCGATGCATGCCTCGACTGTGCCTTCTAGTTGCCAGCCATCTGTTGTT
TGCCCCTCCCCCGTGCCTTCCTTGACCCTGGAAGGTGCCACTCCCACTGTCCTTTCCTAATAAAATGAGGAAATT
GCATCGCATTGTCTGAGTAGGTGTCATTCTATTCTGGGGGGTGGGGTGGGGCAGGACAGCAAGGGGGAGGATTGG
GAAGACAATAGCAGGCATGCTGGGGATGCGGTGGGCTCTATGGCTTCTGAGGCGGAAAGAACCAGCTGGGGCTCT
AGGGGGTATCCCCACGCGCCCTGTAGCGGCGCATTAAGCGCGGCGGGTGTGGTGGTTACGCGCAGCGTGACCGCT
ACACTTGCCAGCGCCCTAGCGCCCGCTCCTTTCGCTTTCTTCCCTTCCTTTCTCGCCACGTTCGCCGGCTTTCCC
CGTCAAGCTCTAAATCGGGGGCTCCCTTTAGGGTTCCGATTTAGTGCTTTACGGCACCTCGACCCCAAAAAACTT
GATTAGGGTGATGGTTCACGTACCTAGAAGTTCCTATTCCGAAGTTCCTATTCTCTAGATACCGGGTAGGGGAGG
CGCTTTTCCCAAGGCAGTCTGGAGCATGCGCTTTAGCAGCCCCGCTGGGCACTTGGCGCTACACAAGTGGCCTCT
GGCCTCGCACACATTCCACATCCACCGGTAGGCGCCAACCGGCTCCGTTCTTTGGTGGCCCCTTCGCGCCACCTT
CTACTCCTCCCCTAGTCAGGAAGTTCCCCCCCGCCCCGCAGCTCGCGTCGTGCAGGACGTGACAAATGGAAGTAG
CACGTCTCACTAGTCTCGTGCAGATGGACAGCACCGCTGAGCAATGGAAGCGGGTAGGCCTTTGGGGCAGCGGCC
AATAGCAGCTTTGCTCCTTCGCTTTCTGGGCTCAGAGGCTGGGAAGGGGTGGGTCCGGGGGCGGGCTCAGGGGCG
GGCTCAGGGGCGGGGCGGGCGCCCGAAGGTCCTCCGGAGGCCCGGCATTCTGCACGCTTCAAAAGCGCACGTCTG
CCGCGCTGTTCTCCTCTTCCTCATCTCCGGGCCTTTCGACCGATCCAGCCGCCACCATGAAAAAGCCTGAACTCA
CCGCGACGTCTGTCGAGAAGTTTCTGATCGAAAAGTTCGACAGCGTCTCCGACCTGATGCAGCTCTCGGAGGGCG
AAGAATCTCGTGCTTTCAGCTTCGATGTAGGAGGGCGTGGATATGTCCTGCGGGTAAATAGCTGCGCCGATGGTT
TCTACAAAGATCGTTATGTTTATCGGCACTTTGCATCGGCCGCGCTCCCGATTCCGGAAGTGCTTGACATTGGGG
AGTTCAGCGAGAGCCTGACCTATTGCATCTCCCGCCGTGCACAGGGTGTCACGTTGCAAGACCTGCCTGAAACCG
AACTGCCCGCTGTTCTGCAGCCGGTCGCGGAGGCCATGGATGCGATCGCTGCGGCCGATCTTAGCCAGACGAGCG
GGTTCGGCCCATTCGGACCGCAAGGAATCGGTCAATACACTACATGGCGTGATTTCATATGCGCGATTGCTGATC
CCCATGTGTATCACTGGCAAACTGTGATGGACGACACCGTCAGTGCGTCCGTCGCGCAGGCTCTCGATGAGCTGA
TGCTTTGGGCCGAGGACTGCCCCGAAGTCCGGCACCTCGTGCACGCGGATTTCGGCTCCAACAATGTCCTGACGG
ACAATGGCCGCATAACAGCGGTCATTGACTGGAGCGAGGCGATGTTCGGGGATTCCCAATACGAGGTCGCCAACA
TCTTCTTCTGGAGGCCGTGGTTGGCTTGTATGGAGCAGCAGACGCGCTACTTCGAGCGGAGGCATCCGGAGCTTG
CAGGATCGCCGCGGCTCCGGGCGTATATGCTCCGCATTGGTCTTGACCAACTCTATCAGAGCTTGGTTGACGGCA
ATTTCGATGATGCAGCTTGGGCGCAGGGTCGATGCGACGCAATCGTCCGATCCGGAGCCGGGACTGTCGGGCGTA
CACAAATCGCCCGCAGAAGCGCGGCCGTCTGGACCGATGGCTGTGTAGAAGTACTCGCCGATAGTGGAAACCGAC
GCCCCAGCACTCGTCCGAGGGCAAAGGAATAGCACGTACTACGAGATTTCGATTCCACCGCCGCCTTCTATGAAA
GGTTGGGCTTCGGAATCGTTTTCCGGGACGCCGGCTGGATGATCCTCCAGCGCGGGGATCTCATGCTGGAGTTCT
TCGCCCACCCCAACTTGTTTATTGCAGCTTATAATGGTTACAAATAAAGCAATAGCATCACAAATTTCACAAATA
AAGCATTTTTTTCACTGCATTCTAGTTGTGGTTTGTCCAAACTCATCAATGTATCTTATCATGTCTGTATACCGT
CGACCTCTAGCTAGAGCTTGGCGTAATCATGGTCATAGCTGTTTCCTGTGTGAAATTGTTATCCGCTCACAATTC
CACACAACATACGAGCCGGAAGCATAAAGTGTAAAGCCTGGGGTGCCTAATGAGTGAGCTAACTCACATTAATTG
CGTTGCGCTCACTGCCCGCTTTCCAGTCGGGAAACCTGTCGTGCCAGCTGCATTAATGAATCGGCCAACGCGCGG
GGAGAGGCGGTTTGCGTATTGGGCGCTCTTCCGCTTCCTCGCTCACTGACTCGCTGCGCTCGGTCGTTCGGCTGC
GGCGAGCGGTATCAGCTCACTCAAAGGCGGTAATACGGTTATCCACAGAATCAGGGGATAACGCAGGAAAGAACA
TGTGAGCAAAAGGCCAGCAAAAGGCCAGGAACCGTAAAAAGGCCGCGTTGCTGGCGTTTTTCCATAGGCTCCGCC

CCCCTGACGAGCATCACAAAAATCGACGCTCAAGTCAGAGGTGGCGAAACCCGACAGGACTATAAAGATACCAGG
CGTTTCCCCCTGGAAGCTCCCTCGTGCGCTCTCCTGTTCCGACCCTGCCGCTTACCGGATACCTGTCCGCCTTTC
TCCCTTCGGGAAGCGTGGCGCTTTCTCATAGCTCACGCTGTAGGTATCTCAGTTCGGTGTAGGTCGTTCGCTCCA
AGCTGGGCTGTGTGCACGAACCCCCCGTTCAGCCCGACCGCTGCGCCTTATCCGGTAACTATCGTCTTGAGTCCA
ACCCGGTAAGACACGACTTATCGCCACTGGCAGCAGCCACTGGTAACAGGATTAGCAGAGCGAGGTATGTAGGCG
GTGCTACAGAGTTCTTGAAGTGGTGGCCTAACTACGGCTACACTAGAAGAACAGTATTTGGTATCTGCGCTCTGC
TGAAGCCAGTTACCTTCGGAAAAAGAGTTGGTAGCTCTTGATCCGGCAAACAAACCACCGCTGGTAGCGGTGGTT
TTTTTGTTTGCAAGCAGCAGATTACGCGCAGAAAAAAAGGATCTCAAGAAGATCCTTTGATCTTTTCTACGGGGT
CTGACGCTCAGTGGAACGAAAACTCACGTTAAGGGATTTTGGTCATGAGATTATCAAAAAGGATCTTCACCTAGA
TCCTTTTAAATTAAAAATGAAGTTTTAAATCAATCTAAAGTATATATGAGTAAACTTGGTCTGACAGTTACCAAT
GCTTAATCAGTGAGGCACCTATCTCAGCGATCTGTCTATTTCGTTCATCCATAGTTGCCTGACTCCCCGTCGTGT
AGATAACTACGATACGGGAGGGCTTACCATCTGGCCCCAGTGCTGCAATGATACCGCGAGACCCACGCTCACCGG
CTCCAGATTTATCAGCAATAAACCAGCCAGCCGGAAGGGCCGAGCGCAGAAGTGGTCCTGCAACTTTATCCGCCT
CCATCCAGTCTATTAATTGTTGCCGGGAAGCTAGAGTAAGTAGTTCGCCAGTTAATAGTTTGCGCAACGTTGTTG
CCATTGCTACAGGCATCGTGGTGTCACGCTCGTCGTTTGGTATGGCTTCATTCAGCTCCGGTTCCCAACGATCAA
GGCGAGTTACATGATCCCCCATGTTGTGCAAAAAAGCGGTTAGCTCCTTCGGTCCTCCGATCGTTGTCAGAAGTA
AGTTGGCCGCAGTGTTATCACTCATGGTTATGGCAGCACTGCATAATTCTCTTACTGTCATGCCATCCGTAAGAT
GCTTTTCTGTGACTGGTGAGTACTCAACCAAGTCATTCTGAGAATAGTGTATGCGGCGACCGAGTTGCTCTTGCC
CGGCGTCAATACGGGATAATACCGCGCCACATAGCAGAACTTTAAAAGTGCTCATCATTGGAAAACGTTCTTCGG
GGCGAAAACTCTCAAGGATCTTACCGCTGTTGAGATCCAGTTCGATGTAACCCACTCGTGCACCCAACTGATCTT
CAGCATCTTTTACTTTCACCAGCGTTTCTGGGTGAGCAAAAACAGGAAGGCAAAATGCCGCAAAAAAGGGAATAA
GGGCGACACGGAAATGTTGAATACTCATACTCTTCCTTTTTCAATATTATTGAAGCATTTATCAGGGTTATTGTC
TCATGAGCGGATACATATTTGAATGTATTTAGAAAAATAAACAAATAGGGGTTCCGCGCACATTTCCCCGAAAAG
TGCCACCTGACGTC

S - 4.2 Thermal melting

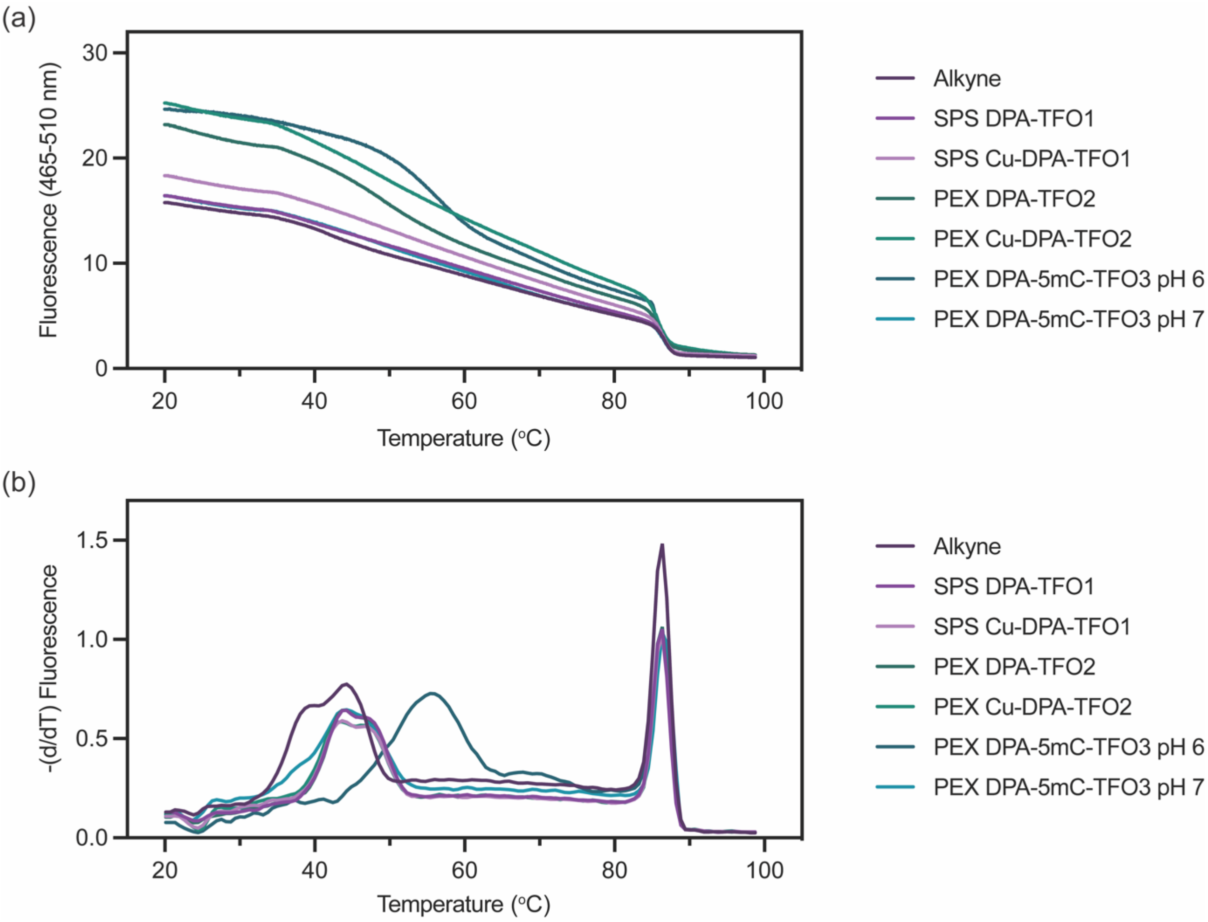

Figure S - 19. (a) Plot of fluorescence readings taken during thermal melting. (b) First negative derivative of fluorescence measurements.

S - 4.3 Triplex formation

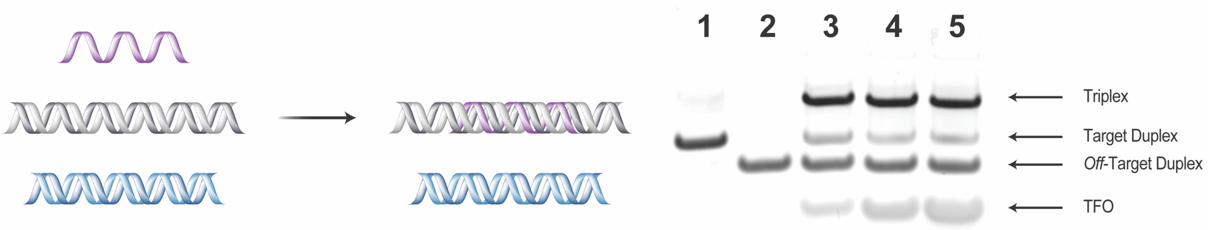

Figure S - 20. TFO (purple) hybridises with target duplex (grey) to form triplex, while *off*-target (blue) remains in duplex state. Lane 1: 57 bp target duplex. Lane 2: *Off*-target duplex. Lanes 3-5: Triplex with 10, 25 and 50 equivalents of TFO to duplex.

S - 4.4 DPA-TFO hybrids PAGE

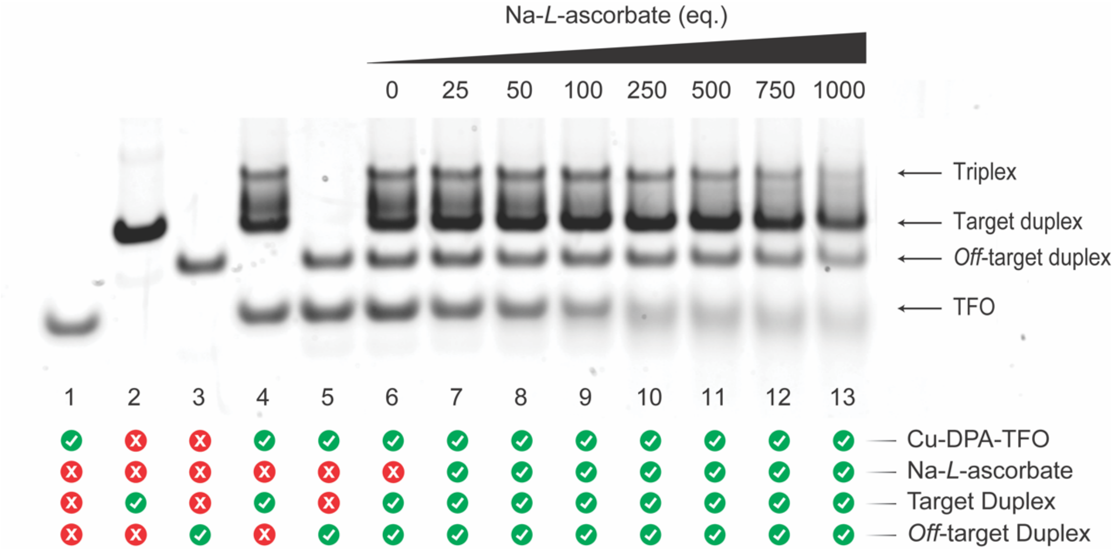

Figure S - 21. 20% native PAGE showing the targeted DNA damage inflicted by SPS Cu(II)-DPA-TFO1. Lane 1: TFO (32 nt). Lane 2: Target duplex (57 bp). Lane 3: *Off*-target duplex (40 bp). Lane 4: TFO and target duplex hybridise to form triplex. Lane 5: TFO does not bind with *off*-target duplex. Lane 6-15: Target and *off*-target duplexes incubated with Cu(II)-DPA-TFO (25 eq.) and 0-1000 eq. Na-*L*-ascorbate. TFO only binds with its target sequence inflicting DNA damage, while *off*-target remains relatively intact.

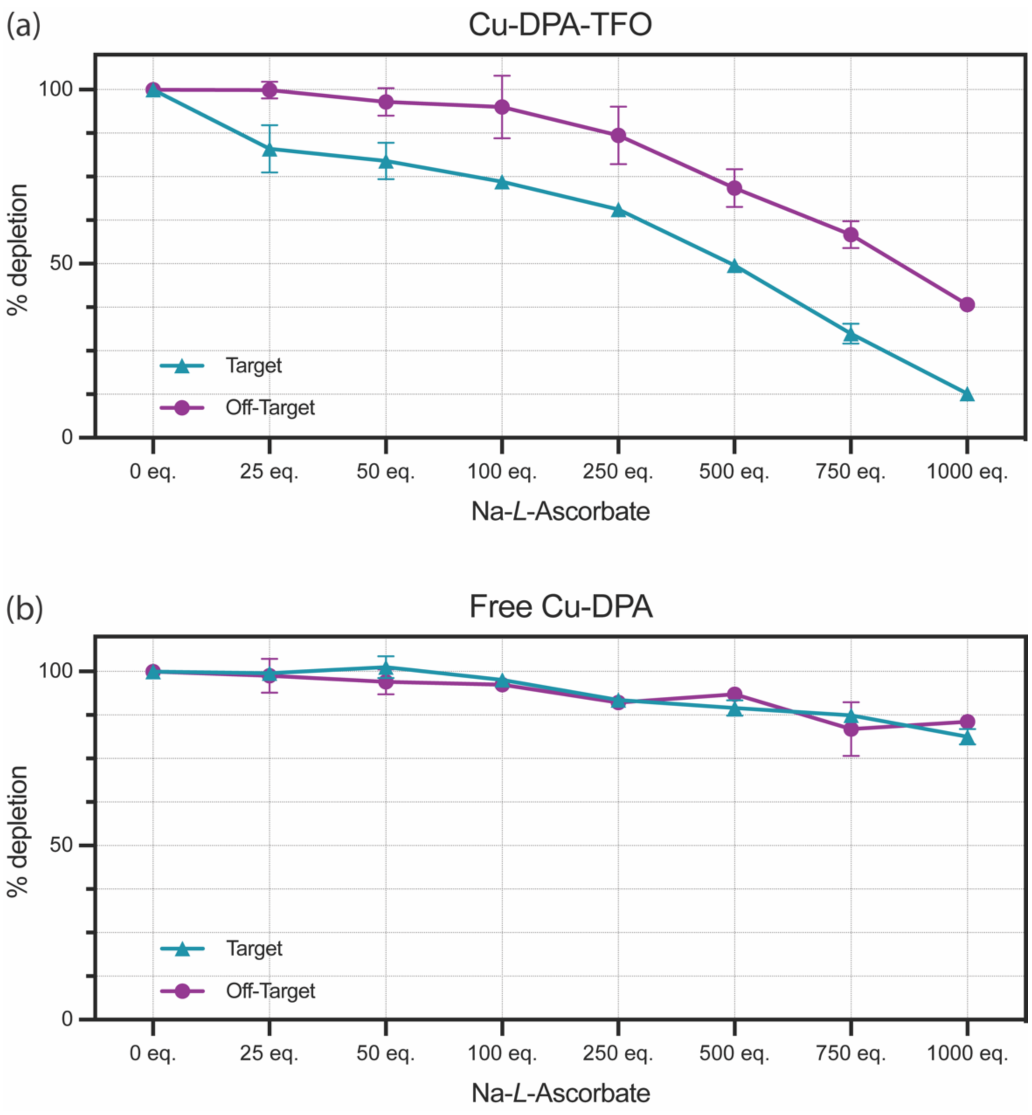

Figure S - 22. Band densitometry of DNA cleavage inflicted by Cu-DPA-TFO (a), and free Cu-DPA with free alkyne TFO (b).

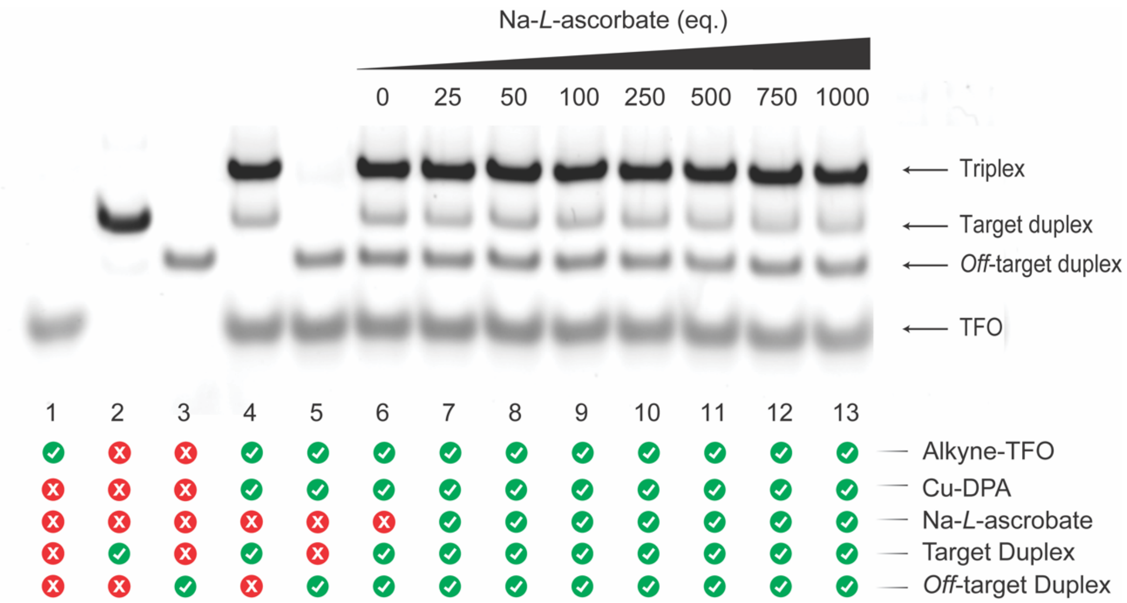

Figure S - 23. 20% native PAGE displaying DNA damage caused by free Cu-DPA and free-alkyne-TFO. Lane 1: Alkyne TFO (32 nt). Lane 2: Target duplex (57 bp). Lane 3: *Off*-target duplex (40 bp). Lane 4: Alkyne-TFO and target duplex hybridise to form triplex. Lane 5: Alkyne-TFO does not bind with *off*-target duplex. Lane 6-15: Target and *off*-target duplexes incubated with free Cu(II)-DPA and free alkyne-TFO (25 eq.) and 0-1000 eq. Na-*L*-ascorbate. No significant DNA damage is observed for either DNA duplex.

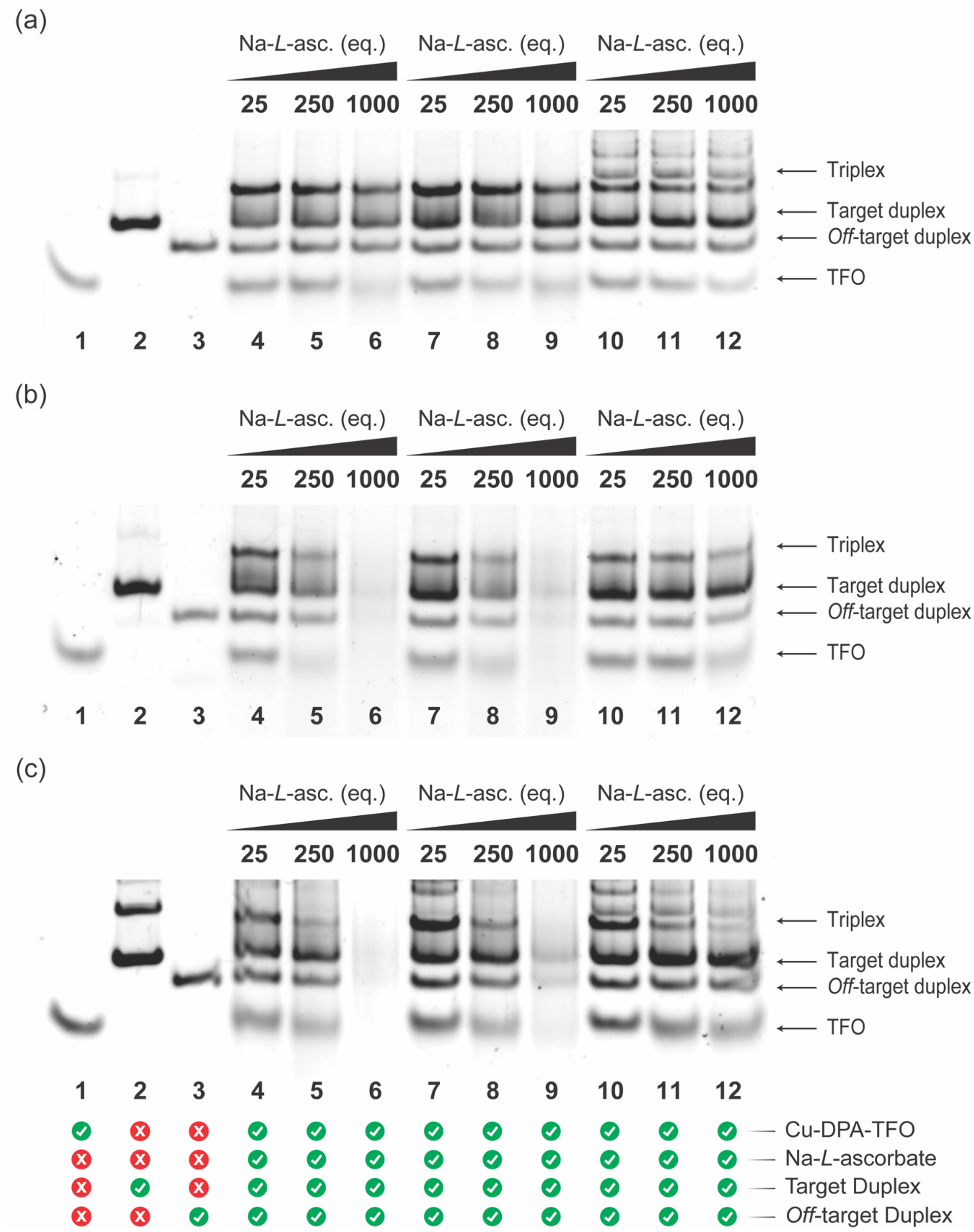

Figure S - 24. Cleavage inflicted by DPA-TFO hybrids at (a) 20 °C, (b) 30 °C and 40 °C (c). Lane 1: TFO (32 nt). Lane 2: Target duplex (57 bp). Lane 3: *Off*-target duplex (40 bp). Lanes 4-12: Target and *off*-target duplexes incubated with Cu(II)-DPA-TFO (25 eq.) for 24, 15 or 2 hours and 25-1000 eq. Na-*L*-ascorbate. Lanes 4-6: 24 hour incubation with 25, 250 and 1000 equivalents of Na-*L*-ascorbate, respectively. Lanes 7-9: 15 hour incubation with 25, 250 and 1000 equivalents of Na-*L*-ascorbate, respectively. Lanes 10-12: 2 hour incubation with 25, 250 and 1000 equivalents of Na-*L*-ascorbate, respectively.

S - 4.5 qPCR assay for analysis of target and *off*-target DNA cleavage

The qPCR assay for the analysis of DNA damage inflicted upon a target and an *off*-target DNA sequence required significant planning and optimisation. The target duplex was a 113 bp amplicon from the GFP gene (forward: 5′-AAAGGGAGCCCCCGATTTAG-3′ and reverse: 5′-GTGACCGCTACACTTGCCA-3′) that contained the TFO recognition sequence (*i.e.* 5′-GGCGAGAAAGGAAGGGAAGAAAGCGAAAGGAG-3′). An *off*-target consisting of the same 113 bp GFP sequence but with a scrambled TFO binding site was considered, but this would prevent target and *off*-target duplexes being present simultaneously in the qPCR samples. Consequently, it was decided that an alternative sequence from another DNA plasmid would make a more suitable *off*-target duplex for qPCR analysis. However, many factors needed to be considered in the selection of the *off*-target: i.) The 113 bp GFP primers (forward: 5′-AAAGGGAGCCCCCGATTTAG-3′ and reverse: 5′-GTGACCGCTACACTTGCCA-3′) used to amplify the target duplex could not have a binding site within the *off*-target sequence, and vice versa; ii.) the *off*-target duplex must not contain the TFO recognition site (*i.e.* 5′-GGCGAGAAAGGAAGGGAAGAAAGCGAAAGGAG-3′); iii.) the target and *off*-target primers must have the same annealing temperature to allow qPCR with both amplicons be performed simultaneously; iv.) the target and *off*-target amplicons must have similar amplification efficiencies to allow for an accurate comparison, and v.) ideally, both amplicons would be of a similar length to allow free nuclease controls be accurately evaluated. A 116 bp region of the pUC19 plasmid was found to satisfy these criteria.

S - 4.6 qPCR analysis of catalytic activity with target and *off*-target

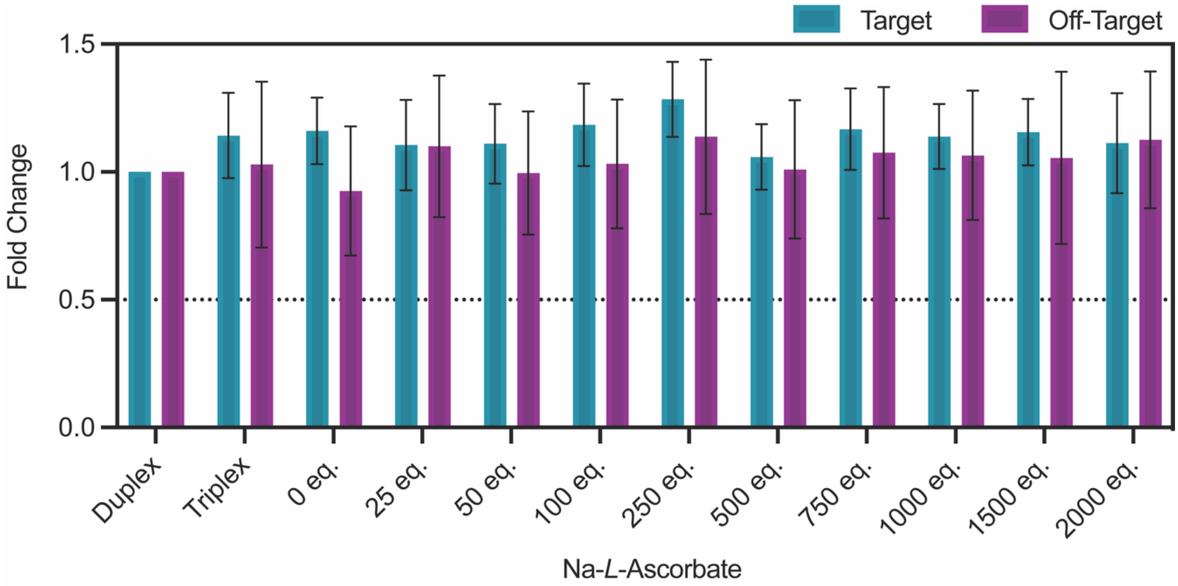

Figure S - 25. Target and *off*-target DNA duplexes were incubated with free Cu-DPA and free alkyne-modified TFO. The fold change in intact DNA concentration was plotted. No significant damage has occurred to either DNA duplex.

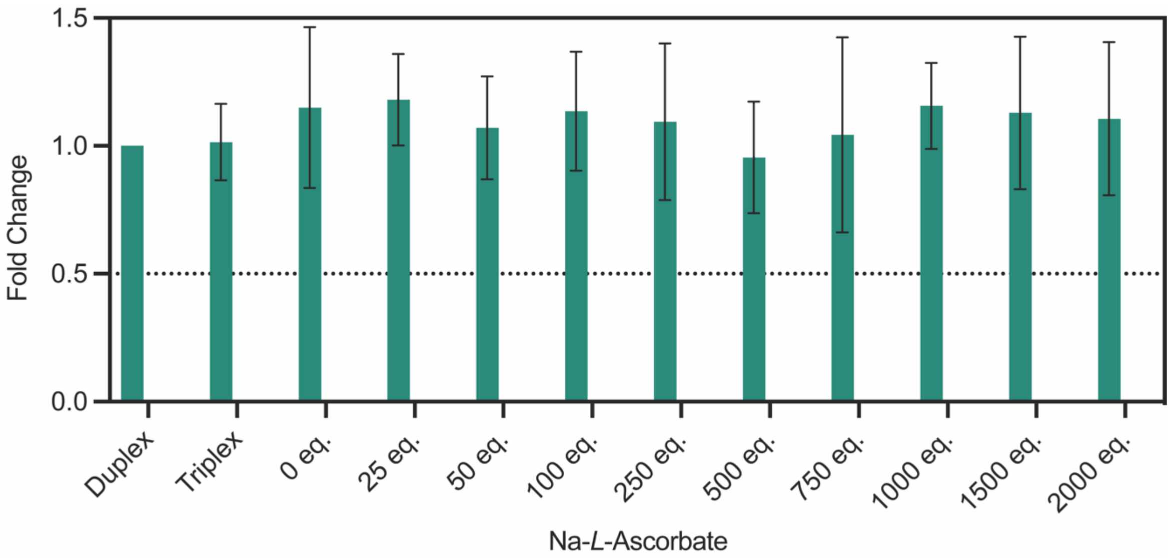

Figure S - 26. qPCR was performed to quantify the DNA damage that occurred to the target duplex in the presence of Cu-DPA-TFO and the ROS species tiron. No significant damage was observed, which confirms that the DNA damage inflicted by the DPA-TFO hybrid is responsible for inhibiting DNA amplification during PCR.

S - 4.7 Plasmid studies

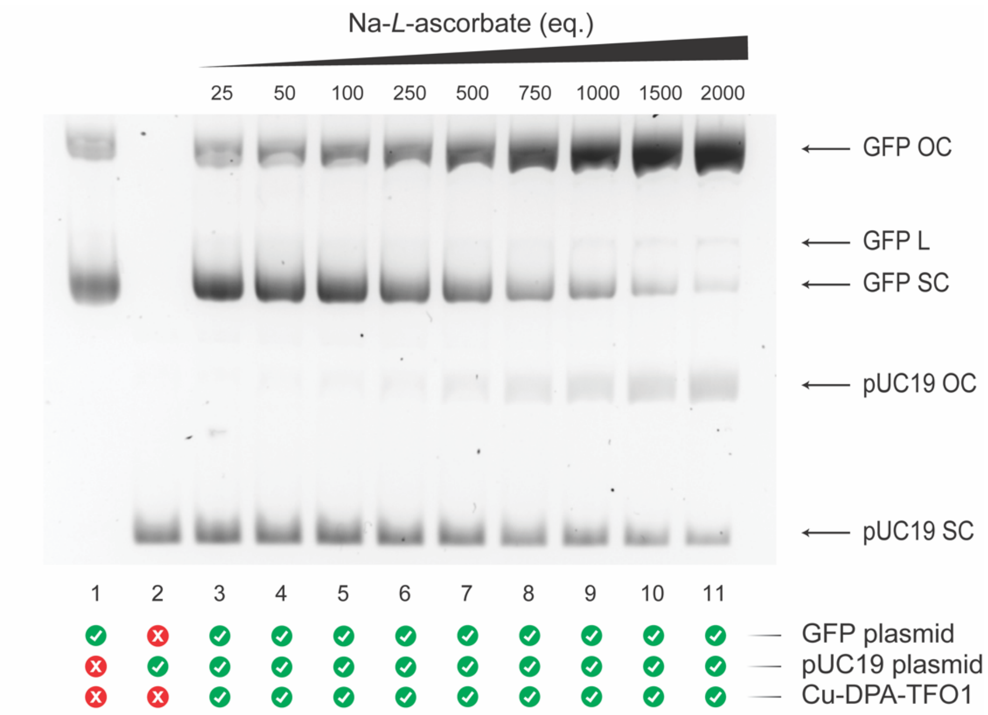

Figure S - 27. Agarose (1%) gel electrophoretic analysis of the damage inflicted upon target and *off*-target supercoiled DNA plasmids by SPS Cu-DPA-TFO1. Lane 1: Supercoiled (SC) GFP (6389 bp). Lane 2: Supercoiled (SC) pUC19 (2686 bp). Lane 6-11: GFP and pUC19 with 25 eq. Cu-DPA-TFO1 and 25-2000 eq. Na-*L*-ascorbate.

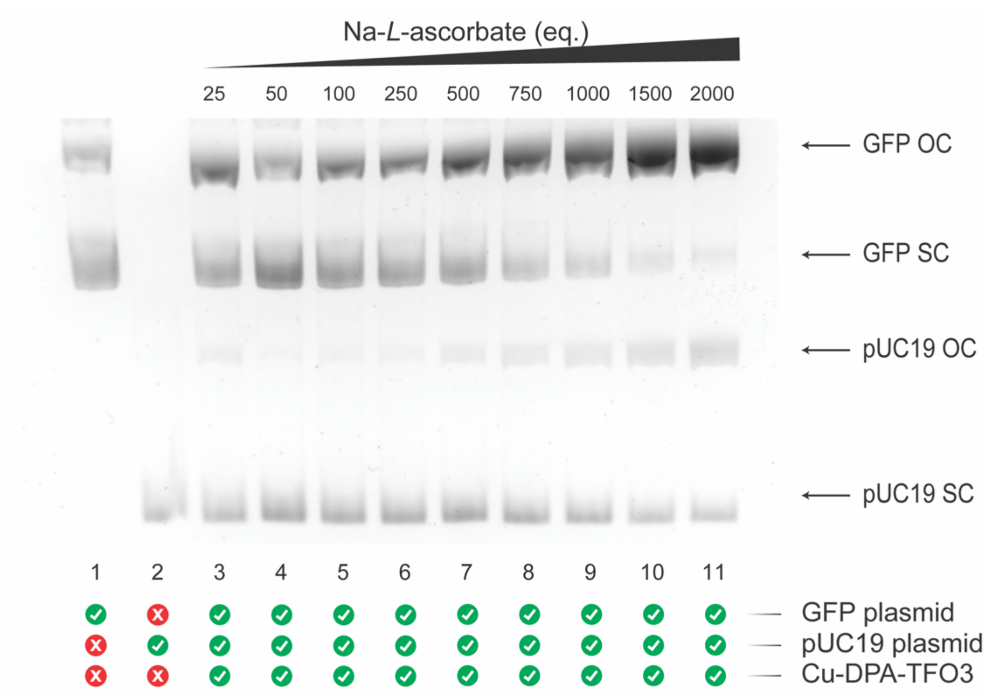

Figure S - 28. 1% agarose gel analysis of enzymatically synthesised TFO3. Lane 1: Supercoiled (SC) GFP (6389 bp). Lane 2: Supercoiled (SC) pUC19 (2686 bp). Lane 3-11: GFP and pUC19 with 25 eq. Cu-DPA-5mC-TFO3 and 25-2000 eq. Na-*L*-ascorbate.

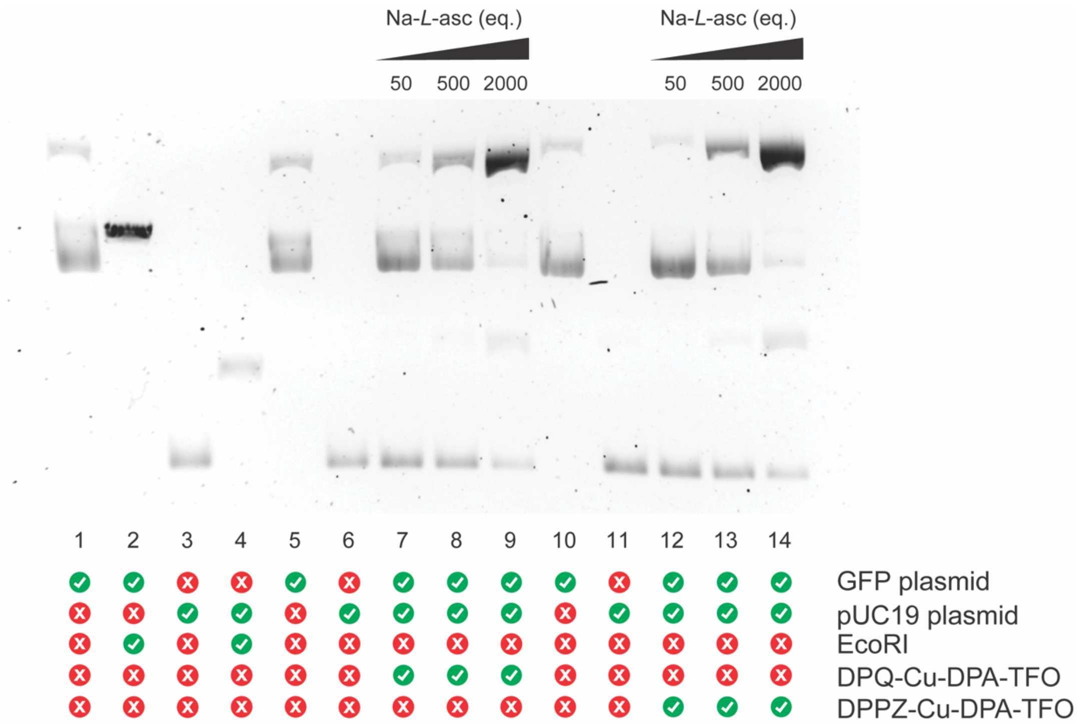

Figure S - 29. 1% agarose gel showing targeted DNA cleavage for Cu-DPA-TFO with designer intercalators, DPQ and DPPZ. Lane 1: Supercoiled (SC) GFP (6389 bp). Lane 2: GFP linearised (L) with EcoRI. Lane 3: Supercoiled (SC) pUC19 (2686 bp). Lane 4: pUC19 linearised (L) with EcoRI. Lane 5: GFP control. Lane 6: pUC19 control. Lane 7-9: GFP and pUC19 with 25 eq. DPQ-Cu-DPA-TFO and 50,500 or 2000 eq. Na-*L*-ascorbate. Lane 10: GFP control. Lane 11: pUC19 control. Lanes 12-14: GFP and pUC19 with 25 eq. DPPZ-Cu-DPA-TFO and 50,500 or 2000 eq. Na-*L*-ascorbate.

S - 4.8 Sequencing

Control forward run 1

```
TCCCAGCCTTGTTGTAGCGCCAAGTGCCCAGCGGGGCTGCTAAAGCGCATGCTCCAGACTGCCTTGGGAAAAGCG
CCTCCCCTACCCGGTATCTAGAGAATAGGAACTTCGGAATAGGAACTTCTAGGTACGTGAACCATCACCCTAATC
AAGTTTTTTGGGGTCGAGGTGCCGTAAAGCACTAAATCGGAACCCTAAAGGGAGCCCCCGATTTAGAGCTTGACG
GGGAAAGCCGGCGAACGTGGCGAGAAAGGAAGGGAAGAAAGCGAAAGGAGCGGGCGCTAGGGCGCTGGCAAGTGT
AGCGGTCACGCTGCGCGTAACCACCACACCCGCCGCGCTTAATGCGCCGCTACAGGGCGCGTGGGGATACCCCCT
AGAGCCCCAGCTGGTTCTTTCCGCCTCAGAAGCCATAGAGCCCACCGCATCCCCAGCATGCCTGCTATTGTCTTC
CCAATCCTCCCCCTTGCTGTCCTGCCCCACCCCACCCCCCAGAATAGAATGACACCTACTCAGACAATGCGATGC
AATTTCCTCATTTTATTAGGAAAGGACAGTGGGAGTGGCACCTTCCAGGGTCAAGGAAGGCACGGGGGAGGGGCA
AACAACAGATGGCTGGCAACTAGAAGGCACAGTCGAGGCATGCATCGAGAATTCCGATGGGACCCTAGACTCGAG
CGGCCGCCTACACATTGATCCTAGCAGAAGCACAGGCTGCAGGGTGACGGTCCATCCCGCTCTCCTGGGCACAAG
ACATGGGCAGCGTGCCATCATCCTGCTCCTCCACCTCCGGCGGGAAGCCATGGCTAAGCTTCTTGTACAGCTCGT
CCATGCCGAGAGTGATCCCGGCGGCGGTCACGAACTCCAGCAGGACCATGTGATCGCGCTTCTCGTTGGGGTCTT
TGCTCAGGGCGGACTGGGTGCTCAGGTAGTGGTTGTCGGGCAGCAGCACGGGGCCGTCGCCGATGGGGGTGTTCT
GCTGGTAGTGGTCGGCGAGCTGCACGCTGCCGTCCTCGATGTTGTGGCGGATCTTGAAGTTCACCTTGATGCCGT
TCTTCTGCTTGTCGGCATGATATAGACGTTGTGGCTGTTGTAATTGTACTCCAGCTTGTGCCCCAGAATGTTGCC
GTCCTCCTTGAAATCGATGCCCTTCACCTCAAGCGGTTCCCAGGGTGTCGCCCCCAAATTTCCCCCGGGCGGGGC
TTGGAATTTCCTCGCCCTTAGAAAAAAAGGGCGCCCCGGAATAACCTTCGGGAGGCGGAATTAAAAAAAAAGGTG
TTTCTTGGGCGGGGGAAGGCTAAAACTCACCCCCCAAGTCAGGGTGTCCAAGGTGGGCAGGCCGGGAACTTTCCG
GGGGGGGAAAAAATTAAGGAAATTTGCAAAGGGATCACCCCCCCCCCCCCCACCAAATTAGGTTTTTTTTTCCCT
TTCACAAGAACCCCGAAACCCCCCTCCCGGGGGGGGGGGGGGCGTTTAAAAATTGCCCTTTTTTTTTATAATTTT
AATTAAACCACCCCCCCCCGCGCGGTGGTTGGAGAGGGGTGAGGGGGGGGGGGGAAAAGTCTAGAAAATAAAGAT
ATCTAAGTATAAATATATATAATGATTCAGCATTTTGATTATATCAATAGTAATATATCGTGATATATATTAATA
TGAGATTATTA
```

Control forward run 2

```
TGTTGTAGCGCCAAGTGCCCAGCGGGGCTGCTAAAGCGCATGCTCCAGACTGCCTTGGGAAAAGCGCCTCCCCTA
CCCGGTATCTAGAGAATAGGAACTTCGGAATAGGAACTTCTAGGTACGTGAACCATCACCCTAATCAAGTTTTTT
GGGGTCGAGGTGCCGTAAAGCACTAAATCGGAACCCTAAAGGGAGCCCCCGATTTAGAGCTTGACGGGGAAAGCC
GGCGAACGTGGCGAGAAAGGAAGGGAAGAAAGCGAAAGGAGCGGGCGCTAGGGCGCTGGCAAGTGTAGCGGTCAC
GCTGCGCGTAACCACCACACCCGCCGCGCTTAATGCGCCGCTACAGGGCGCGTGGGGATACCCCCTAGAGCCCCA
GCTGGTTCTTTCCGCCTCAGAAGCCATAGAGCCCACCGCATCCCCAGCATGCCTGCTATTGTCTTCCCAATCCTC
CCCCTTGCTGTCCTGCCCCACCCCACCCCCCAGAATAGAATGACACCTACTCAGACAATGCGATGCAATTTCCTC
ATTTTATTAGGAAAGGACAGTGGGAGTGGCACCTTCCAGGGTCAAGGAAGGCACGGGGGAGGGGCAAACAACAGA
TGGCTGGCAACTAGAAGGCACAGTCGAGGCATGCATCGAGAATTCCGATGGGACCCTAGACTCGAGCGGCCGCCT
ACACATTGATCCTAGCAGAAGCACAGGCTGCAGGGTGACGGTCCATCCCGCTCTCCTGGGCACAAGACATGGGCA
GCGTGCCATCATCCTGCTCCTCCACCTCCGGCGGGAAGCCATGGCTAAGCTTCTTGTACAGCTCGTCCATGCCGA
GAGTGATCCCGGCGGCGGTCACGAACTCCAGCAGGACCATGTGATCGCGCTTCTCGTTGGGGTCTTTGCTCAGGG
CGGACTGGGTGCTCAGGTAGTGGTTGTCGGGCAGCAGCACGGGGCCGTCGCCGATGGGGGTGTTCTGCTGGTAGT
GGTCGGCGAGCTGCACGCTGCCGTCCTCGATGTTGTGGCGGATCTTGAAGTTCACCTTGATGCCGTTCTTCTGCT
TGTCGGCCATGATATAGACGTTGTGGCTGTTGTAGTTGTACTCCAGCTTGTGCCCCAGGATGTTGCCGTCCTCCT
TGAAGTCGATGCCCTTCAGCTCGATGCGGTTCACCAGGGTGTCGCCCTCGAACTTCACCTCGGCGCGGGTCTTGT
AATTGCCGTCGTCCTTGAAGAGATG
```

Cu-DPA forward run 1

```
CTTGTGTAGCGCCAAGTGCCCAGCGGGGCTGCTAAAGCGCATGCTCCAGACTGCCTTGGGAAAAGCGCCTCCCCT
ACCCGGTATCTAGAGAATAGGAACTTCGGAATAGGAACTTCTAGGTACGTGAACCATCACCCTAATCAAGTTTTT
TGGGGTCGAGGTGCCGTAAAGCACTAAATCGGAACCCTAAAGGGAGCCCCCGATTTAGAGCTTGACGGGGAAAGC
CGGCGAACGTGGCGAGAAAGGAAGGGAAGAAAGCGAAAGGAGCGGGCGCTAGGGCGCTGGCAAGTGTAGCGGTCA
CGCTGCGCGTAACCACCACACCCGCCGCGCTTAATGCGCCGCTACAGGGCGCGTGGGGATACCCCCTAGAGCCCC
AGCTGGTTCTTTCCGCCTCAGAAGCCATAGAGCCCACCGCATCCCCAGCATGCCTGCTATTGTCTTCCCAATCCT
CCCCCTTGCTGTCCTGCCCCACCCCACCCCCCAGAATAGAATGACACCTACTCAGACAATGCGATGCAATTTCCT
CATTTTATTAGGAAAGGACAGTGGGAGTGGCACCTTCCAGGGTCAAGGAAGGCACGGGGGAGGGGCAAACAACAG
ATGGCTGGCAACTAGAAGGCACAGTCGAGGCATGCATCGAGAATTCCGATGGGACCCTAGACTCGAGCGGCCGCC
TACACATTGATCCTAGCAGAAGCACAGGCTGCAGGGTGACGGTCCATCCCGCTCTCCTGGGCACAAGACATGGGC
```

AGCGTGCCATCATCCTGCTCCTCCACCTCCGGCGGGAAGCCATGGCTAAGCTTCTTGTACAGCTCGTCCATGCCG
AGAGTGATCCCGGCGGCGGTCACGAACTCCAGCAGGACCATGTGATCGCGCTTCTCGTTGGGGTCTTTGCTCAGG
GCGGACTGGGTGCTCAGGTAGTGGTTGTCGGGCAGCAGCACGGGGCCGTCGCCGATGGGGGTGTTCTGCTGGTAG
TGGTCGGCGAGCTGCACGCTGCCGTCCTCGATGTTGTGGCGGATCTTGAAGTTCACCTTGATGCCGTTCTTCTGC
TTGTCGGCATGATATAGACGTTGTGGCTGTTGTAGTTGTACTCCAGCTTGTGCCCAGGATGTTGCCGTCCTCCTT
GAAATCAAG

Cu-DPA forward run 2

TGTTGTAGCGCCAAGTGCCCAGCGGGGCTGCTAAAGCGCATGCTCCAGACTGCCTTGGGAAAAGCGCCTCCCCTA
CCCGGTATCTAGAGAATAGGAACTTCGGAATAGGAACTTCTAGGTACGTGAACCATCACCCTAATCAAGTTTTTT
GGGGTCGAGGTGCCGTAAAGCACTAAATCGGAACCCTAAAGGGAGCCCCCGATTTAGAGCTTGACGGGGAAAGCC
GGCGAACGTGGCGAGAAAGGAAGGGAAGAAAGCGAAAGGAGCGGGCGCTAGGGCGCTGGCAAGTGTAGCGGTCAC
GCTGCGCGTAACCACCACACCCGCCGCGCTTAATGCGCCGCTACAGGGCGCGTGGGGATACCCCCTAGAGCCCCA
GCTGGTTCTTTCCGCCTCAGAAGCCATAGAGCCCACCGCATCCCCAGCATGCCTGCTATTGTCTTCCCAATCCTC
CCCCTTGCTGTCCTGCCCCACCCCACCCCCCAGAATAGAATGACACCTACTCAGACAATGCGATGCAATTTCCTC
ATTTTATTAGGAAAGGACAGTGGGAGTGGCACCTTCCAGGGTCAAGGAAGGCACGGGGGAGGGGCAAACAACAGA
TGGCTGGCAACTAGAAGGCACAGTCGAGGCATGCATCGAGAATTCCGATGGGACCCTAGACTCGAGCGGCCGCCT
ACACATTGATCCTAGCAGAAGCACAGGCTGCAGGGTGACGGTCCATCCCGCTCTCCTGGGCACAAGACATGGGCA
GCGTGCCATCATCCTGCTCCTCCACCTCCGGCGGGAAGCCATGGCTAAGCTTCTTGTACAGCTCGTCCATGCCGA
GAGTGATCCCGGCGGCGGTCACGAACTCCAGCAGGACCATGTGATCGCGCTTCTCGTTGGGGTCTTTGCTCAGGG
CGGACTGGGTGCTCAGGTAGTGGTTGTCGGGCAGCAGCACGGGGCCGTCGCCGATGGGGGTGTTCTGCTGGTAGT
GGTCGGCGAGCTGCACGCTGCCGTCCTCGATGTTGTGGCGGATCTTGAAGTTCACCTTGATGCCGTTCTTCTGCT
TGTCGGCCATGATATAGACGTTGTGGCTGTTGTAGTTGTACTCCAGCTTGTGCCCCAGGATGTTGCCGTCCTCCT
TGAAGTCGATGCCCTTCAGCTCGATGCGGTTCACCAGGGTGTCGCCC

DPQ-Cu-DPA-TFO forward run 1

TCCGAGCCTTGTGTGCGCCAAGTGCCCAGCGGGGCTGCTAAAGCGCATGCTCCAGACTGCCTTGGGAAAAGCGCC
TCCCCTACCCGGTATCTAGAGAATAGGAACTTCGGAATAGGAACTTCTAGGTACGTGAACCATCACCCTAATCAA
GTTTTTTGGGGTCGAGGTGCCGTAAAGCACTAAATCGGAACCCTAAAGGGAGCCCCCGATTTAGAGCTTGACGGG
GAAAGCCGGCGAACGTGGCGAGAAAGGAAGGGAAGAAAGCGAAAGGAGCGGGCGCTAGGGCGCTGGCAAGTGTAG
CGGTCACGCTGCGCGTAACCACCACACCCGTCGCGCTTAAAGCTCCGCTACAGGGCGCGTGGGATCCCCCCTAC
TTTTTTAACCGGTACTTTCGCCTCCTTCATACTTGCAGTGTCCTTTCAGACCAGGTATTCAAGAAATGCTTGCAC
CTCTCACGGTTTCAGCGTCAGGCCGCAAGTCGCACCTATCTACAATATCTTCTTATAATGAAGATTTGAAAGATT
TCCAGGAAGCAGAGCCGATACGACCAGGTGAAGAGTAATATTGTTTAGTACTTAAAGCCATTTAGGAACCTCTTA
GCATCGCCCAGGTGTTAGGAGTGTTACTCCACCGGATAGTGCACGACCCTTTCACGTGCGGAGCCAATCTGAATT
CTGAGGACCGGCTGACTTGGAGCATACCTGAAATCACGGCTGACATTGTTCGGACAAGCAGTCACAAGTTGCTGT
CAAAGTTCAGGACCGCACTCGAACACCCCCACTTGTGTATAAACTATCATAAGTGACTCGATGTTCACACCTTCA
CACATTTTAGAGAGGTACAGCCAGAAGACCGTAAGACAGTCATCACTCTGCTGAGGATATCCATAACCTGTCGGG
TGTCCAAACAATCATCGTCAGAGGATCACTTCTACAAAGCTGCTGTGAGATGCGAGAACAAGGCGGCTCTTGCGT
AAGTCCTCCTTGGTCGATAACTTACTTGCTACTACCGGCATGGGACGAACGCCCCGATGACAAGATCCTGACCCG
GGGGTCCCAAA

DPQ-Cu-DPA-TFO forward run 2

GCTTGTGTAGCGCCAAGTGCCCAGCGGGGCTGCTAAAGCGCATGCTCCAGACTGCCTTGGGAAAAGCGCCTCCCC
TACCCGGTATCTAGAGAATAGGAACTTCGGAATAGGAACTTCTAGGTACGTGAACCATCACCCTAATCAAGTTTT
TTGGGGTCGAGGTGCCGTAAAGCACTAAATCGGAACCCTAAAGGGAGCCCCCGATTTAGAGCTTGACGGGGAAAG
CCGGCGAACGTGGCGAGAAAGGAAGGGAAGAAAGCGAAAGGAGCGGGCGCTAGGGCGCTGGCAAGTGTAGCGGTC
ACGCTGCGCGTAACCACCACACCCGCCGCGCTTAATGCGCCGCTACAGGGCGCGTGGGGATACCCCCTAGAGCCC
CAGCTGGTTCTTTCCGCCTCAGAAGCCATAGAGCCCACCGCATCCCCAGCATGCCTGCTATTGTCTTCCCAATCC
TCCCCCTTGCTGTCCTGCCCCACCCCACCCCCCAGAATAGAATGACACCTACTCAGACAATGCGATGCAATTTCC
TCATTTTATTAGGAAAGGACAGTGGGAGTGGCACCTTCCAGGGTCAAGGAAGGCACGGGGGAGGGGCAAACAACA
GATGGCTGGCAACTAGAAGGCACAGTCGAGGCATGCATCGAGAATTCCGATGGGACCCTAGACTCGAGCGGCCGC
CTACACATTGATCCTAGCAGAAGCACAGGCTGCAGGGTGACGGTCCATCCCGCTCTCCTGGGCACAAGACATGGG
CAGCGTGCCATCATCCTGCTCCTCCACCTCCGGCGGGAAGCCATGGCTAAGCTTCTTGTACAGCTCGTCCATGCC
GAGAGTGATCCCGGCGGCGGTCACGAACTCCAGCAGGACCATGTGATCGCGCTTCTCGTTGGGGTCTTTGCTCAG
GGCGGACTGGGTGCTCAGGTAGTGGTTGTCGGGCAGCAGCACGGGGCCGTCGCCGATGGGGGTGTTCTGCTGGTA

GTGGTCGGCGAGCTGCACGCTGCCGTCCTCGATGTTGTGGCGGATCTTGAAGTTCACCTTGATGCCGTTCTTCTG
CTTGTCGGCCATGATATAGACGTTGTGGCTGTTGTAGTTGTACTCCAGCTTGTGCCCCAGGATGTTGCCGTCCTC
CTTGAAGTCGATGCCCTTCAGCTCGATGCGGTTCACCAGGGTGTCGCCCTCGAACTT

DPPZ-Cu-DPA-TFO forward run 1

GTTGTAGCGCCAAGTGCCCAGCGGGGCTGCTAAAGCGCATGCTCCAGACTGCCTTGGGAAAAGCGCCTCCCCTAC
CCGGTATCTAGAGAATAGGAACTTCGGAATAGGAACTTCTAGGTACGTGAACCATCACCCTAATCAAGTTTTTTG
GGGTCGAGGTGCCGTAAAGCACTAAATCGGAACCCTAAAGGGAGCCCCCGATTTAGAGCTTGACGGGGAAAGCCG
GCGAACGTGGCGAGAAAGGAAGGGAAGAAAGCGAAAGGAGCGGGCGCTAGGGCGCTGGCAAGTGTAGCGGTCACG
CTGCGCGTAACCACCACACCCGCCGCGCTTAATGCGCCGCTACAGGGCGCGTGGGATACCCCCTAGAGCCCCAG
CTGGTTCTTTCCGCCTCAGAAGCCATAGAGCCCACCGCATCCCCAGCATGCCTGCTATTGTCTTCCCAATCCTCC
CCCTTGCTGTCCTGCCCCACCCCACCCCCCAGAATAGAATGACACCTACTCAGACAATGCGATGCAATTTCCTCA
TTTTATTAGGAAAGGACAGTGGGAGTGGCACCTTCCAGGGTCAAGGAAGGCACGGGGGAGGGGCAAACAACAGAT
GGCTGGCAACTAGAAGGCACAGTCGAGGCATGCATCGAGAATTCCGATGGGACCCTAGACTCGAGCGGCCGCCTA
CACATTGATCCTAGCAGAAGCACAGGCTGCAGGGTGACGGTCCATCCCGCTCTCCTGGGCACAAGACATGGGCAG
CGTGCCATCATCCTGCTCCTCCACCTCCGGCGGGAAGCCATGGCTAAGCTTCTTGTACAGCTCGTCCATGCCGAG
AGTGATCCCGGCGGCGGTCACGAACTCCAGCAGGACCATGTGATCGCGCTTCTCGTTGGGGTCTTTGCTCAGGGC
GGACTGGGTGCTCAGGTAGTGGTTGTCGGGCAGCAGCACGGGGCCGTCGCCGATGGGGGTGTTCTGCTGGTAGTG
GTCGGCGAGCTGCACGCTGCCGTCCTCGATGTTGTGGCGGATCTTGAAGTTCACCTTGATGCCGTTCTTCTGCTT
GTCGGCATGATATAGACGTTGTGGCTGTTGTAATTGTACTCCAGCTTGTGCCCCAGAATGTTGCCGTCCTCCTTG
AAATCGATGCCCTTCACC

DPPZ-Cu-DPA-TFO forward run 2

TTGTTGTAGCGCCAAGTGCCCAGCGGGGCTGCTAAAGCGCATGCTCCAGACTGCCTTGGGAAAAGCGCCTCCCCT
ACCCGGTATCTAGAGAATAGGAACTTCGGAATAGGAACTTCTAGGTACGTGAACCATCACCCTAATCAAGTTTTT
TGGGGTCGAGGTGCCGTAAAGCACTAAATCGGAACCCTAAAGGGAGCCCCCGATTTAGAGCTTGACGGGGAAAGC
CGGCGAACGTGGCGAGAAAGGAAGGGAAGAAAGCGAAAGGAGCGGGCGCTAGGGCGCTGGCAAGTGTAGCGGTCA
CGCTGCGCGTAACCACCACACCCGCCGCGCTTAATGCGCCGCTACAGGGCGCGTGGGATACCCCCTAGAGCCCC
AGCTGGTTCTTTCCGCCTCAGAAGCCATAGAGCCCACCGCATCCCCAGCATGCCTGCTATTGTCTTCCCAATCCT
CCCCCTTGCTGTCCTGCCCCACCCCACCCCCCAGAATAGAATGACACCTACTCAGACAATGCGATGCAATTTCCT
CATTTTATTAGGAAAGGACAGTGGGAGTGGCACCTTCCAGGGTCAAGGAAGGCACGGGGGAGGGGCAAACAACAG
ATGGCTGGCAACTAGAAGGCACAGTCGAGGCATGCATCGAGAATTCCGATGGGACCCTAGACTCGAGCGGCCGCC
TACACATTGATCCTAGCAGAAGCACAGGCTGCAGGGTGACGGTCCATCCCGCTCTCCTGGGCACAAGACATGGGC
AGCGTGCCATCATCCTGCTCCTCCACCTCCGGCGGGAAGCCATGGCTAAGCTTCTTGTACAGCTCGTCCATGCCG
AGAGTGATCCCGGCGGCGGTCACGAACTCCAGCAGGACCATGTGATCGCGCTTCTCGTTGGGGTCTTTGCTCAGG
GCGGACTGGGTGCTCAGGTAGTGGTTGTCGGGCAGCAGCACGGGGCCGTCACCGAAGGAGGTGTTCTGCTGGTAA
TGGTCCGCGAGCTGCACGCTGCCGTCCTCGATGATGTGGCGGATCTTGACATTCACCTTGGTGCCGTTCTTCTGC
TTGCCGGCCATGAAAA

Control reverse run 1

CAGGAAAGCAAGGGGGAGGATTGGGAAGACAATAGCAGGCATGCTGGGGATGCGGTGGGCTCTATGGCTTCTGAG
GCGGAAAGAACCAGCTGGGGCTCTAGGGGGTATCCCCACGCGCCCTGTAGCGGCGCATTAAGCGCGGCGGGTGTG
GTGGTTACGCGCAGCGTGACCGCTACACTTGCCAGCGCCCTAGCGCCCGCTCCTTTCGCTTTCTTCCCTTCCTTT
CTCGCCACGTTCGCCGGCTTTCCCCGTCAAGCTCTAAATCGGGGGCTCCCTTTAGGGTTCCGATTTAGTGCTTTA
CGGCACCTCGACCCCAAAAAACTTGATTAGGGTGATGGTTCACGTACCTAGAAGTTCCTATTCCGAAGTTCCTAT
TCTCTAGATACCGGGTAGGGGAGGCGCTTTTCCCAAGGCAGTCTGGAGCATGCGCTTTAGCAGCCCCGCTGGGCA
CTTGGCGCTACACAAGTGGCCTCTGGCCTCGCACACATTCCACATCCACCGGTAGGCGCCAACCGGCTCCGTTCT
TTGGTGGCCCCTTCGCGCCACCTTCTACTCCTCCCCTAGTCAGGAAGTTCCCCCCCGCCCCGCAGCTCGCGTCGT
GCAGGACGTGACAAATGGAAGTAGCACGTCTCACTAGTCTCGTGCAGATGGACAGCACCGCTGAGCAATGGAAGC
GGGTAGGCCTTTGGGGCAGCGGCCAATAGCAGCTTTGCTCCTTCGCTTTCTGGGCTCAGAGGCTGGGAAGGGGTG
GGTCCGGGGGCGGGCTCAGGGGCGGGCTCAGGGGCGGGGCGGGCGCCCGAAGGTCCTCCGGAGGCCCGGCATTCT
GCACGCTTCAAAAGCGCACGTCTGCCGCGCTGTTCTCCTCTTCCTCATCTCCGGGCCTTTCGACCGATCCAGCCG
CCACCATGAAAAAGCCTGAACTCACCGCGACGTCTGTCGAGAAGTTTCTGATCGAAAAGTTCGACAGCGTCTCCG
ACCTGATGCAGCTCTCGGAGGGCGAAGAATCTCGTGCTTTCAGCTTCGATGTAGGAGGGCGTGGATATGTCCTGC
GGGTAAATAGCTGCGCCGATGGTTTCTACAAAGATCGTTATGTTTATCGGCACTTTGCATCGGCCGCGCTCCCGA
TTCCGGAAATGCTTGAAATTGGGGAATTCA

Control reverse run 2

CCCGGGGGGGGTGGGCAGGAACGCAAGGGGGAGGATTGGGAAGACATAGCAGGCATGCTGGGGATGCGGTGGGCT
CTATGGCTTCTGAGGCGGAAAGAACCAGCTGGGGCTCTAGGGGGTATCCCCACGCGCCCTGTAGCGGCGCATTAA
GCGCGGCGGGTGTGGTGGTTACGCGCAGCGTGACCGCTACACTTGCCAGCGCCCTAGCGCCCGCTCCTTTCGCTT
TCTTCCCTTCCTTTCTCGCCACGTTCGCCGGCTTTCCCCGTCAAGCTCTAAATCGGGGGCTCCCTTTAGGGTTCC
GATTTAGTGCTTTACGGCACCTCGACCCCAAAAAACTTGATTAGGGTGATGGTTCACGTACCTAGAAGTTCCTAT
TCCGAAGTTCCTATTCTCTAGATACCGGGTAGGGGAGGCGCTTTTCCCAAGGCAGTCTGGAGCATGCGCTTTAGC
AGCCCCGCTGGGCACTTGGCGCTACACAAGTGGCCTCTGGCCTCGCACACATTCCACATCCACCGGTAGGCGCCA
ACCGGCTCCGTTCTTTGGTGGCCCCTTCGCGCCACCTTCTACTCCTCCCCTAGTCAGGAAGTTCCCCCCCGCCCC
GCAGCTCGCGTCGTGCAGGACGTGACAAATGGAAGTAGCACGTCTCACTAGTCTCGTGCAGATGGACAGCACCGC
TGAGCAATGGAAGCGGGTAGGCCTTTGGGGCAGCGGCCAATAGCAGCTTTGCTCCTTCGCTTTCTGGGCTCAGAG
GCTGGGAAGGGGTGGGTCCGGGGGCGGGCTCAGGGGCGGGCTCAGGGGCGGGGCGGGCGCCCGAAGGTCCTCCGG
AGGCCCGGCATTCTGCACGCTTCAAAAGCGCACGTCTGCCGCGCTGTTCTCCTCTTCCTCATCTCCGGGCCTTTC
GACCGATCCAGCCGCCACCATGAAAAAGCCTGAACTCACCGCGACGTCTGTCGAGAAGTTTCTGATCGAAAAGTT
CGACAGCGTCTCCGACCTGATGCAGCTCTCGGAGGGCGAAGAATCTCGTGCTTTCAGCTTCGATGTAGGAGGGCG
TGGATATGTCCTGCGGGTAAATAGCTGCGCCGATGGTTTCTACAAAGATCGTTATGTTTATCGGCACTTTGCATC
GGCCGCGCTCCCGATTCCGGAAGTGCTTGACATTGGGGAGTTCAGCGAGAGCCTGACCTATTGCATCTCCCGCCG
TGCCAAGGGTGTCACGTTGCAAGACCTGCCTGAAACCAACTGCCCGCTGTTCTGCACCCGGTCCCGGAAGCCATG
GATGCAATCCCTGCACCCAATCTTAACCAGACAAGCGGGTTCGGCCCATTCGAACCAAAGGAATCGGCCAATACC
CCAAGGGGGGGGTTTTTTTGGCGGATGGGTGATCCCCCGG

Cu-DPA-TFO reverse run 1

CGTCCCCCACAACAACAAACATTCGCTGGCCTAACATAAGCAGGGAACAATCTTCTTTCTCATCCTCGGCCACTG
TTATTTATGGACGAGTTGCGGGCGGGGTCGTGGTTCCGGGACCCCTCATGTTCGGTGTGGATTTTGGGTGCTCAT
GGTCACCGGAGAGTCTAATTCGCCTGGTGACCCTGCTACCCTGCCGTGCGGAGGGCAGATATGCCTTAACCATGG
GCAGTATCGGTGGGTGACGTCAGTTTTGAACCCAACAGGTTGAAACACCTGCCGACTAACCACCTGATGACACTC
CTGGCCATATCAGCCCCCTACCCTATGTTCGTGTAATTGTGATCGAACTCCCATGAGAGATTATCTGTCTTTAGT
GGACAACTGCGACCTTGCGTTTTGGATATTGCCTGGACAACACTCTCTCTTTTTGTTTGGTTGTGCATTGGTTGT
CGTTTTATGGAGAATATCTAATGGTCCCGCAGTTTGTTGTTTG

Cu-DPA-TFO reverse run 2

AACCGAGGTGGGTGTGGTGGTTACGCCAAAAAGGACCTAGTCCTCACGCAGGTGATGATCCGCAGATAATTTCAC
CTGGTTGAACTACTCCTGTGCGGCATGAATGTGGTGATTAAAGCAAAATACGAAGTTAAGCAAAGATAGCATCCA
GGCGCATTTCGTGCCGAGAGATTGCCTCACCGGATATGCCCAAGAACACCTTCCGGGGGGGCCGCGCGGGATTTA
AGCCGGACCTGTTCTTTTATGACTTTGCTCATTGGCCTCCCCTCCCAGATTATCTGATCTATTAAAAGATCTATT
GGCAAGCAGAGGGCTCGCAGCTGACTAAATGATAAGATCCCTATACCGTTCACTCTATCACTTTTCAATTTCTTG
AAGGACAACACTGGGAACGATACAATTCCTTTGACAAACCACCTCCAATCCTACAGCGCCATCCAGTGAATGGCA
AGGCGTGCCGCATACGAACGCGTCGGCTCAAAAACTTGCCAGCACTGCTACAATGCTCTTCACGCTCACGGCGTT
CACAGAAGGTCCTGAAACGGATAACGACATTCATGACCGGAGCTGGGATTTGATTATCGTGACCACGCTATCTAC
ACCGGGCCATAGACGGGCAAGTACATTGAGACTCTCGCCCGCGCAGAGGAGAGGCCTCCGTCCCGTTTCATTCCA
TTCCCTGTTGTCAAACAAGAGCTGAGGGATGAGGTGCGACCCCTGAGGGAGGGGGTG

DPQ-Cu-DPA-TFO reverse run 1

CCACTTAAAAAAAAAAACCTAGCCTGTCTCATGATCTTCAGTATTTGGGTATGTTTGAATTTGTCAATAATAGTA
AAGATTGTCACTCTTTTACAGGAGGCAATAACAGATGGCTTGGAAATTGTGGTTTCACCTACAAGTCTACACAGT
GAATTAATGTGCCCATGTTGTTTGGATATGTGAATCCAAACGATGGCTACAATCGAGTGTGCACGGATTGTTTTC
TCGGATCCCTTAATCATGGGCCTGTTGGATTGGTTTATTACTCTATCTTTATACTACATCCTAACACTGCTGTCT
GGAACCGAACTCAGAAGTATGATAACATGTAAGACTTATAAAATTCTTCGACTCCCCGTCCTTAATCCCTACATA
AAGAAGACGGCGGTCAACACCAGCACACATCCCCGCGTTACTTTGACAAAACCCGCCCACCCTCTAACTATTTGT
TTGAAGTACCCCTGTTTTCGCTGCACTGCACTTAAATAACGTTTCTAATTTCGTTGA

DPQ-Cu-DPA-TFO reverse run 2

CTAAAGAGTCATACAGCGCAGGGGAGGATTGGGAGACAATAGCAGGCATGCTGGGGATGCGGTGGGCTCTATGGC
TTCTGAGGCGGAAAGAACCAGCTGGGGCTCTACGGGGAGTCCCCACGCGCCCTGGAGGGAAGCCGGGTGCCTGAT

GATTTCTGGATCCCGTTCGCGGTCATTCCCGAGGGCTCCCAGAGAGGTGAGTGGTGGGAGTGAGAAGACTGACTT
AAAAAAGTGCAGTGCTACCCCTGAGGCACTAAGGTTGGGTCCAGTTAAAACGGTAAACTTGGTGCTAAGCAGAAT
TAGGGCCTCAGCCGGGTGAAGAGGATGGCATCTTGAAGTTAAGACTTGAATTTTCCCTCTCTACATCTCCGCAAG
AATCCATCTTACAACAACGGAATTGACACCTGCTCTTCGGGGACCGTGTACCTGTCTATGCCACTCACATACTCT
AGAGGGAAAAA

DPPZ-Cu-DPA-TFO reverse run 1

AAACAAAACCCAGGCCAAGCAAAGGTCCAGATACATCCTCACTAAACGAATCCTGCTGGAAGCGAGCGGGACCCG
TAGAAACCTGGGAGAAGTGGTGAGACTGGGAATTTTCTGCCCAGACTGTCGGCATCCTGGCCGGCAGCCGATGAT
GCGCCGGGGGACTGCCCCTGTACCCCATAACTGCCAGTTCGCCCTTGAATAACCTTGGGTGAGAGATTCGATCAG
GCGGGATTTCCGCGCGTGAAGGTATCCTGACGAGCAAGGCGGTCAGCATTGACGGGCATGGCCGGCATGTGCCCG
TGCTGGATCACCTCTCCCAATGGCACCACTTTGCCGGTTTCTACAGCGTCAGCCGGGAAAGGCTCTCCTGCTATC
TCATGATTTTGGGGATACGATTGGTCGAAACTCCTCGCTGCCGACACGCTGTAGACGGGCGCGGCAAGACTGCCC
GGGGCCTGCGGCATTGAGTAGGTCCGCCCCACTTGACGGTGTGGCCTTAACTTACACTAAAGTGATAGCAGATGA
TCCCTAGCCCAACAAGATGACACAATGCAGGACCAGTTCGGCAGCAACAGCTCCGAGTTGCCTGAAGCAATCATG
TAGTGATGCACTTCATGCGAGCCCTTCTGCCATCGTTAATTGGGGTTCGTCCTATTCGGAAAAAA

DPPZ-Cu-DPA-TFO reverse run 2

GCCAAGCAAAGGTCCAGATACATCCTCACTAAACGAATCCTGCTGGAAGCGAGCGGGACCCGTAGAAACCTGGGA
GAAGTGGTGAGACTGGGAATTTTCTGCCCAGACTGTCGGCATCCTGGCCGGCAGCCGATGATGCGCCGGGGGACT
GCCCCTGTACCCCATAACTGCCAGTTCGCCCTTGAATAACAGGTCCCTTGGGTGAGAGATTCGATCAGGCGGGAT
TTCCGCGCGTGAAGGTATCCTGACGAGCAAGGCGGTCAGCATTGACGGGCATGGCCGGCATGTGCCCCTGGATCA
CCTCTCCCAATGGCACCACTTTGCCGGTTTCTACAGCGTCAGCCGGGAATCCTGCTATCTCATGATTTTGGGGAT
ACGATTGGTCGAAACTCCTCGCTGCCGACACGCTGTAGACGGGCGCGGCAAGACTGCAAAGGGGCCTGCGGCATT
GAGTAGGTCCGCCCCACTTGACGGTGTGGCCTTAACTTACACTAAAGTGATAGCAGATGATCCCTAGCCCAACAA
GATGACACAATGCAGTTCGGCAGCAACAGCTCCGAGTTGCCTGAAGCAATCATGTAGTGATGCACTTCATGCGAG
CCCTTCTGCCATCGTTAATTGGGGTTCGTCCTATTCGGAAAAAA
